# Viability and Outcomes With Revascularization or Medical Therapy in Ischemic Ventricular Dysfunction: A Prespecified Secondary Analysis of the REVIVED-BCIS2 Trial

**DOI:** 10.1001/jamacardio.2023.3803

**Published:** 2023-10-25

**Authors:** Divaka Perera, Matthew Ryan, Holly P. Morgan, John P. Greenwood, Mark C. Petrie, Matthew Dodd, Roshan Weerackody, Peter D. O’Kane, Pier Giorgio Masci, Muhummad Sohaib Nazir, Alexandros Papachristidis, Navtej Chahal, Rajdeep Khattar, Saad M. Ezad, Stam Kapetanakis, Lana J. Dixon, Kalpa De Silva, Adam K. McDiarmid, Michael S. Marber, Theresa McDonagh, Gerry P. McCann, Tim C. Clayton, Roxy Senior, Amedeo Chiribiri

**Affiliations:** 1 British Heart Foundation Centre of Research Excellence at the School of Cardiovascular and Metabolic Medicine & Sciences, King’s College London, London, United Kingdom; 2 Guy’s and St Thomas’ NHS Foundation Trust, London, United Kingdom; 3 Leeds Institute for Cardiometabolic Medicine, University of Leeds, Leeds, United Kingdom; 4 Institute of Cardiovascular and Medical Sciences, University of Glasgow, Glasgow, United Kingdom; 5 London School of Hygiene & Tropical Medicine, London, United Kingdom; 6 Barts Health NHS Trust, London, United Kingdom; 7 University Hospitals Dorset NHS Foundation Trust, Bournemouth, United Kingdom; 8 School of Biomedical Engineering and Imaging Sciences, King’s College London, London, United Kingdom; 9 Royal Brompton Hospital, London, United Kingdom; 10 King’s College Hospital NHS Foundation Trust, London, United Kingdom; 11 London Northwest Health NHS Trust, London, United Kingdom; 12 Belfast Health and Social Care NHS Trust, Belfast, United Kingdom; 13 University Hospitals Bristol NHS Foundation Trust, Bristol, United Kingdom; 14 Newcastle Hospitals NHS Foundation Trust, Newcastle, United Kingdom; 15 University of Leicester and the NIHR Leicester Biomedical Research Centre, Leicester, United Kingdom

## Abstract

**Importance:**

In the Revascularization for Ischemic Ventricular Dysfunction (REVIVED-BCIS2) trial, percutaneous coronary intervention (PCI) did not improve outcomes for patients with ischemic left ventricular dysfunction. Whether myocardial viability testing had prognostic utility for these patients or identified a subpopulation who may benefit from PCI remained unclear.

**Objective:**

To determine the effect of the extent of viable and nonviable myocardium on the effectiveness of PCI, prognosis, and improvement in left ventricular function.

**Design, Setting, and Participants:**

Prospective open-label randomized clinical trial recruiting between August 28, 2013, and March 19, 2020, with a median follow-up of 3.4 years (IQR, 2.3-5.0 years). A total of 40 secondary and tertiary care centers in the United Kingdom were included. Of 700 randomly assigned patients, 610 with left ventricular ejection fraction less than or equal to 35%, extensive coronary artery disease, and evidence of viability in at least 4 myocardial segments that were dysfunctional at rest and who underwent blinded core laboratory viability characterization were included. Data analysis was conducted from March 31, 2022, to May 1, 2023.

**Intervention:**

Percutaneous coronary intervention in addition to optimal medical therapy.

**Main Outcomes and Measures:**

Blinded core laboratory analysis was performed of cardiac magnetic resonance imaging scans and dobutamine stress echocardiograms to quantify the extent of viable and nonviable myocardium, expressed as an absolute percentage of left ventricular mass. The primary outcome of this subgroup analysis was the composite of all-cause death or hospitalization for heart failure. Secondary outcomes were all-cause death, cardiovascular death, hospitalization for heart failure, and improved left ventricular function at 6 months.

**Results:**

The mean (SD) age of the participants was 69.3 (9.0) years. In the PCI group, 258 (87%) were male, and in the optimal medical therapy group, 277 (88%) were male. The primary outcome occurred in 107 of 295 participants assigned to PCI and 114 of 315 participants assigned to optimal medical therapy alone. There was no interaction between the extent of viable or nonviable myocardium and the effect of PCI on the primary or any secondary outcome. Across the study population, the extent of viable myocardium was not associated with the primary outcome (hazard ratio per 10% increase, 0.98; 95% CI, 0.93-1.04) or any secondary outcome. The extent of nonviable myocardium was associated with the primary outcome (hazard ratio, 1.07; 95% CI, 1.00-1.15), all-cause death, cardiovascular death, and improvement in left ventricular function.

**Conclusions and Relevance:**

This study found that viability testing does not identify patients with ischemic cardiomyopathy who benefit from PCI. The extent of nonviable myocardium, but not the extent of viable myocardium, is associated with event-free survival and likelihood of improvement of left ventricular function.

**Trial Registration:**

ClinicalTrials.gov Identifier: NCT01920048

## Key Points

**Question:**

Does myocardial viability testing identify patients with ischemic left ventricular dysfunction who benefit from percutaneous coronary intervention?

**Findings:**

In this prespecified subgroup analysis of a randomized clinical trial of 610 participants with ischemic left ventricular dysfunction 35% or less, myocardial viability testing with cardiovascular magnetic resonance imaging or stress echocardiography did not identify a population of patients who benefit from percutaneous coronary intervention. The extent of nonviable myocardium was associated with a higher risk of death or hospitalization for heart failure and a lower chance of improvement in left ventricular function.

**Meaning:**

Findings suggest that the extent of dysfunctional yet viable myocardium was not associated with revascularization outcomes.

## Teaser

This secondary analysis of a randomized clinical trial assesses whether myocardial viability testing identifies patients with ischemic left ventricular dysfunction who benefit from percutaneous coronary intervention.

## Introduction

Myocardial viability tests are thought to identify patients with ischemic cardiomyopathy who benefit from revascularization. These tests typically characterize myocardial tissue into 3 distinct states: healthy myocardium contracting normally at rest, viable or hibernating myocardium that contracts abnormally at rest where improvement in function is expected, and nonviable scarred myocardium that contracts abnormally at rest but where improvement is not expected. Historically, viability has been regarded in a binary manner, and when classified in this way, observational, nonrandomized data suggest that patients with extensive myocardial viability might experience left ventricular recovery and improved survival after revascularization.^1^ However, when treatment was by random allocation in the Surgical Treatment for Ischemic Heart Failure (STICH) trial, no interaction was found between viability status and the effect of coronary artery bypass graft surgery.^2^ Other observational studies that regarded viability as a continuum have suggested an incremental benefit of revascularization above medical therapy alone, although interpretation of these data is limited by their retrospective nature and nonrandomized treatment allocation.^3^ Hence, it remains unclear whether myocardial viability is correlated with event-free survival or left ventricular recovery and which viability characteristics are associated with the effect of revascularization on these outcomes.^4^

We recently completed the Revascularization for Ischemic Ventricular Dysfunction (REVIVED-BCIS2) trial, a randomized comparison of percutaneous coronary intervention (PCI) vs optimal medical therapy (OMT) alone for patients with ischemic cardiomyopathy who had undergone mandatory viability testing. We report the prespecified analysis of clinical and left ventricular outcomes in relation to the extent of viable and nonviable myocardium to determine their associations with prognosis and functional recovery and the interaction with revascularization.

## Methods

REVIVED-BCIS2 was a prospective, multicenter, open-label randomized clinical trial, the design and preliminary results of which have been published previously.^5,6^ Participants for this subgroup analysis were recruited from 40 sites in the United Kingdom between August 28, 2013, and March 19, 2020 (eAppendixes 1 and 2 in Supplement 2). The trial protocol received ethical approval from the UK Health Research Authority, was registered before enrollment of the first participant (NCT01920048), and is available in Supplement 1. All participants provided written informed consent. This study conforms to the Consolidated Standards of Reporting Trials (CONSORT) guideline for reporting of randomized clinical trials.

Participants were eligible for enrollment if they had a left ventricular ejection fraction less than or equal to 35%, extensive coronary artery disease (British Cardiovascular Intervention Society jeopardy score ≥6),^7^ and evidence of myocardial viability. The qualifying threshold for viability was defined as at least 4 myocardial segments that were dysfunctional at rest, judged by recruiting centers to be viable and supplied by coronary arteries that were severely diseased but amenable to revascularization by PCI. Key exclusion criteria were myocardial infarction fewer than 4 weeks before randomization, decompensated heart failure, and sustained ventricular tachycardia or ventricular fibrillation less than 72 hours before randomization. Participants were randomized in a 1:1 ratio to a strategy of either PCI plus OMT (PCI group) or OMT alone (OMT group) via an online randomization system (Sealed Envelope).^8^ All clinical outcomes were adjudicated by an independent clinical events committee, and left ventricular ejection fraction was measured by an independent echocardiography core laboratory blinded to treatment assignment, outcome data, and the temporal sequence of scans.^6^

Viability assessment could be obtained by cardiovascular magnetic resonance (CMR) imaging, dobutamine stress echocardiography, single-photon emission computed tomography, or positron emission tomography. For this analysis, participants who had viability assessed with CMR imaging or dobutamine stress echocardiography were included, with CMR imaging data used when both were available. Given the small number of participants assessed only by single-photon emission computed tomography or positron emission tomography, these participants were excluded because the results would not be generalizable to these nuclear imaging techniques. Any participants for whom viability study results could not be obtained or who were unsuitable for core laboratory analysis were also excluded.

All available CMR imaging and dobutamine stress echocardiography studies were analyzed by independent core laboratories (CMR imaging core laboratory at King’s College London, United Kingdom, and dobutamine stress echocardiography core laboratory at King’s Health Partners, United Kingdom). The left ventricle was described with a 17-segment American Heart Association model.^9^ Segmental wall motion was classed as normal or dysfunctional, with dysfunctional myocardial segments classified as viable or nonviable based on a 25% late gadolinium enhancement transmural threshold by CMR imaging or the presence of contractile reserve by dobutamine stress echocardiography (Table 1).^10,11^ Per-participant viability status was described by the proportion of segments that were viable and nonviable; segments with nonischemic scarring were excluded from the analysis. A sensitivity analysis was performed, with segmental viability and nonviability adjudicated using a 50% late gadolinium enhancement transmural threshold.

**Table 1.** Characterization of Myocardial Viability

Viability definition	Wall motion^a^	CMR–transmurality of enhancement	DSE–contractile reserve^b^
**Segmental classification by CMR or DSE**			
Normal	Normal	NA	NA
Viable	Dysfunctional	≤25%^c^	Present
Nonviable	Dysfunctional	>25%^c^	Absent
**Participant-level classification by CMR** ^d^			
Scar burden (% LV)	Each segment was classified by transmural extent of LGE as 0%, 1%-25%, 26%-50%, 51%-75%, or 76%-100%.^10^ LGE was summed across all segments and expressed as a proportion of the LV.^e^		

Abbreviations: CMR, cardiovascular magnetic resonance imaging; DSE, dobutamine stress echocardiography; LGE, late gadolinium enhancement; LV, left ventricular myocardial volume; NA, not applicable.

^a^ Myocardial wall motion was graded on a 5-point scale as normal, hypokinetic, akinetic, dyskinetic, or aneurysmal.

^b^ Contractile reserve was defined as an improvement in wall motion score greater than or equal to 1 or greater than or equal to 2 if the segment was dyskinetic at rest.

^c^ Sensitivity analyses were performed for an LGE threshold of less than or equal to 50%.

^d^ When calculating the extent of viable and nonviable myocardium at a participant level, segments with a nonischemic scar were excluded from the numerator; the denominator was all segments.

^e^ Segmental LGE was calculated as the midpoint in each range (for instance, 13% for the range 1%-25%).

In the CMR imaging cohort, per-participant ischemic scar burden was determined semiquantitatively by visual consensus of expert readers in pairs (P.G.M., M.S.N., and A.C.) and expressed as a percentage of the total left ventricular myocardial volume (Table 1). This determination included all myocardial segments regardless of resting wall motion, although segments with clearly nonischemic late gadolinium enhancement were excluded.

The primary outcome was a composite of all-cause death or hospitalization for heart failure during a minimum follow-up period of 24 months. Secondary outcomes were all-cause death, cardiovascular death, hospitalization for heart failure, and improvement in left ventricular function at 6 months, defined as a greater than the median absolute change in left ventricular ejection fraction from baseline, detected by echocardiography, measured by a blinded core laboratory at Guy’s and St Thomas’ NHS Foundation Trust.

### Statistical Analysis

The statistical analysis plan was finalized before unblinding of viability data. A formal power calculation was not performed for this secondary analysis. A Cox proportional hazards model was used to assess the association between the extent of viable myocardium, nonviable myocardium, scar burden, and the primary outcome across the whole population, adjusted for baseline factors, including age, sex, previous heart failure hospitalization, presence of diabetes, chronic kidney failure, left ventricular ejection fraction, extent of coronary disease, and the modality of viability testing. The interaction between randomized assignment, independent variables (the extent of viable myocardium, nonviable myocardium, and scar burden), and major outcomes was assessed with a Cox proportional hazards model containing the following covariates: viability characteristics (treated as a linear effect), assigned treatment, their interaction, and baseline risk factors. The results were calculated by considering each viability characteristic as a continuous variable (expressed as hazard ratios [HRs] and 95% CIs), but for illustrative purposes, Kaplan-Meier curves and forest plots were stratified by tertiles of these parameters. Logistic regression models were also created to explore the association between viability characteristics and improvement in left ventricular function, defined dichotomously by the median change in left ventricular ejection fraction adjusting for baseline variables.

Finally, a landmark analysis was performed including participants who survived at least 6 months from randomization to test the association between improvement in left ventricular function and the primary outcome, using Cox proportional models. Missing values of left ventricular ejection fraction were imputed with a multiple imputation model with chained equations that included randomized treatment, age, sex, and baseline, 6-month, and 12-month left ventricular ejection fractions. A sensitivity analysis was performed and was restricted to observed values, without imputation. All analyses were conducted with Stata, version 17.0 (StataCorp LLC), from March 31, 2022, to May 1, 2023. Two-sided Wald tests were used to calculate *P* values, with *P* < .05 used to indicate statistical significance.

## Results

**Figure 1.**
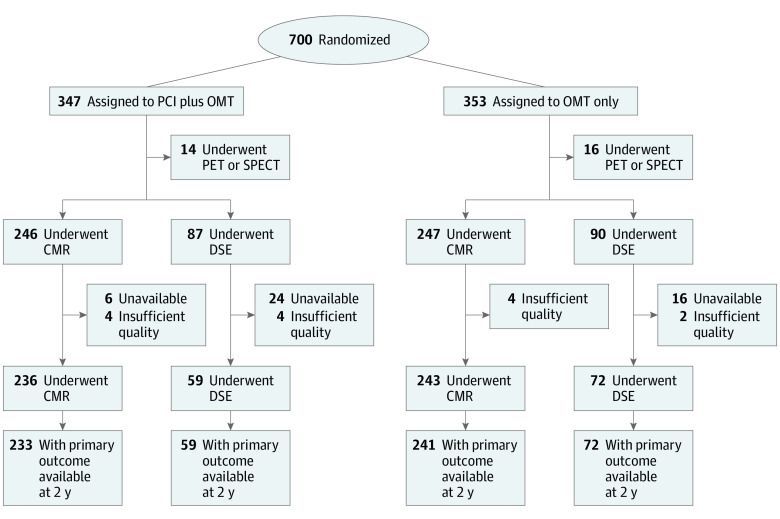
CONSORT Diagram Showing Flow of Participants Through the Study CMR indicates cardiovascular magnetic resonance imaging; DSE, dobutamine stress echocardiography; OMT, optimal medical therapy; PCI, percutaneous coronary intervention; PET, positron emission tomography; and SPECT, single-photon emission computed tomography.

Of the 700 participants randomized in the REVIVED-BCIS2 trial, 610 were included in this prespecified analysis, 295 assigned to the PCI group and 315 to the OMT group (Figure 1). The mean (SD) age of the participants was 69.3 (9.0) years. In the PCI group, 258 (87%) were male, and 37 (13%) were female; in the OMT group, 277 (88%) were male, and 38 (12%) were female. Race and ethnicity were self-reported by participants using options defined by the investigators. Participants were asked to select their ethnicity as Asian, Black, White, other, or prefer not to say. No further definition was provided. In the PCI group vs OMT group, 26 (9%) vs 13 (4%) were Asian; 3 (1%) vs 3 (1%) were Black; 5 (2%) vs 3 (1%) were of other race and ethnicity (self-reported by participants, with these fields provided in the case report form), or race and ethnicity were not reported; and 261 (88%) vs 296 (94%) were White. The groups were balanced in relation to baseline clinical, demographic, and viability characteristics (Table 2). The median extent of viable and nonviable myocardium was 29% (IQR, 12% to 53%) and 29% (IQR, 12%-41%), respectively, across the whole trial population. The characteristics of participants undergoing CMR imaging or dobutamine stress echocardiography and those who were not included in this analysis were similar (eTable 1 in Supplement 2).

**Table 2.** Demographic and Clinical Characteristics of the Participants at Baseline^a^

Characteristic	PCI (n = 295)	OMT (n = 315)
Age, mean (SD), y	69.8 (9.1)	68.8 (8.9)
Sex, No. (%)		
Male	258 (87)	277 (88)
Female	37 (13)	38 (12)
Diabetes, No. (%)	116 (39)	134 (43)
Race and ethnicity, No. (%)^b^		
Asian	26 (9)	13 (4)
Black	3 (1)	3 (1)
White	261 (88)	296 (94)
Other or not reported	5 (2)	3 (1)
History of myocardial infarction, No. (%)	146 (49)	175 (56)
Hospitalization for heart failure in prior 2 y, No. (%)	104 (36)	102 (32)
Cardiac medication, No. (%)		
RAAS inhibitor	258 (87)	282 (90)
β-Blocker	266 (90)	285 (90)
Mineralocorticoid receptor antagonist	153 (52)	151 (48)
BCIS jeopardy score, median (IQR)^c^	10 (8-12)	10 (8-12)
ICD ± CRT at randomization, No. (%)	65 (22)	58 (18)
Left main coronary artery disease, No. (%)	46 (16)	40 (13)
Left ventricular ejection fraction, mean (SD), %^d^	32 (10)	32 (10)
Viability test, No. (%)^e^		
CMR	236 (80)	243 (77)
DSE	59 (20)	72 (23)
Extent of viable myocardium, median (IQR), %	29 (18-53)	29 (12-47)
Extent of nonviable myocardium, median (IQR), %	29 (12-41)	29 (12-41)
Scar burden, median (IQR), %	19 (9-28)	18 (9-28)

Abbreviations: BCIS, British Cardiovascular Intervention Society; CMR, cardiovascular magnetic resonance imaging; CRT, cardiac resynchronization therapy; DSE, dobutamine stress echocardiography; ICD, implantable cardioverter-defibrillator; OMT, optimal medical therapy; PCI, percutaneous coronary intervention; RAAS, renin-angiotensin-aldosterone system.

^a^ Percentages may not total 100 because of rounding.

^b^ Self-reported by participants using options defined by the investigators. Participants were asked to select their ethnicity as Asian, Black, White, other, or prefer not to say. No further definition was provided.

^c^ The BCIS jeopardy score is a quantification of the extent of myocardial jeopardy relating to clinically significant coronary artery stenoses. The score ranges from 0 (no significant coronary disease) to 12 (disease jeopardizing the whole left ventricular myocardium).

^d^ Baseline left ventricular ejection fraction measured by the blinded echocardiography core laboratory.

^e^ Sixteen participants of 295 (5.4%) in the PCI group and 19 participants of 315 (6.0%) in the OMT group had nonischemic scar. The median number of segments with nonischemic scar in these participants was 2 segments (IQR, 1-3 segments) in the PCI group and 2 segments (IQR, 1-3 segments) in the OMT group.

A primary outcome event occurred for 107 of 295 participants in the PCI group and 114 of 315 participants in the OMT group (36.3% vs 36.2%; difference between groups, 0.1%; HR, 0.99; 95% CI, 0.76-1.29; *P* = .93) at a median of 3.4 years (IQR, 2.3-5.0 years), consistent with the results in the whole trial population (eTable 2 in Supplement 2).

**Figure 2.**
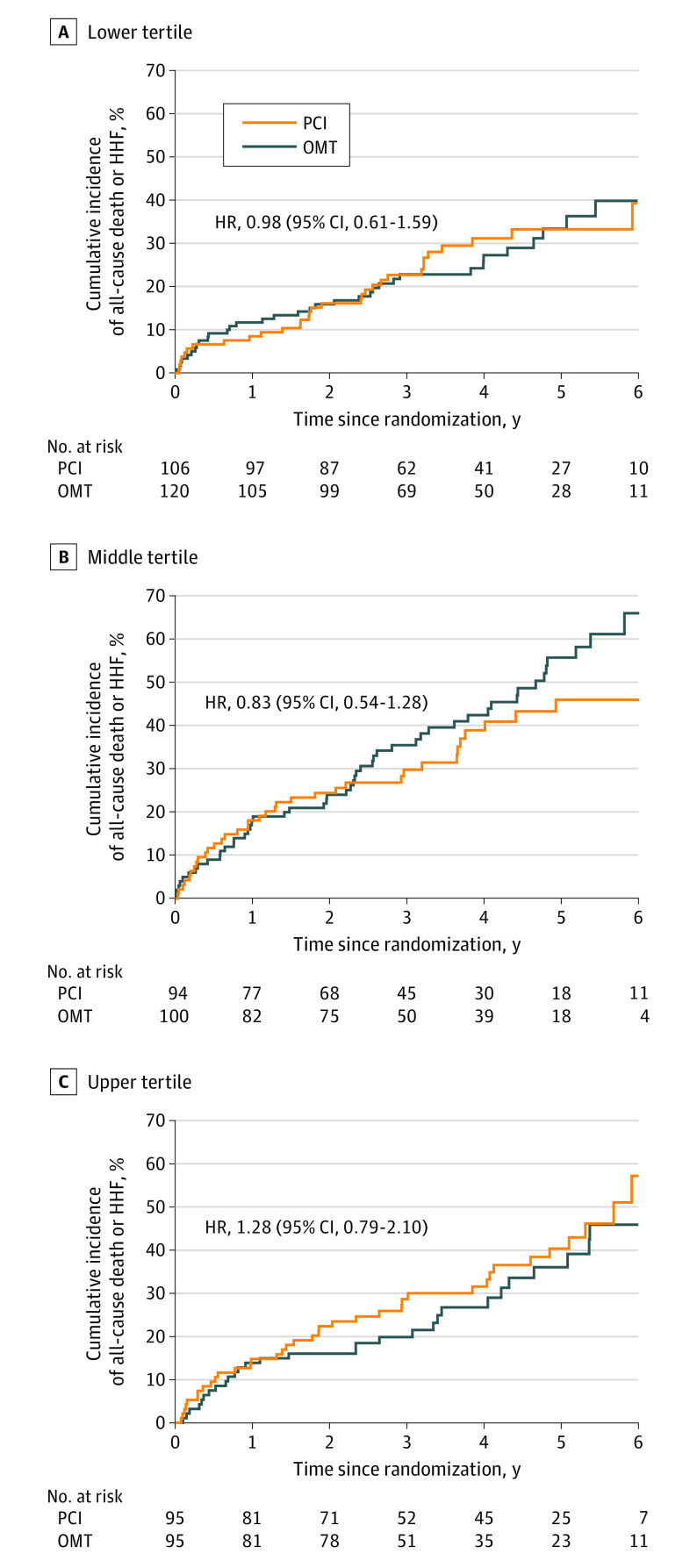
All-Cause Death or Hospitalization for Heart Failure (HHF) in Participants Assigned to Percutaneous Coronary Intervention (PCI) or Optimal Medical Therapy (OMT), Stratified by Viability Tertile Kaplan-Meier estimates of the cumulative incidence of death from any cause of HHF in a time-to-first-event analysis, stratified by tertiles of the extent of myocardial viability. A, For the lower tertile, the extent of viability was less than or equal to 18%. B, For the middle tertile, the extent of viability was greater than 18% to less than or equal to 41%. C, For the upper tertile, the extent of viability was greater than 41%. HR indicates hazard ratio.

There was no evidence of an interaction between the extent of viable myocardium and the effect of assignment to PCI vs OMT on occurrence of the primary outcome or any of the secondary outcomes (Figure 2; eFigures 1 and 2 and eTable 3 in Supplement 2). Similarly, there was no evidence of an interaction between the extent of nonviable myocardium and the effect of assignment to PCI vs OMT on occurrence of the primary outcome or any of the secondary outcomes (eFigures 1 and 2 and eTable 3 in Supplement 2).

**Figure 3.**
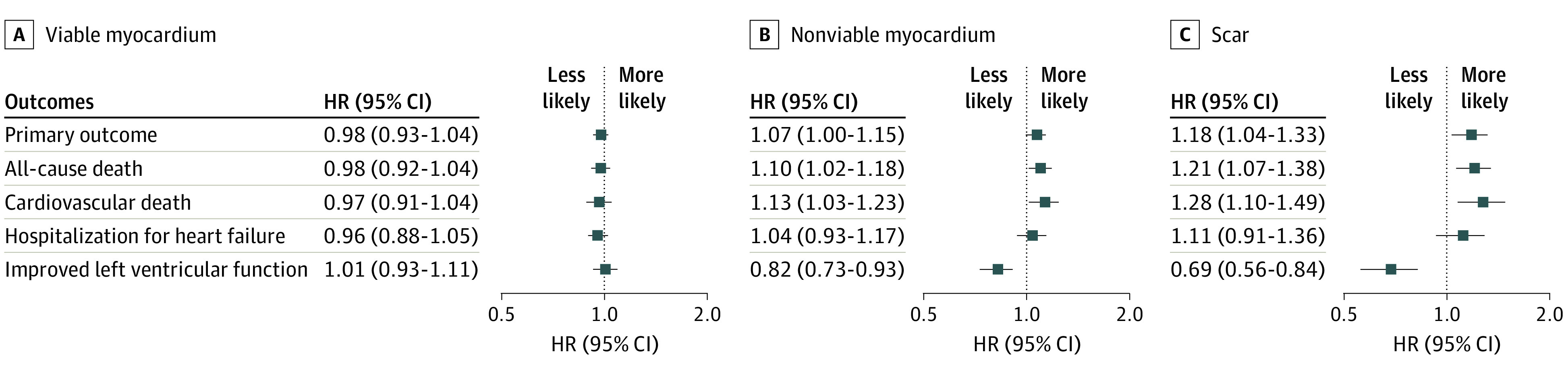
Association Between Viability Characteristics and Trial Outcomes Forest plot of the hazard ratio (HR) (for clinical outcomes) or odds ratio (for improvement in left ventricular function) for the primary and secondary outcomes according to the extent of viable myocardium, extent of nonviable myocardium, and scar burden. Data relate to the whole trial population. Ratios are expressed per 10% absolute increase in the characteristic relative to overall left ventricular mass. The values relating to this graph are reported in eTable 5 in Supplement 2. HR indicates hazard ratio.

Across the trial population, no association was observed between the extent of viable myocardium and occurrence of the primary outcome (HR per 10% absolute increase in viable myocardium, 0.98; 95% CI, 0.93-1.04; *P* = .56) (Figure 3; eTable 4 in Supplement 1) or any of the secondary outcomes. In contrast, an increasing volume of nonviable myocardium was associated with a greater likelihood of the primary outcome (HR per 10% absolute increase in nonviable myocardium, 1.07; 95% CI, 1.00-1.15; *P* = .048) (Figure 3; eTable 4 in Supplement 2). Results were consistent for all-cause death and cardiovascular death (HR for viable myocardium: all-cause death, 0.98 [95% CI, 0.92-1.04], cardiovascular death, 0.97 [95% CI, 0.91-1.04]; HR for nonviable myocardium: all-cause death, 1.10 [95% CI, 1.02-1.18], cardiovascular death, 1.13 [95% CI, 1.03-1.23]; and HR for scar burden: all-cause death, 1.21 [95% CI, 1.07-1.38], cardiovascular death, 1.28 [95% CI, 1.10-1.49]), whereas no effect was observed on hospitalization for heart failure (eTable 4 in Supplement 2).

Sensitivity analyses based on a late gadolinium enhancement transmural threshold less than or equal to 50% also showed no association between the extent of viability and primary outcome, as well as no interaction with assignment to PCI vs OMT (eTable 5 in Supplement 2).

For the 479 participants assessed with CMR imaging, scar burden did not interact with the effect of assignment to PCI vs OMT on the risk of the primary outcome or any secondary outcomes (eFigures 1 and 2 and eTable 3 in Supplement 1). A greater scar burden was associated with an increased incidence of the primary outcome (HR per 10% absolute increase in scar burden, 1.18; 95% CI, 1.04-1.33; *P* = .009), all-cause death, and cardiovascular death across the whole trial population (Figure 3; eTable 4 in Supplement 2).

The median change in left ventricular ejection fraction was 4.7% (IQR, −2.2% to 12.5%) at 6 months (eTable 6 in Supplement 2). None of the viability characteristics interacted with the effect of assignment to PCI vs OMT on the likelihood of improvement in left ventricular function (eFigure 3 and eTable 7 in Supplement 2). In the whole trial population, the extent of viable myocardium was not associated with improvement in left ventricular function at 6 months (odds ratio, 1.01; 95% CI, 0.93-1.11; *P* = .78), but increasing volumes of nonviable myocardium (odds ratio, 0.82; 95% CI, 0.73-0.93; *P* = .002) and scar (odds ratio, 0.69; 95% CI, 0.56-0.84; *P* < .001) were associated with a lower likelihood of improvement in left ventricular function (eTable 4 in Supplement 2). The determinants of improvement in left ventricular function at 12 months were the same as at 6 months (eFigure 4 and eTable 7 in Supplement 2).

In the landmark analysis of participants surviving more than 6 months, improvement in left ventricular function by at least 4.7% was associated with a 38% relative risk reduction for the primary outcome compared with that of those who did not have an improvement (odds ratio, 0.62; 95% CI, 0.41-0.95) (eFigure 5 in Supplement 2). The association was maintained when improvement in left ventricular function at 6 months was regarded as a continuous variable (HR per 5% absolute improvement in ejection fraction, 0.87; 95% CI, 0.79-0.95; *P* = .003).

## Discussion

The REVIVED-BCIS2 trial showed that, compared with OMT alone, PCI neither reduced the occurrence of death or hospitalization for heart failure nor influenced the degree of left ventricular recovery in patients with severe ischemic left ventricular dysfunction. In this prespecified substudy, in which we carried out blinded core laboratory analysis of CMR imaging and dobutamine stress echocardiography viability tests performed before randomization, we did not find that any of the viability characteristics influenced the effect of PCI on either prognosis or likelihood of improvement in left ventricular function. Our findings do not support the use of myocardial viability testing to select patients with severe left ventricular systolic dysfunction for revascularization.

The traditional concept of myocardial hibernation, an adaptive state of decreased contractility that can be reversed by relieving the ischemic substrate through medical therapy and revascularization, appears at odds with our findings.^12,13,14^ Furthermore, although an increasing amount of hibernating myocardium has previously been associated with a worse prognosis, we did not find any association with all-cause or cardiovascular mortality.^3,15^ Several potential explanations need to be considered. The lack of association may be because contemporary viability testing merely demonstrates the absence of appreciable myonecrosis in regions that are dysfunctional but does not specifically detect myocardial hibernation.^16^ Alternatively, it is possible that the hibernation paradigm itself may need modification. Although ischemia may trigger the process of hibernation, revascularization may not be sufficient to effectively reverse it.^17^ The time taken to reverse hibernation has also been reported to be very variable,^14^ but given that the associations with 12-month left ventricular remodeling were similar to those at 6 months in our study and that clinical follow-up was continued for a median of 3.4 years (IQR, 2.3-5.0 years), length of follow-up is unlikely to have affected our findings.

In contrast, the extent of nonviable myocardium was associated with an increased likelihood of the primary outcome independent of whether participants were assigned to have revascularization or not. This effect was driven by increased mortality rather than more heart failure hospitalization, with a clear relationship between nonviable myocardial mass and cardiovascular death. When scar burden was semiquantitatively assessed on CMR imaging, agnostic to resting wall motion, the prognostic association was stronger. Whether the negative association between scar and event-free survival is mediated by an increased incidence of fatal ventricular arrhythmia, as well as whether scar burden and morphology could be used to stratify risk and guide management, warrants further investigation. Given that current international guidelines recommend that arrhythmic risk stratification be primarily based on left ventricular ejection fraction,^18^ it is notable that scar burden remained strongly associated with the incidence of the primary outcome after adjusting for baseline left ventricular ejection fraction.

Finally, our results demonstrate that patients who experience improvement in left ventricular function by 6 months have markedly better event-free survival than those who do not. Although this association has been reported in nonischemic left ventricular dysfunction,^19^ the STICH trial investigators did not find that improvement in left ventricular function affected survival.^20^ The discordance may be due to differences in trial methods because assessment of left ventricular function was protocol mandated for all participants in REVIVED-BCIS2 and continued to 12 months (rather than 4 months in STICH), as well as the observation that mean change in ejection fraction was lower in STICH, which may in turn reflect improvements in optimal medical and device therapy between the trials.

### Strengths and Limitations

Apart from mandated viability testing, randomized assignment to revascularization, and high rates of guideline-directed medical and device therapy, our study had 2 key strengths compared with previous observational data. First, we characterized participants in terms of viable and nonviable myocardium, each of which relates to a distinct pathophysiologic determinant of outcome in ischemic cardiomyopathy. Second, all these viability characteristics were analyzed as continuous rather than binary variables, which better captures biological heterogeneity and enhances our ability to detect potential interactions.

Our study does have some limitations. We used data from only 87% of the trial population, although the baseline characteristics and clinical outcomes were similar to those of the overall trial population, so this loss of data is unlikely to have affected the results. Enrollment in the REVIVED-BCIS2 trial required participants to have at least 4 segments of viable myocardium according to local adjudication, and consequently the exclusion of patients without viable myocardium means the results cannot be generalized to the entire viability continuum; however, given the consistency of our results with those of the STICH trial, it is unlikely that the primary findings would be affected. Participants for whom viability was assessed with positron emission tomography or single-photon emission computed tomography were excluded, and we cannot extrapolate the results to these modalities. The accuracy of CMR imaging–based scar measurement might be improved by quantitative analysis, but automated methods are not yet in widespread clinical use, and our method best reflects the current way in which CMR imaging studies are interpreted in this patient population. Because we did not mandate paired ischemia testing, it is not possible to link clinical outcomes and improvement in left ventricular function to change in ischemic burden (with medical therapy, PCI, or both), and hence any comments on the mechanisms of hibernation remain speculative. Finally, differentiating ischemic left ventricular dysfunction from nonischemic cardiomyopathy with bystander coronary artery disease can be challenging in the absence of a definitive test. This issue might influence the results, although the REVIVED-BCIS2 population was phenotyped with advanced cardiac imaging during viability testing and a threshold British Cardiovascular Intervention Society jeopardy score that is highly specific for ischemic left ventricular dysfunction.^21^

## Conclusions

In conclusion, in this subgroup analysis of a randomized clinical trial of PCI vs OMT alone, viability testing did not identify participants for whom PCI would confer a prognostic benefit or improve left ventricular function. In this population with ischemic left ventricular dysfunction, the extent of viable myocardium as estimated by CMR imaging or dobutamine stress echocardiography did not correlate with event-free survival or the likelihood of improvement in left ventricular function of 5% or greater, although the extent of nonviable myocardium (by CMR imaging or dobutamine stress echocardiography) and the total left ventricular scar burden (by CMR imaging) were associated with both outcomes.

## Notes

Supplement 1.
Trial Protocols, SAPs, and Viability Study

Supplement 2. **eAppendix 1.** Revived Sites and Investigators **eAppendix 2.** Trial Organization and Oversight **eFigure 1.** Relationship Between Viability Characteristics and the Primary Outcome by Treatment Assignment **eFigure 2.** Interaction Between Treatment Assignment, Viability Characteristics (in Tertiles) and Primary Outcome **eFigure 3.** Interaction Between Treatment Assignment, Viability Characteristics (in Tertiles) and Likelihood of Left Ventricular Improvement at 6-Months **eFigure 4.** Improvement in Left Ventricular Ejection Fraction at 12 Months **eFigure 5.** Impact of Improvement in Left Ventricular Function at 6 Months on Subsequent Occurrence of Primary Outcome (Landmark Analysis) **eTable 1.** Baseline Demographics in Patients Who Had CMR, DSE or Were Excluded **eTable 2.** Primary and Clinical Secondary Outcomes **eTable 3.** Interaction Between Treatment Assignment, Viability Characteristics (Continuous) and Outcomes **eTable 4.** Relationship Between Viability Characteristics (Continuous) and Outcomes **eTable 5.** Sensitivity Analysis (Incorporating 50% LGE Transmurality Threshold) of Interaction Between Treatment Assignment, Viability Characteristics (Continuous) and Primary Outcome **eTable 6.** Change in Left Ventricular Ejection Fraction From Baseline to 6- and 12-Month Follow-Up **eTable 7.** Determinants of Binary Improvement in Left Ventricular Ejection Fraction at 6- and 12-Months
eReferences.

Supplement 3.
Nonauthor Collaborators

Supplement 4.
Data Sharing Statement
