# Supplementary material for: Viability and Outcomes With Revascularization or Medical Therapy in Ischemic Ventricular Dysfunction: A Prespecified Secondary Analysis of the REVIVED-BCIS2 Trial
Source: JAMA Cardiol. 2023 Oct 25;8(12):1154–61. doi: 10.1001/jamacardio.2023.3803 (PMC10600721; doi:10.1001/jamacardio.2023.3803)
Supplement: Supplement 1. — Trial Protocols, SAPs, and Viability Study [file jamacardiol-e233803-s001.pdf]

REVascularisation for Ischaemic VEntricular Dysfunction

(REVIVED-BCIS2)

Trial Protocol Version 8

Sponsored by King's College London

Funded by NIHR HTA CET

Table of Contents

1. Trial Summary 5
1.1. Protocol Summary 5
1.2. Trial Flowchart 6
1.3. Trial Organisation 7
1.3.1. NIHR HTA CET Grant applicants 7
1.3.2. Trial Steering Committee (TSC) 7
1.3.3. Project Management Group (PMG) 7
1.3.4. Clinical Trials Unit (CTU) 8
1.3.5. Data and Safety Monitoring Committee (DSMC) 8
1.3.6. Clinical Events Committee (CEC) 8
1.3.7. Medical Therapy Committee 8
1.3.8. Recruiting Centres 8
2. Background 9
2.1. Epidemiology 9
2.2. Hibernating Myocardium 9
2.3. CABG surgery for ischaemic cardiomyopathy 10
2.4. PCI for ischaemic cardiomyopathy 11
3. Hypothesis 13
4. Endpoints 13
4.1. Primary Endpoint 13
4.2. Major Secondary Endpoints 13
4.3. Other Secondary Endpoints 13
4.4. Endpoint Definitions 14
5. Safety Reporting 17
5.1. Definition 17
5.2. Unexpected Serious Adverse Events 17
5.3. Unexpected Non-Serious Adverse Events 17
5.4. Reporting unexpected adverse events 17
5.4.1. Assessment of intensity 17
5.4.2. Assessment of causality 17
5.5. Notification 18
6. Trial Population 18
6.1. Inclusion Criteria 18

6.2. Exclusion Criteria ... 18

7. Ethical Considerations ... 19

7.1. Consent ... 19

7.2. Declaration of Helsinki and Good Clinical Practice ... 19

7.3. Ethical committee review ... 19

8. Statistical Considerations ... 19

8.1. Power Calculation ... 19

8.2. Crossover ... 20

8.3. Statistical Analysis ... 20

8.4. Interim Analysis ... 21

9. Screening ... 21

9.1. Screening population ... 21

9.2. Screening log ... 21

10. Assessment of LVEF ... 22

10.1. Qualifying ejection fraction ... 22

10.2. Baseline echocardiogram ... 22

10.3. Qualifying EF flowchart: ... 22

10.4. Patients with recent MI ... 23

10.5. Echo core lab ... 23

10.6. Angiography core lab ... 23

11. Assessment of Viability ... 23

12. Randomisation ... 24

13. Percutaneous Coronary Intervention ... 24

13.1. Adjunctive therapy and devices ... 24

13.2. Completeness of Revascularisation ... 24

13.3. Staged PCI ... 25

13.4. PCI Definitions ... 25

14. Optimal Medical Therapy ... 26

15. ICDs and Cardiac Resynchronisation ... 26

16. Data collection and follow-up ... 27

16.1. Tests required for eligibility ... 27

16.1.1. Time limits for screening tests ... 27

16.2. TRIAL CHECKLIST 28
16.3. DATA HANDLING 30
16.3.1. DATA COLLECTION 30
16.3.2. ADVERSE EVENTS 31
16.3.3. PARTICIPANT ID LOG 31
16.3.4. MORTALITY TRACKING 31
17. Health Economic Analysis 31
18. References 33
Appendix 1: Glossary 38

1. Trial Summary

1.1. Protocol Summary

Trial Title	Revascularisation for Ischaemic Ventricular Dysfunction (**REVIVED-BCIS2**)
Aim	To evaluate the efficacy and safety of percutaneous coronary intervention (PCI) compared to optimal medical therapy (OMT) alone for ischaemic left ventricular dysfunction
Trial Design	Multicentre prospective randomised open controlled trial
Primary Endpoint	All-cause death or hospitalisation due to heart failure
Secondary Endpoints	Quality of life score:<br>Kansas City Cardiomyopathy Questionnaire (KCCQ)<br>EuroQol EQ-5D-5L<br>New York Heart Association (NYHA) Functional Class<br>Left ventricular ejection fraction (LVEF) on echocardiography at 6 months and 1 year<br>Hospitalisation for heart failure<br>All-cause death<br>Cardiovascular death<br>Acute myocardial infarction (MI)<br>Appropriate implantable cardioverter defibrillator (ICD) therapy<br>Unplanned further revascularisation<br>Canadian Cardiovascular Society (CCS) angina class<br>Health resource use<br>Brain natriuretic peptide (BNP or NT-proBNP) level<br>Troponin (T or I) level<br>Major bleeding
Inclusion Criteria	LVEF ≤35%<br>Extensive coronary artery disease (CAD)<br>Viability in at least 4 dysfunctional myocardial segments, that can be revascularised by PCI
Major Exclusion Criteria	Acute MI <4 weeks prior to randomisation (clinical definition)<br>Acutely decompensated heart failure requiring treatment with inotropes/ventilation/mechanical circulatory support <72 hours prior to randomisation<br>Any contraindication to PCI

Sample Size and Enrolment	n=700<br>Start date: 1st June 2013<br>Recruitment start date: 1st September 2013<br>Recruitment end date: 30th April 2020<br>Follow-up end date: 30th April 2022<br>Number of centres: 35-40 (listed on trial website)

1.2. Trial Flowchart

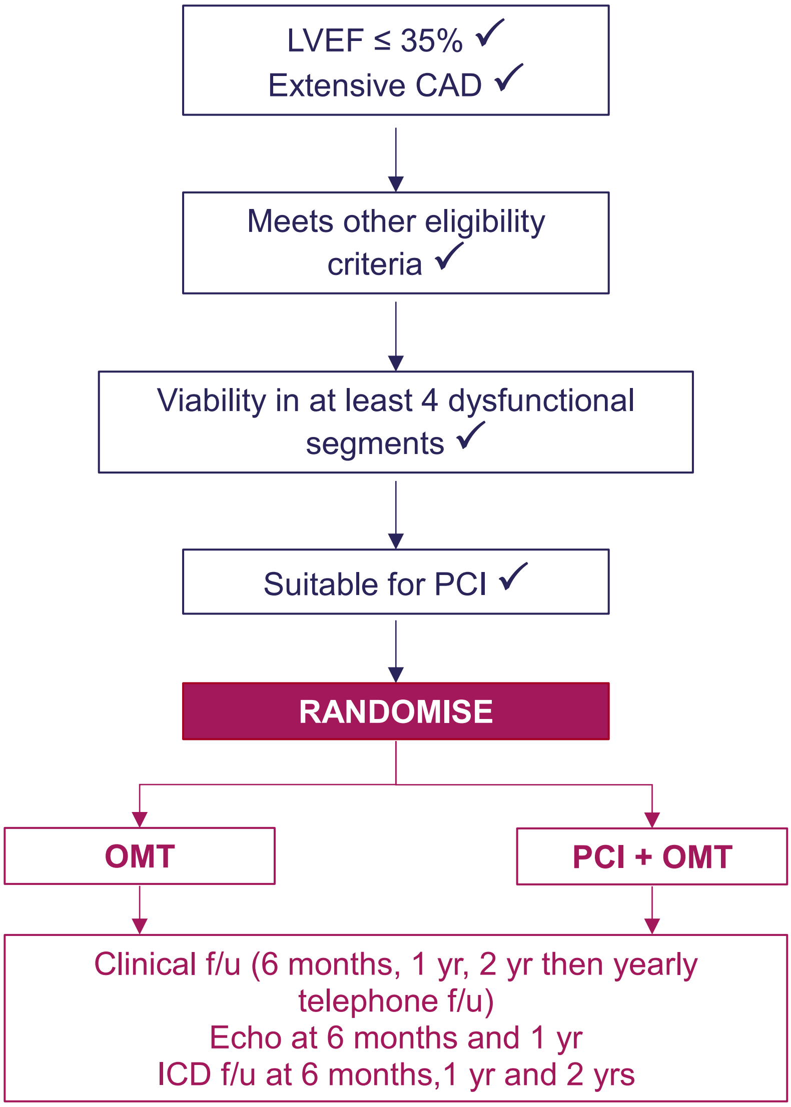

1.3. Trial Organisation

1.3.1. NIHR HTA CET Grant applicants

Prof Divaka Perera, King's College London (Chief Investigator)

Associate Prof Tim Clayton, London School of Hygiene & Tropical Medicine

Prof Simon Redwood, King's College London

Dr Mark De Belder, The James Cook University Hospital, Middlesbrough

Prof Tony Gershlick, Glenfield Hospital, Leicester

Prof Michael Marber, King's College London

Prof Theresa McDonagh, King's College London

Dr Gerry Carr-White, Guy's and St Thomas' Hospital, London

Prof Mark Sculpher, Centre for Health Economics, University of York

1.3.2. Trial Steering Committee (TSC)

Prof Andrew Clark, Chair of Clinical Cardiology, Castle Hill Hospital, Hull (chair)

Mrs Helen Williams, Pharmacist, NHS Southwark Clinical Commissioning Group

Dr Pablo Perel, Epidemiologist, London School of Hygiene & Tropical Medicine

Dr David Walker, Cardiologist, Conquest Hospital, St. Leonards-on-Sea

Prof Rod Stables, Cardiologist, Liverpool Heart and Chest Hospital

Prof Divaka Perera, King's College London

Ms Liz Bestic, Consumer representative

Mrs Paula Young, Consumer representative

1.3.3. Project Management Group (PMG)

Prof Divaka Perera, King's College London

Associate Prof Tim Clayton, London School of Hygiene & Tropical Medicine

Mr Steven Robertson, London School of Hygiene & Tropical Medicine

Mr Richard Evans, London School of Hygiene & Tropical Medicine

Ms Ruth Canter, London School of Hygiene & Tropical Medicine

Mrs Karen Wilson, Guy's and St Thomas' Hospital, London

Mrs Sophie Arnold, Guy's and St Thomas' Hospital, London

Dr Bhavik Modi, Guy's and St Thomas' Hospital, London

Dr Natalia Briceno, Guy's and St Thomas' Hospital, London

Dr Matthew Ryan, Guy's and St Thomas' Hospital, London

1.3.4. Clinical Trials Unit (CTU)

The trial is managed by the UKCRC accredited CTU at London School of Hygiene & Tropical Medicine (Registration ID 44).

1.3.5. Data and Safety Monitoring Committee (DSMC)

Dr Peter Ludman, Consultant Cardiologist, Birmingham (chair)

Dr Suzanna Hardman, Consultant Cardiologist, Whittington Hospital, London

Dr Louise Brown, Senior Statistician, MRC Clinical Trials Unit at University College London

The DSMC is supported by Mr Matt Dodd, Statistician at the London School of Hygiene & Tropical Medicine CTU

1.3.6. Clinical Events Committee (CEC)

Prof Roxy Senior, Professor of Clinical Cardiology, Royal Brompton Hospital, London (chair)

Dr Zaheer Yousef, Consultant Cardiologist, University Hospital of Wales

Dr Rajan Sharma, Consultant Cardiologist, St George's Hospital, London

1.3.7. Medical Therapy Committee

Prof Michael Marber, Professor of Cardiology, King's College London

Prof Aldo Rinaldi, Consultant Cardiologist, St Thomas' Hospital, London

Dr Stam Kapetanakis, Consultant Cardiologist, St Thomas' Hospital, London

Prof Mark Petrie, Consultant Cardiologist, Golden Jubilee Hospital, Glasgow

1.3.8. Recruiting Centres

At each site;

- Heart Failure lead
- PCI lead

  (One of which will be designated as the Principal Investigator and the other as a co-investigator)
- Trial Coordinator

A current list of sites is provided on the trial website http://revived.lshtm.ac.uk/

2. Background

2.1. Epidemiology

In 2002, it was estimated that approximately 900,000 individuals in the UK had a diagnosis of heart failure and at least 5% of all deaths in the country were related to this condition. At that time, one million in-hospital bed-days per year were estimated to be due to heart failure, with an annual cost to the NHS in excess of £625 million. Furthermore, there is evidence of a rising prevalence of heart failure in the population, with the number of associated hospital admissions expected to increase by around 50% in the next 25 years(1). This emerging epidemic is the likely consequence of a progressively aging population and improved survival from acute coronary syndromes, partly due to more efficient and timely revascularisation techniques. The Framingham Heart Study suggests that the most common cause of chronic heart failure is no longer hypertension or valvular heart disease, as it was in previous decades, but rather coronary artery disease(2). Recent meta-analyses of heart failure trials and large registries have shown that coronary disease is the underlying cause of heart failure in 65% of cases(3, 4), although this may have been an underestimation, given that few of these studies mandated systematic exploration of aetiology.

2.2. Hibernating Myocardium

The concept of viable but dysfunctional myocardium emerged approximately three decades ago, when it was observed that patients undergoing coronary artery bypass surgery for chronic stable angina had improvement or normalisation of left ventricular function following revascularisation(5). The energy utilized during myocyte contraction far exceeds the requirement for sustaining viability and, as such, myocardial tissue may survive in a hypocontractile state in the presence of reduced coronary blood flow or decreased coronary flow reserve, known as hibernation(6). Improvement of blood flow by revascularisation of hibernating myocardium can lead to restoration of regional and global left ventricular function and reversal of adverse remodelling(7-9), provided this is achieved before the onset of irreversible cellular and ultrastructural alterations(10). Potentially reversible, dysfunctional myocardium is characterised by preserved cellular integrity and a degree of contractile reserve, whereas scarring and absence of inducible contraction tend to reflect irreversible myocardial damage. Each of these distinguishing features can be used to predict myocardial viability or the likelihood of functional recovery following revascularisation. The parameter most widely used to determine viability is contractile reserve, which is assessed by measuring the augmentation of function of hypocontractile myocardium, in response to inotropic stimulation. The most commonly used agent is Dobutamine (at doses up to 20 μg/kg/min) while the change in regional and global contractility could be imaged by dobutamine stress echocardiography (DSE) or cine-magnetic resonance imaging (MRI). While MRI allows scar imaging as well as assessment of contractile reserve, at present it is contraindicated in patients with implantable cardioverter defibrillators or pacemakers in situ, which can limit its use in a heart failure population.

Despite variation in the sensitivity and specificity of MRI, DSE, positron emission tomography (PET) and Nuclear Medicine techniques, patients found to have viable myocardium (by any modality) have been shown to have a strong survival advantage following revascularisation compared to medical therapy alone. A meta-analysis of more than 3000 patients in 24 observational studies (in which viability was assessed by single photon emission computed tomography (SPECT), PET or DSE) showed an impressive 80% relative reduction (and 12.8% absolute reduction) in mortality with revascularisation compared to medical therapy in patients found to have significant viable myocardium(8). In contrast, no survival benefit was seen in the absence of viability and even a trend to worse outcome with revascularisation.

These data also argue against a strategy of revascularising all patients with heart failure and coronary disease, regardless of viability; mortality following coronary artery bypass graft (CABG) surgery in patients without viability was more than double that observed in those who did have viable myocardium.

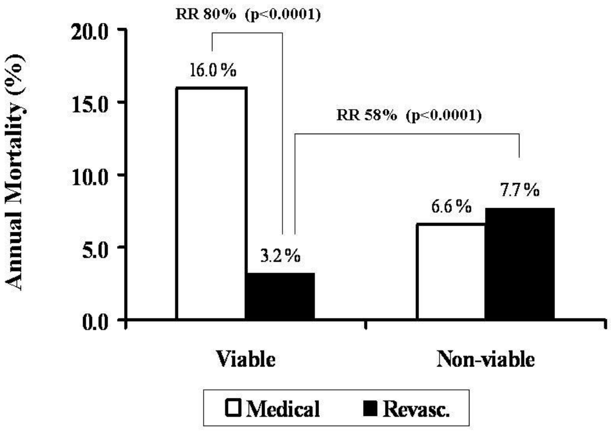

A more recent analysis of 14 non-randomised studies suggests that the findings of the Allman meta-analysis have not changed despite changes in revascularisation techniques and medical therapy(11). It has traditionally been held that completeness of revascularisation (in relation to the angiographic findings) is a major determinant of outcome in ischaemic cardiomyopathy(12); whether regional viability can be used to guide the extent (and hence the mode) of revascularisation in a given patient, remains untested to date.

Notwithstanding the compelling nature of these small studies, there is a lack of consensus on the role of revascularisation in patients with heart failure owing to the absence of adequately powered randomised controlled trials (RCTs) in this field(13-15). Furthermore, there have been major advances in medical therapy for heart failure during the last decade and the incremental benefit of revascularisation in contemporary practice is unknown. REVIVED-BCIS2 will be the largest contemporary randomised comparison of percutaneous revascularisation (with optimal medical therapy (OMT)) versus OMT alone in patients with heart failure and viable myocardium, and is expected to definitively resolve the role of this treatment.

2.3. CABG surgery for ischaemic cardiomyopathy

CABG surgery is considered an appropriate treatment for impaired left ventricle (LV) function in the presence of significant proximal coronary disease, regardless of whether the patient has angina(13-16). These recommendations were based on data from registries and cohort studies that were carried out more than 20 years ago, before the routine use of medical therapies that have been shown to improve survival and symptoms in this group of patients. The Coronary Artery Surgery Study (CASS) registry included 651 (of a total of approximately 20,000) patients who had a left ventricular ejection fraction (LVEF) <50%, 231 of whom received CABG surgery. CABG provided a mortality benefit over medical therapy only in the subgroup of patients with severe LV dysfunction (ejection fraction (EF) <25%), where angina was the predominant symptom, rather than heart failure(17). The Duke registry of 1391 patients with ischaemic cardiomyopathy (EF <40%), treated over a period of 25 years, demonstrated a sustained survival benefit in the group receiving CABG surgery (339 patients) compared to those treated with medical therapy alone(18).

The landmark Surgical Treatment for Ischemic Heart Failure (STICH) trial is the only completed RCT to date that addressed this question(19). This was an international multicentre, open-labelled RCT that enrolled 1212 patients with LV dysfunction (EF <35%) with follow up for an average of 4.7 years. The main hypothesis was that a strategy of coronary artery bypass grafting and OMT compared with OMT alone would reduce the primary outcome of all-cause mortality. The primary outcome was not found to be significantly different between groups (41% OMT versus 36% CABG, p=0.12). There was a trend for a reduction in the secondary outcome of cardiovascular mortality in the CABG treated group, which did not quite make statistical significance (p=0.05). The Surgical Treatment for Ischaemic Heart Failure Extension Study (STICHES) reported longer-term mortality data from the STICH trial; 98% of the study cohort was followed up for a median of 9.8 years, during which time 59% of patients assigned to CABG died versus 66% in the medical therapy group (hazard ratio 0.84; 95% confidence interval, 0.73-0.97; p=0.02)(20). Death from cardiovascular causes and several pre-specified composite secondary endpoints also occurred less often in the CABG group. Patients with more severe coronary artery disease, a left ventricular aneurysm suitable for surgical reconstruction, who were classified as being Hispanic/Latino/non-white or were younger than 60 years had the greatest survival benefit with revascularisation (p values for interaction 0.04, 0.03, 0.02 and 0.18 respectively)[60].

Several considerations should be taken into account when interpreting the above data. Firstly, although the trial at its onset mandated the presence of viability for enrolment, due to slow recruitment this was removed from the protocol; as a result, a patient population with both non-viable and viable myocardium were enrolled. Secondly, on average 2 patients were enrolled per centre per year, reflecting the fact that this was a difficult trial to recruit to, and may indicate selection bias. Importantly, the CABG procedure itself conferred a higher 30-day mortality than with medical therapy, which may have ameliorated any benefit seen with revascularisation, an effect which lasted for more than 2 years. This finding is in keeping with registry data on CABG surgery: perioperative mortality rates in patients with LV dysfunction have been shown to be between 5% and 30%; and the risk increasing with age, comorbidities and degree of LV impairment(21). The relative risk of early death following CABG surgery in patients with severe LV dysfunction is 3- to 4-fold higher than in those with mild dysfunction or preserved systolic function(22-24). However for patients who survive this early mortality hazard, there may be a long-term mortality benefit from CABG. Another consideration is the age of the population enrolled in STICH(ES) and whether this relatively young population are representative of the average heart failure patient. In STICHES, the reduction in mortality was about 25% for those aged <60 years (slightly more than half of all patients) but only 9% in those aged >60 years.

Furthermore, patients with left main coronary stenoses (who represent the extreme end of the spectrum of coronary disease and therefore are at highest risk of cardiovascular events) were excluded from the trial. Finally, the STICH investigators did not systematically exclude patients with non-ischaemic cardiomyopathy with co-existent coronary disease; a minimum coronary disease severity was not mandated and, as a consequence, 40% of the entire cohort had single or 2 vessel disease only. Potential inclusion of non-ischaemic cardiomyopathy patients would be expected to dilute any beneficial effects of revascularisation.

2.4. **PCI for ischaemic cardiomyopathy**

Numerous comparisons have been made between percutaneous coronary intervention (PCI) and CABG surgery for patients with symptomatic coronary disease or evidence of significant reversible ischaemia, but most of the large RCTs excluded patients with impaired left ventricular function (EF <30%)(26-28). Less than 2% of all patients included in the largest and most recent RCT, the Synergy between PCI with Taxus and Cardiac Surgery (SYNTAX) trial, had significant LV impairment (EF <30%) at baseline(29). A meta-analysis of 10 such trials has

found similar 5-year survival following surgery or PCI in the combined cohort, as well as in the subgroup (17% of all patients) who had modest LV dysfunction(30). We recently reported mortality rates of 1.3% and 6% at one and 6 months respectively, following PCI in 301 patients with severely impaired LV function (EF 24%) and severe coronary disease (British Cardiovascular Interventional Society (BCIS-1) Jeopardy Score (JS) of 10/12)(31). Long-term all-cause mortality assessment in this cohort was completed in October 2011, by tracking the database of the Office for National Statistics in the UK. These data provide the best contemporary indication of the utility of PCI in ischaemic cardiomyopathy. All-cause mortality at a median of 51 months (range 28-70) was 33%(32). Notwithstanding the inherent difficulties of carrying out a non-randomised comparison, it is worth noting that mortality in the 600 medically treated patients in STICH was 46% at a median of 56 months (range 12-72), despite having better overall LV function (EF 28%) and a lower coronary disease burden than the contemporaneous BCIS-1 cohort. These results may suggest that PCI may be the preferred mode of revascularisation for patients with ischaemic cardiomyopathy who have suitable coronary anatomy. The ability to carry out surgical ventricular reconstruction has also been traditionally considered an indication for CABG surgery rather than PCI, but Hypothesis 2 of the STICH trial suggests that ventricular restoration does not offer survival or functional benefit over revascularisation alone(33).

There have been a few non-randomised comparisons of the two modalities in patients with poor LV function. In the pre-stent era, observational studies suggested better early outcomes but less complete revascularisation and more mid-term repeat revascularisation procedures following balloon angioplasty than surgery, with similar long-term survival following either treatment(12, 34). The Angina With Extremely Serious Operative Mortality Evaluation (AWESOME) investigators combined the data from randomised and registry cohorts in a pre-specified subgroup analysis and demonstrated equivalent 3-year survival following surgery or bare-metal stent PCI(35). The advent of drug-eluting stents has vastly reduced the incidence of restenosis and has facilitated a greater degree of revascularisation with PCI, which are particularly pertinent factors in the treatment of ischaemic cardiomyopathy(36). A recent observational study has confirmed these theoretical benefits by demonstrating comparable mortality at 15 months following drug-eluting stent PCI or CABG surgery, although there was a greater improvement in New York Heart Association (NYHA) functional class with surgery, possibly due to more complete revascularisation(37). However, these studies were relatively underpowered retrospective analyses that included patients who had significant angina and were not balanced in terms of baseline characteristics or completeness of revascularisation. At present, although conceptually appealing, there is no randomised evidence supporting the use of PCI for patients with ischaemic cardiomyopathy and predominant symptoms of heart failure, rather than angina. There is clearly a need for systematic evaluation of the safety and efficacy of this treatment by a RCT. Furthermore, there have been major advances in medical therapy for heart failure during the last decade and the incremental benefit of revascularisation in contemporary practice is unknown. REVIVED-BCIS2 will be the largest contemporary randomised comparison of percutaneous revascularisation (with OMT) versus OMT alone in patients with heart failure and viable myocardium, and is expected to definitively resolve the role of this treatment.

3. Hypothesis

Compared to OMT alone, PCI improves event-free survival in patients with ischaemic cardiomyopathy and viable myocardium.

4. Endpoints

An independent clinical events committee (CEC), who are blinded to treatment assignment, will centrally adjudicate and validate selected endpoints where validation is necessary.

4.1. Primary Endpoint

All-cause death or hospitalisation due to heart failure. This composite endpoint will be collected over the entire duration of follow-up in the trial.

4.2. Major Secondary Endpoints

LVEF on echocardiography at 6 months and 1 year

Quality of life score:

- Kansas City Cardiomyopathy Questionnaire (KCCQ)
- EuroQol EQ-5D-5L

NYHA Functional Class

4.3. Other Secondary Endpoints

Cardiovascular death
All-cause death
Hospitalisation due to heart failure
Acute myocardial infarction (MI)
Appropriate implantable cardioverter defibrillator (ICD) therapy
Unplanned further revascularisation
Canadian Cardiovascular Society (CCS) angina class
Health resource use
Serial Troponin (T or I) levels
Serial brain natriuretic peptide (BNP or NT-proBNP) levels
Major bleeding

4.4. Endpoint Definitions

Acute Myocardial Infarction	**1. Spontaneous MI (>48 hrs after PCI/CABG)**<br><br>Detection of a rise and/or fall of cardiac biomarkers (preferably Troponin (T or I), with at least one value higher than the 99th percentile upper reference limit (URL)*) AND symptoms consistent with ischaemia OR dynamic electrocardiogram (ECG) changes (including >1mm ST elevation, new Left Bundle Branch Block (LBBB) >1mm ST depression, >3mm T wave inversion).<br><br>**2. Peri-procedural MI (<48 hrs after PCI/CABG)***<br><br>**Following PCI**: Troponin (T or I) >5 times the 99th percentile URL (or 5 times the baseline value if this is higher than the URL) in combination with any of the following: (i) evidence of prolonged ischaemia (>20 min) as demonstrated by prolonged chest pain and/or ischaemic ST changes; (ii) new pathological Q waves; (c) angiographic evidence of a flow limiting complication, such as of loss of patency of a side branch, persistent slow-flow or no-reflow, embolisation; or (d) imaging evidence of new loss of viable myocardium or new regional wall motion abnormality.<br><br>**Following CABG**: Troponin (T or I) >10 times the 99th percentile URL (or 10 times the baseline value if this is higher than the URL) in combination with any of the following: (i) new pathological Q waves; (ii) angiographically documented new graft or new native coronary artery occlusion; or (iii) imaging evidence of new loss of viable myocardium or new regional wall motion abnormality.<br><br>**3. Sudden death**<br><br>Cardiac arrest accompanied by new ST elevation/LBBB on ECG and/or evidence of fresh coronary thrombus at autopsy/angiography.<br><br>** In addition to classifying patients dichotomously, on the basis of the 2012 Universal Definition of MI(38), as having suffered a periprocedural MI or not, baseline and peak Troponin (T or I) levels measured within 24 hours of a procedure will be recorded. This will provide a continuous outcome measure of periprocedural myocardial injury and will also allow subsequent reclassification in the event of further revisions to the Universal Definition during the course of the trial.*

Hospitalisation for heart failure (39, 40)	Hospital admission (lasting >24 hours) for deteriorating symptoms or signs of heart failure, where there is a documented diagnosis of heart failure and the patient receives initiation or intensification of treatment for heart failure. Initiation or intensification of treatment includes at least one of the following: increase in oral diuretic dose or addition of another oral diuretic; intravenous diuretic therapy; intravenous vasoactive therapy (vasodilator, inotrope or vasopressor); mechanical circulatory support (MCS) (including intra-aortic balloon pump (IABP), Impella, extra-corporeal membrane oxygenation (ECMO)); or cardiac transplantation.<br><br>Heart failure during or after the assigned PCI procedure itself is defined as prolongation of the planned admission by at least 24 hours due to acute heart failure requiring initiation or intensification of treatment as defined above. Prolongation of hospital admission in patients who have prophylactic pre-PCI insertion of a mechanical support device (IABP, Impella or ECMO) should not be recorded as having a heart failure hospitalisation UNLESS there are features of heart failure requiring initiation or intensification of treatment as defined above.<br><br>Elective admission for implantation or revision of ICD/cardiac resynchronisation therapy (CRT) devices will NOT constitute an endpoint.
Unplanned revascularisation	**PCI group:** Any unplanned target vessel or non-target vessel revascularisation by PCI or CABG following index PCI, excluding provisional staged PCI (with plan documented at the index procedure).<br><br>**OMT group:** Any revascularisation by PCI or CABG.
Appropriate ICD therapy	At least one ICD shock or episode of anti-tachycardia pacing for documented ventricular tachycardia (VT) or ventricular fibrillation (VF).
Cardiovascular death	All deaths where there is no clinical or post-mortem evidence of a non-cardiovascular aetiology.

<table>
<tr><td>Major Bleeding</td><td>Major bleeding will be defined using the Bleeding Academic Research Consortium (BARC) categories below:

Type 3

Type 3a
• Overt bleeding plus haemoglobin drop of ≥30 to <50g/L (provided haemoglobin drop is related to bleed)
• Any transfusion with overt bleeding

Type 3b
• Overt bleeding plus haemoglobin drop ≥50g/L (provided haemoglobin drop is related to bleed)
• Cardiac tamponade
• Bleeding requiring surgical intervention for control (excluding dental/nasal/skin/haemorrhoid)
• Bleeding requiring intravenous vasoactive drugs

Type 3c
• Intracranial haemorrhage (does not include microbleeds or haemorrhagic transformation; does include intraspinal)
• Subcategories; confirmed by autopsy, imaging or lumbar puncture (LP)
• Intra-ocular bleed compromising vision

Type 4: CABG-related bleeding
• Perioperative intracranial bleeding within 48 hours
• Reoperation following closure of sternotomy for the purpose of controlling bleeding
• Transfusion of ≥5 units of whole blood or packed red blood cells within a 48-hour period
• Chest tube output ≥2L within a 24-hour period
• If a CABG-related bleed is not adjudicated as at least a Type 3 severity event, it will be classified as ‘Not a bleeding event’

Type 5: fatal bleeding

Type 5a
• Probable fatal bleeding: no autopsy or imaging confirmation, but clinically suspicious

Type 5b
• Definite fatal bleeding: overt bleeding or autopsy or imaging confirmation</td></tr>
</table>

5. Safety Reporting

5.1. Definition

Unexpected events that have not been defined as endpoints (section 4) or expected complications of the PCI procedure (listed in PCI definitions, section 13.4) should be reported as either a serious adverse event (SAE) or non-serious adverse event (NSAE) depending on their severity.

5.2. Unexpected Serious Adverse Events

SAEs should be reported to the Clinical Trials Unit (CTU) within 7 days. The report should include an assessment of causality by the Principal Investigator at each site (see section 5.4.2). The Chief Investigator will be responsible for the prompt notification of findings that could adversely affect the health of patients or impact on the conduct of the trial.

5.3. Unexpected Non-Serious Adverse Events

Unexpected NSAEs should be evaluated by the Principal Investigator. This should include an assessment of causality (see section 5.4.2) and intensity (see section 5.4.1) and reports made within 14 days. The CTU will keep detailed records of all unexpected adverse events reported. Reports will be reviewed by the Chief Investigator to consider intensity, causality and expectedness.

5.4. Reporting unexpected adverse events

Investigators will make their reports of all unexpected adverse events, whether serious or not, to the CTU at the London School of Hygiene & Tropical Medicine.

5.4.1. Assessment of intensity

Mild: The patient is aware of the event or symptom, but the event or symptom is easily tolerated.

Moderate: The patient experiences sufficient discomfort to interfere with or reduce his or her usual level of activity.

Severe: Significant impairment of functioning; the patient is unable to carry out usual activities and/or the patient’s life is at risk from the event.

5.4.2. Assessment of causality

Probable: A causal relationship is clinically / biologically highly plausible and there is a plausible time sequence between onset of the adverse event and the PCI procedure / commencement of OMT.

Possible: A causal relationship is clinically / biologically plausible and there is a plausible time sequence between onset of the adverse event and the PCI procedure / commencement of OMT.

Unlikely: A causal relationship is improbable and another documented cause of the adverse event is most plausible.

Unrelated: A causal relationship can definitely be excluded and another documented cause of the adverse event is most plausible.

5.5. Notification

The Sponsor, the Research Ethics Committee (REC) and the Data and Safety Monitoring Committee (DSMC) will be notified by the CTU when reported SAEs have been classified by the Chief Investigator as **both** unexpected and given a causality classification of either Probable or Possible.

6. Trial Population

6.1. Inclusion Criteria

ALL of the following:

1. Poor left ventricular function (EF ≤35%)#
2. Extensive coronary disease*
3. Viability in at least 4 dysfunctional myocardial segments that can be revascularised by PCI

# Biplane/3D echocardiography or MRI can be used to assess the qualifying LVEF. The imaging study should be performed at least 4 weeks after a MI, if there has been a recent clinical diagnosis of a MI.

** In general, patients who do not have bypass grafts will be eligible if they have at least proximal left anterior descending (LAD) disease or at least proximal 2 vessel disease. For patients with patent bypass grafts, or in cases where the extent of coronary artery disease (CAD) is uncertain, the BCIS-1 JS should be calculated. The maximum possible JS score is 12 and a score ≥6 is required to be eligible for REVIVED. N.B. The JS should be based on all coronary disease, not just the vessel subtending viable myocardium.*

6.2. Exclusion Criteria

1. MI <4 weeks prior to randomisation (clinical definition as adjudicated by recruiting centres)
2. Acutely decompensated heart failure requiring inotropic support, invasive or non-invasive ventilation or Mechanical Circulatory Assist therapy <72 hours prior to randomisation
3. Sustained VT/VF or appropriate ICD discharges <72 hours prior to randomisation
4. Valve disease deemed by the local heart team to require imminent intervention
5. Contraindications to PCI
6. Age <18 years
7. Estimated glomerular filtration rate (eGFR) <25 ml/min, unless established on dialysis
8. Women who are pregnant
9. Previously enrolled in REVIVED-BCIS2 or current enrolment in other trial that may affect REVIVED-BCIS2 outcome data
10. Life expectancy <1 year due to non-cardiac pathology

7. Ethical Considerations

7.1. Consent

Only patients that give written consent will be included in the trial. If fully informed consent is not possible, the patient will not be recruited into the trial. The patient should be given sufficient time to consider the trial, recommended to be 24 hours, following which informed consent will be taken. Consent may be taken once all requirements for inclusion have been met.

Staff at site may telephone potential patients with information about the trial before scheduled hospital appointments. If a patient is interested, then the site can post them the information sheet to read prior to their appointment and follow this up with a further telephone call within a reasonable time frame.

A patient may decide to withdraw from the trial at any time without prejudice to their future care.

7.2. Declaration of Helsinki and Good Clinical Practice

The trial will conform to the spirit and the letter of the Declaration of Helsinki, and in accordance with Good Clinical Practice Guidelines.

7.3. Ethical committee review

The National Research Ethics Service Committee London - Westminster have reviewed and approved the trial (REC reference 10/H0802/46). Copies of the letters of approval are to be filed in the trial site files at each centre.

8. Statistical Considerations

8.1. Power Calculation

The predicted occurrence of death or hospitalisation for heart failure at two years is 36% in the OMT group(8, 19, 31, 41, 42). The primary endpoint in REVIVED-BCIS2 will be measured over the entire trial duration, with a minimum follow-up duration of two years, thus increasing the number of events. A trial of 700 (350 in each group) with 300 patients experiencing an event would have over 85% power to detect a hazard ratio of 0.7 (a 30% relative reduction in the hazard) at 5% significance allowing for up to 5% losses by the end of follow-up and increasing recruitment over time. For illustrative purposes this represents a reduction in death or hospitalisation to 27% in the PCI group at two years. The hazard ratio of 0.7 used in the power calculation is pragmatic, while being clinically meaningful and is in line with the magnitude of benefit observed across other treatment modalities in this population.

For the major secondary endpoint, even half this sample size will provide 90% power to detect a minimum difference in EF of 4%, assuming a standard deviation of 11%. The trial is expected to have very good power to detect differences in Quality of Life (one of the major secondary outcomes).

The above predicted event rates take into account the possibility of patients randomised to OMT subsequently undergoing PCI (see below). If a higher event rate is found in the OMT group or patient recruitment rates exceed expectation early in the trial (thus providing a

longer duration of follow-up in a larger proportion of patients), the trial would have greater power to detect a hazard ratio of 0.7, or alternatively, provide over 85% power to detect smaller differences in treatment effect.

Although a smaller treatment effect may be clinically significant, this would have a major impact on sample size, which in turn may affect the feasibility of completing the trial within the proposed timescale and resources.

8.2. **Crossover**

In patients randomly assigned to receive OMT, revascularisation by PCI or CABG during the trial should only be considered in one of the following circumstances:

- Readmission with an acute coronary syndrome (ACS), including ST-elevation myocardial infarction (STEMI) and non-STE events. The diagnosis of ACS will be based on the presence of typical ischaemic symptoms as well as a rise in cardiac biomarker levels or dynamic ST deviation on ECG.
- Deterioration in exertional angina to ≥CCS class 3 level symptoms.
- Resistant ventricular arrhythmias considered to be ischaemic in aetiology.

This trial will be a comparison of strategy, rather than technique, and the projected event rates and hazard ratio allow that OMT patients may undergo subsequent revascularisation. As such, no additional adjustments have been made to the power calculation to account for unplanned revascularisation in the OMT arm.

8.3. **Statistical Analysis**

A detailed statistical analysis plan will be finalised before any analysis of the data by treatment group is undertaken. An unadjusted time-to-event analysis will be performed on the primary endpoint using data across all follow-up, with time to the first event (or censoring) times measured from randomisation. Hazard ratios together with associated confidence intervals will be calculated from the Cox proportional hazards model. The assumptions underlying the Cox model will be assessed. If there is clear non-proportionality, comparisons will also be made in early and later follow-up with cut-points determined based on availability of data prior to unblinding. Cumulative event rates will be calculated and presented using Kaplan-Meier time-to-event curves. As a measure of absolute treatment difference, cumulative event rates will be compared at 2 years. Secondary analyses of each individual component of the primary composite endpoint as well as other secondary time to event outcomes will be analysed using the above methods. Losses to follow-up are expected to be minimal and patients will be included up until the time they experience the event or are censored.

Any categorical outcome measures will be examined at specific time points using risk ratios or risk differences, confidence intervals and chi-square or Fisher's exact tests as appropriate. Continuous variables will be analysed and presented as mean treatment differences, confidence intervals and p values derived from analysis of co-variance models or unpaired t-tests as appropriate (with appropriate transformation if necessary). Analysis of endpoints in the randomised cohort will be by intention-to-treat.

A limited number of subgroups for the primary endpoint will be pre-specified in the analysis plan and are likely to include groups stratified by age, the extent of coronary disease (BCIS-1 JS <12 vs. 12), degree of LV dysfunction (EF <20% vs. ≥20%), diabetes, NYHA class (<3 vs. ≥3) and chronic total occlusion (CTO). In addition, a model will be developed and patients will be categorised according to their baseline risk of the primary outcome and this will be used to examine whether the impact of treatment depends on a person's underlying risk. Since the subgroup analyses are secondary analyses and exploratory in nature, the trial has not been

powered for these. A Cox proportion hazards model incorporating tests of interaction will be used for subgroup analyses.

Other analyses such as sensitivity and per-protocol analyses will be detailed in the statistical analysis plan.

8.4. Interim Analysis

An interim analysis of recruitment and pooled event rates was performed approximately one year after the first patient was recruited to inform the feasibility of completing the trial within the initial projected period. As the number of patients randomised was still relatively small and length of follow-up short, it was felt that the expected number of events at this stage of the trial was too low for meaningful assessment. Recruitment and the pooled event rate will continue to be monitored as the trial progresses.

An independent DSMC has been established and a separate DSMC charter developed which includes details of the meeting schedule and stopping guidelines. The DSMC is expected to meet at least annually.

9. Screening

9.1. Screening population

Patients with LVEF <40% should be screened for eligibility. They may come from the following sources:

- Patients referred to the heart failure team for initiation or optimisation of medical therapy including inpatient referrals, outpatient nurse led heart failure clinics and referrals from district general hospitals.
- Patients referred for viability assessments who are known to have poor resting LV function.
- Patients referred for consideration of CRT or ICD implantation.
- Patients with poor LV function referred for consideration of revascularisation following coronary angiography.
- Patients referred for coronary angiography to establish the aetiology of a dilated cardiomyopathy, who are found to have coronary artery disease.

9.2. Screening log

Full detailed screening logs of all patients with extensive CAD and EF ≤35% considered for the trial will be completed at sites.

The CTU will collect a snapshot of screening outcomes, once a year, from all participating sites. Only patients who complete the screening process (i.e. randomised, declined, met an exclusion criterion) in that three-month period are required to be entered.

10. Assessment of LVEF

10.1. Qualifying ejection fraction

To determine eligibility for the trial, LVEF can be determined by the following modalities:

- Transthoracic echocardiogram (TTE) (Simpson's biplane on 2D or 3D echocardiography)
- The resting stage of a stress echocardiogram
- Cardiac MRI

The qualifying assessment **must** have been carried out less than 1 year before randomisation. Estimation of LVEF and adjudication of eligibility for enrolment in will be done by each participating centre, using locally agreed protocols.

10.2. Baseline echocardiogram

If the qualifying echocardiogram study (TTE or resting images from a stress echo) was performed less than 6 months before randomisation, this study can also be submitted to the core lab to calculate baseline LVEF.

If the qualifying echocardiogram was done more than 6 months before randomisation, or the qualifying LVEF was assessed using MRI, a further transthoracic echocardiogram should be carried out soon after randomisation and this study submitted to the core lab to calculate baseline LVEF.

10.3. Qualifying EF flowchart:

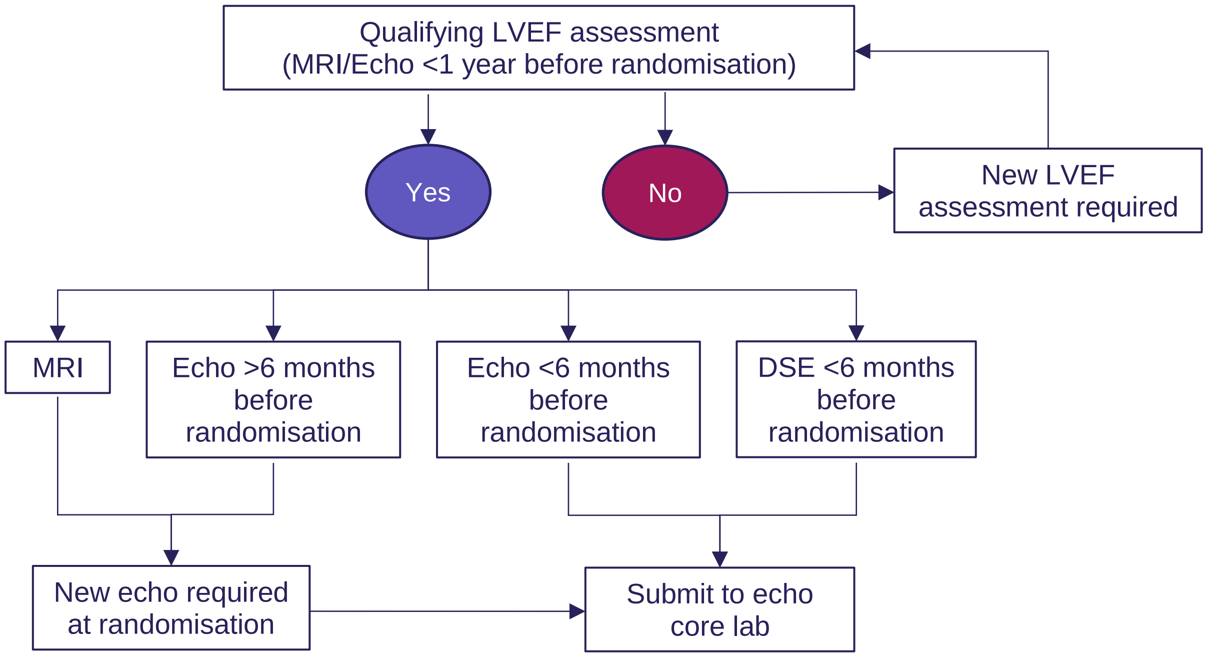

10.4. Patients with recent MI

In the event of a recent myocardial infarction (clinical definition), assessment of qualifying LVEF should be based on a MRI or echocardiogram performed at least 4 weeks after the event.

When a completely new diagnosis is made of heart failure or ischaemic cardiomyopathy, it is recommended that the qualifying echocardiogram or MRI be performed after heart failure medication has been initiated.

10.5. Echo core lab

All trial echocardiograms should be performed in accordance with the minimum standard set out by the British Society of Echocardiography. Baseline, 6-month and 12-month echocardiograms will be anonymised and submitted to an independent echocardiography core laboratory (at Guy's and St Thomas' Hospital, London, UK), which will determine LV volumes and EF using a Simpson's biplane method, for evaluation of the major secondary outcome. The core laboratory will be blinded to treatment assignment as well as to the timing of the studies in relation to randomisation. Core laboratory analysis will also include the degree of mitral regurgitation and segmental wall motion.

In cases where endocardial definition is suboptimal, please consider using intravenous contrast to improve delineation.

Echo core lab analysis will include estimation of end diastolic and end systolic volumes, calculation of LVEF using the Simpson's biplane method and grading segmental wall motion.

10.6. Angiography core lab

Both pre-randomisation and trial procedure coronary angiogram and angioplasty images will be transferred to an angiography core laboratory (at Golden Jubilee National Hospital, Glasgow, UK) via anonymised optical media. Each participant's pre-randomisation BCIS-1 JS and PCI procedural success will be independently validated by the core laboratory. The core laboratory will calculate a number of other scores reflecting the anatomic complexity of coronary disease, the extent of effective revascularisation and the complexity of CTO lesions.

This data will be used to conduct a number of sub-analyses to identify predictors of benefit for the primary and secondary outcomes. The core laboratory will subsequently provide the relevant data to the Sponsor and CTU at the London School of Hygiene & Tropical Medicine for analysis against the data held in the eCRF.

11. Assessment of Viability

Eligibility for the trial will require demonstration of myocardial viability in at least 4 dysfunctional myocardial segments, subtended by diseased coronary arteries that can be treated by PCI.

Regional function at rest will be scored according to the American Heart Association 17 segment-5 grade scoring model (1: normal; 2: mildly hypokinetic; 3: severely hypokinetic; 4: akinetic; 5: dyskinetic)(43). Segments with resting wall motion abnormalities (grade 2-5) will be considered dysfunctional.

Segmental viability can be determined by any imaging modality. The criteria for determining viability will be based on local protocols and as determined by the local imaging specialist,

using all available information; the following are guidelines for defining segmental viability in the REVIVED trial:

- DSE: improvement in contraction by at least one wall motion grade during low-dose Dobutamine stimulation, compared to resting wall motion (improvement by at least 2 grades if aneurysmal or dyskinetic at rest).
- MRI: ≤25% transmural late gadolinium-enhanced (LGE) images. Adjudication of viability in segments with 26-50% transmurality will be at the discretion of the recruiting centres, on the basis of other available information, including of contractile reserve during low-dose Dobutamine stimulation.
- SPECT: tracer activity on the delayed images that is ≥50% of the activity in the segment with maximal activity (in rest-redistribution protocols).
- PET: perfusion – metabolism (FDG) mismatch.

Imaging and intervention specialists at each participating centre will adjudicate segmental viability and the feasibility of revascularising the relevant segments, to determine whether an individual patient will be eligible for randomisation.

12. Randomisation

Potential patients will be reviewed by the Principal Investigator before randomisation with all available tests/notes to confirm eligibility.

Once the eligibility of a patient is confirmed by the trial coordinators and written informed consent obtained, randomisation will be carried out via an online web based system. Randomisation of the treatment assignment will be stratified by centre using randomly permuted blocks of varying size, with 1:1 allocation between the PCI and OMT arms.

There is no time limit from randomisation to PCI. However, it is recommended that index PCI be carried out as close as possible to randomisation to minimise the incidence of major adverse cardiovascular events (MACE) prior to the assigned treatment. Clinical events that occur after randomisation but before planned PCI will be attributed to the assigned treatment on an intention-to-treat basis.

13. Percutaneous Coronary Intervention

13.1. Adjunctive therapy and devices

PCI will be performed according to local protocols. Dual antiplatelet therapy should be given in all cases, with pre-loading, and the post-PCI duration based on the individual's bleeding risk and local/national guidelines. In general, drug-eluting stents are recommended, but in patients who have an indication for long-term formal anticoagulation (e.g. for concurrent atrial fibrillation, LV thrombus or venous thromboembolic disease), the choice of stent type should be based on their suitability for medium-term combined antiplatelet and anticoagulation therapy.

13.2. Completeness of Revascularisation

It is strongly recommended that PCI is considered and, if feasible, attempted on all significant coronary lesions in major proximal coronary vessels (or side branches >2.5mm in diameter) subtending viable myocardium. Lesion significance is defined as >70% diameter stenosis on angiography or for lesions between 50 and 70% diameter stenosis, when accompanied by

demonstrable reversible ischaemia on invasive or non-invasive testing. Planned target lesions will need to be identified by the operator and recorded by the trial coordinator before the procedure.

Patients who meet inclusion criteria and have CTO of coronary arteries subtending viable myocardial segments *should* be considered for REVIVED, provided that the PCI operators predict a high likelihood of successfully reopening these vessels. It is recommended that dedicated CTO operators, in units that have this degree of specialisation, undertake such cases.

The coronary disease burden at baseline and the degree of final revascularisation will be characterised by the BCIS-1 JS and Revascularisation Index (RI) (44), where RI = $(JS_{pre} – JS_{post})/JS_{pre}$.

13.3. Staged PCI

A single stage strategy should be employed where possible. However, provisional staging could be considered in patients with renal dysfunction, complex coronary disease (including chronic total occlusions) or if it is felt during PCI that deferring intervention to one or more vessels is in the patient's best interests (e.g. due to unexpected high contrast volumes or procedural complications during PCI to the first vessel). Staging must be pre-specified at the index procedure.

Urgent revascularisation before the planned 2nd stage procedure will be considered a major endpoint(45).

13.4. PCI Definitions

Target Vessel Success	<30% residual stenosis and Thrombolysis in Myocardial Infarction (TIMI) III flow in target vessel.
Procedural Success	Target vessel success in ALL treated vessels.
Major Procedural Complication	VT/VF requiring defibrillation.<br>Cardiorespiratory arrest requiring assisted ventilation.<br>Prolonged hypotension. (Prolonged hypotension = Mean arterial pressure ≤75 mmHg for >10 min despite fluid resuscitation or requirement of inotropic support / IABP / left ventricular assist device (LVAD) to maintain augmented mean arterial pressure >75 mmHg).
Major Bleeding	≥4 g/dL decrease in haemoglobin relative to baseline (if transfusion required, 1 unit of packed cells / whole blood considered equivalent to 1 g/dL drop in haemoglobin) or intra-cranial haemorrhage.
Minor Bleeding	2-4 g/dL decrease in haemoglobin relative to baseline.

Access complication	Haematoma/limb ischaemia requiring surgical or percutaneous intervention.<br><br>Documented false aneurysm / arterial occlusion.
Acute Kidney Injury (AKI)	An increase in serum creatinine to >150% of the pre-PCI level, within 48 hours of PCI.

14. Optimal Medical Therapy

It is recommended that patients are **initiated** on medical therapy prior to randomisation, however the doses **do not necessarily need to have been optimised** before a patient can be randomised.

In order to ensure that patients in both arms of the trial receive optimal medical and device therapy, there is a nominated heart failure lead at each participating centre who is actively involved in patient selection and monitoring of therapy during the course of the trial. Furthermore a trial Medical Therapy Committee has been established, which will review available evidence and guidelines at least annually and refine recommendations to ensure that drug and device therapy given to all patients in the trial remains optimal and contemporary. Each site is provided with a standard operating procedure for delivering and monitoring OMT, which sets out classes of drugs appropriate for trial patients, including heart failure therapies (such as angiotensin-converting-enzyme (ACE) inhibitor or angiotensin receptor blocker +/- neprilysin inhibitor, betablocker and mineralocorticoid receptor antagonist(13)) and secondary prevention for atherosclerosis (including statin and antiplatelet agent) as well as recommended treatment targets (including lipid profile, HbA1c, resting heart rate). Formal anticoagulation for LV thrombus detected on imaging or as prophylaxis for severe LV dysfunction / dyskinesis is at the discretion of the treating physician. Initiation of the above treatments, dose-titration and relevant monitoring is per local heart failure protocols.

15. ICDs and Cardiac Resynchronisation

ICD implantation is not a requirement for inclusion in REVIVED.

Local guidelines should be followed when deciding on device therapy but the decision to implant (or not implant) a device should be made before randomisation. This plan will be documented in the CRF at baseline.

In evaluating whether an ICD should be implanted, physicians should assume that all patients would be assigned to OMT alone, to minimise the risk of trial outcomes being affected by treatment bias.

16. Data collection and follow-up

16.1. Tests required for eligibility

The following tests are required for identifying and screening patients. These are all standard of care tests and must be performed before patient consent:

- Demographics and medical history
- Coronary angiogram
- Viability assessments
- LVEF assessment – in the case of patients with ACS, this must be done at least four weeks after the ACS
- Creatinine and electrolytes

16.1.1. Time limits for screening tests

**Eligibility criteria**	**Test**	**Time limit**
Extensive coronary disease	Angiogram	Clinically valid
LVEF ≤35%	Resting LVEF assessment	1 year prior to randomisation (at least 4 weeks after ACS)
Viability in at least 4 dysfunctional segments, that can be revascularised by PCI	Viability assessment	Clinically valid

16.2. Trial Checklist

	Tests required for eligibility	Baseline	Pre-PCI as per local protocol ‡	At discharge (up to 16 hours) post-PCI ‡	48 hrs post-PCI ‡	At 6 months post randomisation	At 1 year after randomisation	At 2 years after randomisation	Yearly follow-up	End of trial follow-up
**Clinical assessments (standard of care)**										
Demographics and medical history	X									
Coro Angio	X									
Viability assessment	X									
LVEF Assessment	X*									
Echo		X†								
ICD check		X				X	X	X		
FBC	X			X						
Creatinine‡ & Electrolytes	X			X						
HbA1C		X								
Full Lipid Profile		X								
CK			X	X						
Trop T/I		X	X	X		X	X			
ECG		X		X						
AKI					X					
**Trial specific assessments**										
Echo						X	X			
BNP / NT-proBNP		X				X	X	X		
NYHA/CCS		X				X	X	X		
EQ-5D-5L		X				X	X	X	X	X
KCCQ		X				X	X	X		
Primary Endpoint				X		X	X	X	X	X
Secondary Endpoints						X	X	X	X	
SAEs				X		X	X	X		
Cardiac Medication		X		X		X	X	X		

‡ If PCI is staged, please collect for each stage of the procedure

* In the case of patients with Acute Coronary Syndrome (ACS), must be >4 weeks after ACS

† This echo is only required if there is no available echo within 6 months of randomisation and >4 weeks after ACS

Baseline (up to 6 months prior to randomisation):

- Echo
- Viability assessment report
- ICD check
- HbA1C
- Full lipid profile
- BNP / NT-proBNP
- ECG
- NYHA/CCS
- EuroQol EQ-5D-5L
- Kansas City Cardiomyopathy questionnaire (KCCQ)
- Cardiac medication
- Troponin T/I (OMT arm only)

Pre-PCI as per local protocol (If PCI is staged please collect for each stage of the procedure):

- Troponin T/I or CK

At discharge (or up to 16 hours) post-PCI (If PCI is staged please collect for each stage of the procedure):

- Death
- Creatinine & Electrolytes
- Troponin T/I or CK
- ECG
- Unexpected serious adverse events
- Cardiac medication

48 hours after PCI (If PCI is staged please collect for each stage of the procedure):

- AKI

6 months after randomisation (clinical follow-up):

- Death
- Hospitalisation due to heart failure
- MI
- Major bleeding
- Unplanned further revascularisation
- LVEF on echocardiography
- ICD check
- BNP / NT-proBNP
- NYHA/CCS
- EuroQol EQ-5D-5L
- Kansas City Cardiomyopathy questionnaire (KCCQ)
- Hospitalisation (at St Thomas' only)
- Unexpected serious adverse events
- Cardiac medication
- Troponin T/I

1 year after randomisation (clinical follow-up):

- Death
- Hospitalisation due to heart failure
- MI

- Major bleeding
- Unplanned further revascularisation
- LVEF on echocardiography
- ICD check
- BNP / NT-proBNP
- NYHA/CCS
- EuroQol EQ-5D-5L
- Kansas City Cardiomyopathy questionnaire (KCCQ)
- Hospitalisation (at St Thomas' only)
- Unexpected serious adverse events
- Cardiac medication
- Troponin T/I

2 years after randomisation (clinical follow-up):

- Death
- Hospitalisation due to heart failure
- MI
- Major bleeding
- Unplanned further revascularisation
- ICD check
- BNP / NT-proBNP
- NYHA/CCS
- EuroQol EQ-5D-5L
- Kansas City Cardiomyopathy Questionnaire (KCCQ)
- Hospitalisation (at St Thomas' only)
- Unexpected serious adverse events
- Cardiac medication

Years 3 to 8 after randomisation (telephone follow-up):

- Death
- Hospitalisation due to heart failure
- MI
- Unplanned further revascularisation
- Hospitalisation (at St Thomas' only)
- EuroQol EQ-5D-5L

Final follow-up at end of trial (telephone follow-up):

- Death
- Hospitalisation due to heart failure
- EuroQol EQ-5D-5L

16.3. **Data Handling**

16.3.1. Data Collection

Data will be collected electronically via a web-based case report form (eCRF). In addition, hard copies of ECGs should be maintained at each centre in a physical CRF.

eCRFs should be completed within 2 weeks of each trial milestone (hospital discharge, 6 months etc.), where possible.

Principal Investigators at each site have overall responsibility for the accuracy, completeness and legibility of the data entered onto the eCRF and all associated reports.

16.3.2. Adverse Events

Expected adverse events (see section 4.4 for endpoint definitions) should be reported in the eCRF. An additional SAE form is not required.

Unexpected adverse events (see section 5 for requirements) should be reported on the relevant SAE or NSAE forms and faxed to the CTU within 7 days of notification for SAE and 14 days of notification for NSAE.

16.3.3. Participant ID Log

A list of all patients enrolled into the trial should be maintained by each centre, containing patient identification numbers, full names, dates of birth and dates of enrolment in the trial, which could be used for unambiguous identification of each patient if required. The patient's enrolment in a trial must also be recorded in the patient's medical record and the general practitioner notified accordingly.

16.3.4. Mortality Tracking

In addition to telephone and hospital follow-up, mortality tracking will be carried out via NHS Digital for up to 5 years from enrolment of the last patient.

17. Health Economic Analysis

A formal health economic analysis will be carried out under the leadership of Prof Mark Sculpher, who heads the team for the Economic Evaluation of Health Technology Assessment at the Centre for Health Economics at the University of York, UK.

REVIVED-BCIS2 will provide a vehicle to collect data to support a cost-effectiveness analysis of PCI in heart failure. Data will be collected on NHS resource use including inpatient days in hospital, use of cardiovascular medication and devices and subsequent cardiovascular procedures. These data will be collected via record forms and questionnaires to patients.

In addition, data will be collected on health-related quality of life using the EQ-5D-5L instrument, a generic, preference-based measure. This will be administered at baseline, at 6-month follow-up and at annual intervals subsequently. Resource use will be valued in monetary terms using routine unit cost data relevant to the NHS. These will include NHS Reference Costs, British National Formulary drug prices and the Personal Social Services Research Unit (PSSRU) survey of unit costs.

In terms of analysis, the economic evaluation will consist of a description of resource use, costs and EQ-5D-5L data collected within the trial. A formal cost effectiveness of PCI in this population will be undertaken using a decision analytic framework which is necessary for two main reasons. Firstly, to extrapolate costs and benefits over a longer-term time horizon than that implied by the follow-up period of RCTs. For example, any impact of PCI on mortality will need to be expressed in terms of additional survival duration which requires a model to reflect long term all-cause mortality risks for this patient group. The second reason for using a modelling framework is that it provides a means of synthesising the evidence collected in REVIVED-BCIS2 with any other relevant evidence available in the literature. Most importantly

other RCTs of PCI in heart failure will need to be systematically identified, synthesised with REVIVED-BCIS2 if appropriate and used to assess cost-effectiveness. The structure of the model will be informed by a review of recent modelling studies in the field of cardiovascular disease in general and in heart failure in particular. However, it is anticipated that it will be a cohort model with states representing death and different levels of heart failure symptoms. The modelling approach will also reflect work undertaken by the health economics team in the cardiovascular field using individual patient data from randomised trials(47, 48). The model will be extensively validated to ensure that it can replicate the results of the REVIVED-BCIS2 trial and generates longer-term estimates of survival and costs consistent with available epidemiological evidence in this area.

The cost effectiveness analysis will adhere to the reference case defined by the National Institute for Health and Clinical Excellence for technology appraisal(49). Key features will include the quantification of health benefits in terms of quality-adjusted life years (QALYs) and the use of an NHS cost perspective. Standard decision rules(50) will be used to assess cost effectiveness and extensive sensitivity analysis will be undertaken (probabilistic and deterministic) to assess the implications of uncertainty in the available evidence for cost-effectiveness. Heterogeneity in cost effectiveness between different sub-groups of patients will be assessed using methods consistent with those applied to clinical outcomes.

18. References

1. Peterson S RM, Wolstenholme J. Heart failure supplement. British Heart Foundation Coronary Heart Disease Statistics. 2002.

2. Lloyd-Jones DM, Larson MG, Leip EP, Beiser A, D'Agostino RB, Kannel WB, et al. Lifetime risk for developing congestive heart failure: the Framingham Heart Study. Circulation. 2002;106(24):3068-72.

3. Gheorghiade M, Sopko G, De Luca L, Velazquez EJ, Parker JD, Binkley PF, et al. Navigating the crossroads of coronary artery disease and heart failure. Circulation. 2006;114(11):1202-13.

4. Adams KF, Jr., Fonarow GC, Emerman CL, LeJemtel TH, Costanzo MR, Abraham WT, et al. Characteristics and outcomes of patients hospitalized for heart failure in the United States: rationale, design, and preliminary observations from the first 100,000 cases in the Acute Decompensated Heart Failure National Registry (ADHERE). American heart journal. 2005;149(2):209-16.

5. Rahimtoola SH. Coronary bypass surgery for chronic angina--1981. A perspective. Circulation. 1982;65(2):225-41.

6. Rahimtoola SH. The hibernating myocardium. American heart journal. 1989;117(1):211-21.

7. Bax JJ, Visser FC, Poldermans D, Elhendy A, Cornel JH, Boersma E, et al. Time course of functional recovery of stunned and hibernating segments after surgical revascularization. Circulation. 2001;104(12 Suppl 1):I314-8.

8. Allman KC, Shaw LJ, Hachamovitch R, Udelson JE. Myocardial viability testing and impact of revascularization on prognosis in patients with coronary artery disease and left ventricular dysfunction: a meta-analysis. Journal of the American College of Cardiology. 2002;39(7):1151-8.

9. Carluccio E, Biagioli P, Alunni G, Murrone A, Giombolini C, Ragni T, et al. Patients with hibernating myocardium show altered left ventricular volumes and shape, which revert after revascularization: evidence that dyssynergy might directly induce cardiac remodeling. Journal of the American College of Cardiology. 2006;47(5):969-77.

10. Dispersyn GD, Borgers M, Flameng W. Apoptosis in chronic hibernating myocardium: sleeping to death? Cardiovascular research. 2000;45(3):696-703.

11. Camici PG, Prasad SK, Rimoldi OE. Stunning, hibernation, and assessment of myocardial viability. Circulation. 2008;117(1):103-14.

12. Toda K, Mackenzie K, Mehra MR, DiCorte CJ, Davis JE, McFadden PM, et al. Revascularization in severe ventricular dysfunction (15% < OR = LVEF < OR = 30%): a comparison of bypass grafting and percutaneous intervention. The Annals of thoracic surgery. 2002;74(6):2082-7; discussion 7.

13. Ponikowski P, Voors AA, Anker SD, Bueno H, Cleland JG, Coats AJ, et al. 2016 ESC Guidelines for the diagnosis and treatment of acute and chronic heart failure: The Task Force for the diagnosis and treatment of acute and chronic heart failure of the European Society of Cardiology (ESC)Developed with the special contribution of the Heart Failure Association (HFA) of the ESC. European heart journal. 2016;37(27):2129-200.

14. Windecker S, Kolh P, Alfonso F, Collet JP, Cremer J, Falk V, et al. 2014 ESC/EACTS Guidelines on myocardial revascularization: The Task Force on Myocardial Revascularization of the European Society of Cardiology (ESC) and the European Association for Cardio-Thoracic Surgery (EACTS)Developed with the special contribution of the European Association of Percutaneous Cardiovascular Interventions (EAPCI). European heart journal. 2014;35(37):2541-619.

15. Patel MR, Calhoon JH, Dehmer GJ, Grantham JA, Maddox TM, Maron DJ, et al. ACC/AATS/AHA/ASE/ASNC/SCAI/SCCT/STS 2017 Appropriate Use Criteria for Coronary Revascularization in Patients With Stable Ischemic Heart Disease: A Report of the American College of Cardiology Appropriate Use Criteria Task Force, American Association for Thoracic Surgery, American Heart Association, American Society of Echocardiography, American Society of Nuclear Cardiology, Society for Cardiovascular Angiography and Interventions, Society of Cardiovascular Computed Tomography, and Society of Thoracic Surgeons. Journal of the American College of Cardiology. 2017;69(17):2212-41.

16. Eagle KA, Guyton RA, Davidoff R, Edwards FH, Ewy GA, Gardner TJ, et al. ACC/AHA 2004 guideline update for coronary artery bypass graft surgery: a report of the American College of Cardiology/American Heart Association Task Force on Practice Guidelines (Committee to Update the 1999 Guidelines for Coronary Artery Bypass Graft Surgery). Circulation. 2004;110(14):e340-437.

17. Alderman EL, Fisher LD, Litwin P, Kaiser GC, Myers WO, Maynard C, et al. Results of coronary artery surgery in patients with poor left ventricular function (CASS). Circulation. 1983;68(4):785-95.

18. O'Connor CM, Velazquez EJ, Gardner LH, Smith PK, Newman MF, Landolfo KP, et al. Comparison of coronary artery bypass grafting versus medical therapy on long-term outcome in patients with ischemic cardiomyopathy (a 25-year experience from the Duke Cardiovascular Disease Databank). The American journal of cardiology. 2002;90(2):101-7.

19. Velazquez EJ, Lee KL, Deja MA, Jain A, Sopko G, Marchenko A, et al. Coronary-artery bypass surgery in patients with left ventricular dysfunction. The New England journal of medicine. 2011;364(17):1607-16.

20. Velazquez EJ, Lee KL, Jones RH, Al-Khalidi HR, Hill JA, Panza JA, et al. Coronary-Artery Bypass Surgery in Patients with Ischemic Cardiomyopathy. The New England journal of medicine. 2016;374(16):1511-20.

21. Baker DW, Jones R, Hodges J, Massie BM, Konstam MA, Rose EA. Management of heart failure. III. The role of revascularization in the treatment of patients with moderate or severe left ventricular systolic dysfunction. Jama. 1994;272(19):1528-34.

22. Kennedy JW, Kaiser GC, Fisher LD, Fritz JK, Myers W, Mudd JG, et al. Clinical and angiographic predictors of operative mortality from the collaborative study in coronary artery surgery (CASS). Circulation. 1981;63(4):793-802.

23. Stahle E, Bergstrom R, Edlund B, Frostfeldt G, Lagerquist B, Sjogren I, et al. Influence of left ventricular function on survival after coronary artery bypass grafting. The Annals of thoracic surgery. 1997;64(2):437-44.

24. . !!! INVALID CITATION !!! (19, 20).

25. Perera D, Redwood S, Marber M. CABG in patients with left ventricular dysfunction. The New England journal of medicine. 2011;365(5):468-9; author reply 70-1.

26. Serruys PW, Unger F, Sousa JE, Jatene A, Bonnier HJ, Schonberger JP, et al. Comparison of coronary-artery bypass surgery and stenting for the treatment of multivessel disease. The New England journal of medicine. 2001;344(15):1117-24.

27. Rodriguez A, Bernardi V, Navia J, Baldi J, Grinfeld L, Martinez J, et al. Argentine Randomized Study: Coronary Angioplasty with Stenting versus Coronary Bypass Surgery in patients with Multiple-Vessel Disease (ERACI II): 30-day and one-year follow-up results. ERACI II Investigators. Journal of the American College of Cardiology. 2001;37(1):51-8.

28. Hueb W, Soares PR, Gersh BJ, Cesar LA, Luz PL, Puig LB, et al. The medicine, angioplasty, or surgery study (MASS-II): a randomized, controlled clinical trial of three therapeutic strategies for multivessel coronary artery disease: one-year results. Journal of the American College of Cardiology. 2004;43(10):1743-51.

29. Serruys PW, Morice MC, Kappetein AP, Colombo A, Holmes DR, Mack MJ, et al. Percutaneous coronary intervention versus coronary-artery bypass grafting for severe coronary artery disease. The New England journal of medicine. 2009;360(10):961-72.

30. Hlatky MA, Boothroyd DB, Bravata DM, Boersma E, Booth J, Brooks MM, et al. Coronary artery bypass surgery compared with percutaneous coronary interventions for multivessel disease: a collaborative analysis of individual patient data from ten randomised trials. Lancet. 2009;373(9670):1190-7.

31. Perera D, Stables R, Thomas M, Booth J, Pitt M, Blackman D, et al. Elective intra-aortic balloon counterpulsation during high-risk percutaneous coronary intervention: a randomized controlled trial. Jama. 2010;304(8):867-74.

32. Perera D, Stables R, Clayton T, De Silva K, Lumley M, Clack L, et al. Long-term mortality data from the balloon pump-assisted coronary intervention study (BCIS-1): a randomized, controlled trial of elective balloon counterpulsation during high-risk percutaneous coronary intervention. Circulation. 2013;127(2):207-12.

33. Jones RH, Velazquez EJ, Michler RE, Sopko G, Oh JK, O'Connor CM, et al. Coronary bypass surgery with or without surgical ventricular reconstruction. The New England journal of medicine. 2009;360(17):1705-17.

34. O'Keefe JH, Jr., Allan JJ, McCallister BD, McConahay DR, Vacek JL, Piehler JM, et al. Angioplasty versus bypass surgery for multivessel coronary artery disease with left ventricular ejection fraction < or = 40%. The American journal of cardiology. 1993;71(11):897-901.

35. Sedlis SP, Ramanathan KB, Morrison DA, Sethi G, Sacks J, Henderson W, et al. Outcome of percutaneous coronary intervention versus coronary bypass grafting for patients with low left ventricular ejection fractions, unstable angina pectoris, and risk factors for adverse outcomes with bypass (the

AWESOME Randomized Trial and Registry). The American journal of cardiology. 2004;94(1):118-20.

36. Gioia G, Matthai W, Benassi A, Rana H, Levite HA, Ewing LG. Improved survival with drug-eluting stent implantation in comparison with bare metal stent in patients with severe left ventricular dysfunction. Catheterization and cardiovascular interventions : official journal of the Society for Cardiac Angiography & Interventions. 2006;68(3):392-8.

37. Gioia G, Matthai W, Gillin K, Dralle J, Benassi A, Gioia MF, et al. Revascularization in severe left ventricular dysfunction: outcome comparison of drug-eluting stent implantation versus coronary artery by-pass grafting. Catheterization and cardiovascular interventions : official journal of the Society for Cardiac Angiography & Interventions. 2007;70(1):26-33.

38. Thygesen K, Alpert JS, Jaffe AS, Simoons ML, Chaitman BR, White HD, et al. Third universal definition of myocardial infarction. European heart journal. 2012;33(20):2551-67.

39. Hicks KA, Tcheng JE, Bozkurt B, Chaitman BR, Cutlip DE, Farb A, et al. 2014 ACC/AHA Key Data Elements and Definitions for Cardiovascular Endpoint Events in Clinical Trials: A Report of the American College of Cardiology/American Heart Association Task Force on Clinical Data Standards (Writing Committee to Develop Cardiovascular Endpoints Data Standards). Journal of the American College of Cardiology. 2015;66(4):403-69.

40. Zannad F, Stough WG, Pitt B, Cleland JG, Adams KF, Geller NL, et al. Heart failure as an endpoint in heart failure and non-heart failure cardiovascular clinical trials: the need for a consensus definition. European heart journal. 2008;29(3):413-21.

41. Pfeffer MA, Swedberg K, Granger CB, Held P, McMurray JJ, Michelson EL, et al. Effects of candesartan on mortality and morbidity in patients with chronic heart failure: the CHARM-Overall programme. Lancet. 2003;362(9386):759-66.

42. Maggioni AP, Dahlstrom U, Filippatos G, Chioncel O, Crespo Leiro M, Drozdz J, et al. EURObservational Research Programme: regional differences and 1-year follow-up results of the Heart Failure Pilot Survey (ESC-HF Pilot). European journal of heart failure. 2013;15(7):808-17.

43. Afridi I, Kleiman NS, Raizner AE, Zoghbi WA. Dobutamine echocardiography in myocardial hibernation. Optimal dose and accuracy in predicting recovery of ventricular function after coronary angioplasty. Circulation. 1995;91(3):663-70.

44. De Silva K, Morton G, Sicard P, Chong E, Indermuehle A, Clapp B, et al. Prognostic utility of BCIS myocardial jeopardy score for classification of coronary disease burden and completeness of revascularization. The American journal of cardiology. 2013;111(2):172-7.

45. Vranckx P, Cutlip DE, Mehran R, Kint PP, Silber S, Windecker S, et al. Myocardial infarction adjudication in contemporary all-comer stent trials: balancing sensitivity and specificity. Addendum to the historical MI definitions used in stent studies. EuroIntervention : journal of EuroPCR in collaboration with the Working Group on Interventional Cardiology of the European Society of Cardiology. 2010;5(7):871-4.

46. Hunt SA, Abraham WT, Chin MH, Feldman AM, Francis GS, Ganiats TG, et al. 2009 Focused update incorporated into the ACC/AHA 2005 Guidelines for the Diagnosis and Management of Heart Failure in Adults A Report of the American College of Cardiology Foundation/American Heart Association Task Force on Practice Guidelines Developed in Collaboration With the International Society for Heart and Lung Transplantation. Journal of the American College of Cardiology. 2009;53(15):e1-e90.

47. Briggs A, Mihaylova B, Sculpher M, Hall A, Wolstenholme J, Simoons M, et al. Cost effectiveness of perindopril in reducing cardiovascular events in patients with stable coronary artery disease using data from the EUROPA study. Heart. 2007;93(9):1081-6.

48. Henriksson M, Epstein DM, Palmer SJ, Sculpher MJ, Clayton TC, Pocock SJ, et al. The cost-effectiveness of an early interventional strategy in non-ST-elevation acute coronary syndrome based on the RITA 3 trial. Heart. 2008;94(6):717-23.

49. NICE. Guide to the methods of technology approaisal. National Institute for Hearth and Clinical Excellence. 2008.

50. Drummond MF SM TG, O'Brien BJ, Stoddart GL. Methods for the economic evaluation of health care programmes. 3rd ed: Oxford: Oxford University Press; 2005.

Appendix 1: Glossary

Angiotensin-Converting-Enzyme (ACE) Inhibitor**:** A drug used for the treatment of high blood pressure and sometimes heart failure.

Acute Coronary Syndrome (ACS): This refers to a group of symptoms caused by obstructed coronary arteries. The symptoms include 'crushing chest pains', nausea and sweating. These symptoms usually occur as part of a heart attack.

Activating Clotting Time (ACT)**:** This is a coagulation test, taken after high-dose heparin has been given (i.e. during an angioplasty).

Adenosine**:** A short acting drug used to slow down the heart, often in order to determine a fast rhythm.

Akinetic: This refers to the heart muscles inability to move.

Aldosterone Antagonist: A diuretic used in the management of heart failure (e.g. Spironalactone).

American Heart Association (AHA) 17 segment: This refers to the 17 angles/pictures of the heart that will be captured in the echocardiogram (see definition) 5 Grade Scoring Model- This will be used to grade the severity of impaired movement to the heart muscle wall in each of the 17 angles.

Angiogram Procedure: where a small tube is inserted into the groin or wrist and is passed to the heart. Pictures are then taken of the heart arteries by X-ray to show any narrowing's.

Arrhythmia/Dysrhythmia: An abnormal heart rate caused by abnormal electrical activity- it may be too fast, too slow, regular or irregular.

Atherectomy (rotational): Minimally invasive surgery to remove atherosclerosis from a blood vessel.

Atherosclerosis: An accumulation of fatty materials causing the arterial vessel wall to thicken and contributing to the blockage of blood vessels.

Atrial Fibrillation (AF): A common irregular heartbeat caused by the top chambers in the heart (the atriums) quivering (fibrillating). This rhythm is often the cause of 'palpitations'.

Beta Blocker: A group of drugs that are often used to treat high blood pressure, irregular heart rates and/or heart failure. They act to lower blood pressure and slow the heart rate.

Biphasic Response: Two separate responses that are separated in time.

Biventricular pacemaker: A treatment for heart failure using a pacemaker or ICD to stimulate the right and left side of the heart causing the lower chambers of the heart (ventricles) to beat at the same time.

Brain Natriuretic Peptide (BNP): This is a measure of amino acids (proteins) in the blood that are released in patients with heart failure.

British Cardiovascular Interventional Society (BCIS-1) Jeopardy Score (JS): A scoring system that has been developed to predict procedural risk during PCI.

Cardiac Aneurysm: This refers to a bulging or pocketing on the wall of the inside of the heart, often the left ventricle. This often occurs slowly over a long period of time or as a result of a heart attack (not the same as a vessel aneurysm).

Cardiac Re-Synchronisation Therapy Defibrillator (CRT-D): A device used in patients with heart failure that helps to enhance the blood pumped out with each time the heart beats.

Cardiomyopathy: Heart muscle disease, a measurable deterioration of the myocardium.

Cellular integrity: When the cells in the myocardium are essentially still working, that they have maintained their viability.

Contractile Reserve: This is the ability of the myocardium to increase its contractibility when under 'stress' (i.e. during physical activity or a DSE - see stress echo definition).

Coronary Artery Bypass Graft (CABG) Surgery: To improve the blood flow to the heart. Arteries or vein from elsewhere in the body are grafted to the coronary arteries to bypass the narrowings and improve the blood supply to the heart muscle.

Coronary Artery Disease (CAD): A disease that results in the accumulation of fatty material/plaques forming on the artery vessel wall and restricting the blood flow through the vessel.

Creatinine Kinase (CK): A blood test that measures the presence of cardiac enzymes. These act as markers that can assist in the diagnosis of a heart attack.

Dobutamine: A specific inotropic drug that increases blood pressure by enhancing cardiac muscle contractility. (LD - Low Dose, HD - High Dose).

Dobutamine Stress Echocardiogram' (DSE): See 'Stress Echocardiogram'.

Dyskinetic: This refers to difficulty or abnormality in the movement of the heart muscle (could include slight movement/twitches).

Electrocardiogram (ECG): A test that records the electric activity of your heart. (ST elevation/depression, T wave, QRS complex - these terms represent aspects of an ECG reading).

Estimated Glomerular Filtration Rate (eGFR): This is a test to see how well the kidneys are working. It estimates how much blood is filtered by the kidneys over a given period of time.

HbA1c (Glycated Haemoglobin): This is a form of haemoglobin (see definition) that is used to measure the average level of glucose in the blood over a period of time.

Hibernating Myocardium: A segment of the myocardium where the contraction is affected due to tissue ischemia. Significantly it is potentially reversible through revascularisation. Segments that do have this potential are referred to as 'viable'.

Hypo contractility: This refers to the reduced ability of the heart/myocardium to beat.

Hypokinetic: This refers to reduced movement in the heart muscle.

Implantable Cardioverter Defibrillator (ICD): An ICD is made up of a battery and a small computer. All of the components of the ICD are sealed inside a metal can about the size of a small pager. Additionally, an ICD monitors your heart's rhythm and can deliver therapy such as small electrical impulses and/or shocks through the lead system depending on the need of your heart. If a fast heart rhythm is detected, these small electrical impulses and/or shocks can slow down your heart. An ICD is placed under the skin in the upper chest area during an operation.

Intra-aortic Balloon Pump (IABP): A mechanical device that supports the heart and helps to increase the oxygen supply to the heart muscle and the amount of blood the heart pumps out with each beat.

Left Ventricular Assist Device (LVAD): Mechanical circulatory device that either partially or fully replaces the function of a failing heart.

Left Ventricular Ejection Fraction (LVEF): Often given as a percentage, it is the volumetric fraction of blood pumped out of the left ventricle in the heart with each heartbeat.

Magnetic Imaging Resonance (MRI): A medical imaging technique used in radiology to visualise internal structures in the body. LGE - Late gadolinium-enhanced images is a more advanced MRI, 'Cine Data' or 'Cine MRI' is a four dimensional image taken using MRI.

Magnetic Resonance Perfusion Scan (MRP): A brain scan sometimes performed following carotid endarterectomy surgery.

Major Adverse Cardiovascular Event (MACE): This comprises of a non-fatal heart attack, stroke or a cardiovascular death.

Mitral Valve Regurgitation (MR): The leaking of the mitral valve of the heart, causing blood to flow in the reverse direction.

Myocardium: The middle of the three layers forming the wall of the heart. The cardiac muscle.

Myocardial Infarction (MI) or 'Heart attack': An interruption of blood supply caused by a blockage in the blood vessels to the heart leading to cell or tissue death (infarction).

Myocyte / Myogenic Contraction: This is a contraction of the heart initiated by the cells in the myocardium.

Myocardial Remodelling: This refers to the changes in shape, size and structure to the myocardium surrounding the ventricles. This often happens as a result of a heart attack (global/regional refer to the area of myocardium that has been remodelled and cellular/ultrastructural refers to the extent of remodelling).

New York Heart Association (NYHA): A simple way of classifying the extent of heart failure using physical activity, chest pain and breathless as a measure.

Optimal Medical Therapy (OMT): This includes the best medication (tablets) that are currently available for heart failure, at doses that are individually tailored. This strategy

often also involves insertion of a special type of pacemaker (called a biventricular pacemaker, which may also function as an ICD).

Percutaneous Coronary Intervention (PCI): This procedure is used to treat the narrowed coronary arteries of the heart. A small tube is inserted in the groin or wrist and advanced to the heart. Small balloons and stents are used to open up the narrowings and improve blood flow to the heart muscle. This is sometime also known as Coronary Angioplasty.

Permanent Pace Maker (PPM): A medical device where electrodes are in contact with the heart muscle wall and send electrical impulses that cause contractions to regulate the beating of the heart.

Positron Emission Tomography (PET): An imaging technique that produces 3D images of functional processes in the body.

Proximal/Mid/Distal: These terms refer to the location within a coronary vessel - written in order from the top of the vessel (nearest the aorta) down toward the apex.

Regional Wall Motion (RWM): This refers to an abnormality in the movement of a region of the heart muscle. Scoring will be done using the wall motion scoring index.

Revascularisation: 'To restore blood supply'. This refers to a PCI or CABG.

Single Photon Emission Computed Tomography (SPECT): A type of nuclear imaging that shows how blood flows to tissues and organs.

Stress Echocardiogram (SE): A test that uses sound waves to visualise the beating of the heart when responding to 'stress' i.e. physical activity. Physical activity can be simulated using a drug called Dobutamine (see definition). This is sometimes referred to as a 'Dobutamine Stress Echocardiogram' (DSE).

Trans Thoracic Echocardiogram (TTE): A test that uses sound waves to visualise the beating of the heart using a non-invasive technique; a probe is placed on the chest and can pick up the sound waves through the chest wall.

Ventricular Fibrillation (VF): The heart is not beating effectively as the ventricles instead of contracting in a coordinated fashion are instead quivering (fibrillating). This rhythm is not compatible with life.

Ventricular Tachycardia (VT): A heart rhythm where the ventricles in the heart are beating very fast.

Wall Motion Score Index (WMSI): A score measured following an echocardiogram (see definition) used to assess the movement of the left ventricle. It will be the average of each score taken using the AHA grading scale from 17 views of the heart.

STATISTICAL ANALYSIS PLAN

Study Title: Revascularisation for ischaemic ventricular dysfunction (REVIVED-BCIS2)

Study Registration Number: ISRCTN45979711 / NCT01920048

Statistical Analysis Plan Author: Joanna Dobson, Tim Clayton and Pedro Saramago Goncalves

Statistical Analysis Plan Version: 1.1 dated 4th May 2022

FINAL VERSION APPROVED BY:

Name	Signature	Date
Divaka Perera<br>Chief Investigator		May 6, 2022
Andrew Clark<br>Chair of Trial Steering Committee		May 6, 2022
Tim Clayton<br>Senior Statistician	TC Clayton<br>T C Clayton (May 6, 2022 13:13 GMT+1)	May 6, 2022
Pedro Saramago Goncalves<br>Senior Research Fellow,<br>Centre for Health Economics, York		May 6, 2022

**REVISION OF FINAL VERSION CHRONOLOGY:**			
**VERSION NUMBER**	**EFFECTIVE DATE**	**REASON FOR CHANGE**	**CHANGE APPROVED BY (NAME)**
1.1	4th May 2022	• Updated the Analysis of health economic outcomes section	Tim Clayton

CONTENTS

1 INTRODUCTION....4

2 STUDY SYNOPSIS....4

3 STUDY OBJECTIVES....5

3.1 Primary Objective....5

3.2 Primary Endpoint....5

3.3 Major Secondary Endpoints....5

3.4 Other Secondary Endpoints....5

4 STUDY DESIGN....6

4.1 Overall General Design and Plan....6

4.2 Sample Size....6

4.3 Randomisation and Blinding....6

4.4 Study Assessments....7

5 STUDY POPULATIONS....7

5.1 Selection of Trial Population....7

5.2 Definition of Populations for Analysis....8

5.3 Definition of Subgroup Populations in Different Analyses....8

6 STATISTICAL ANALYSIS....10

6.1 General....10

6.2 Pooling of Sites....10

6.3 Interim Analysis....10

6.4 Time Points for Analysis....10

6.5 Methods for Handling Withdrawals and Missing Data and Other Issues....10

6.5.1 Withdrawals....10

6.5.2 Missing Data....10

6.5.3 Data Transformations....11

6.5.4 Multiplicity....11

6.5.5 Covariate Adjustment....11

6.6 Statistical Analytical Issues....11

7 EVALUATION OF DEMOGRAPHICS, OTHER BASELINE CHARACTERISTICS AND PCI TREATMENT....11

7.1 General....11

7.2 Demographics....12

7.3 Baseline Physical Examination....12

7.4 Screening Medical History....12

7.5 Medications....12

7.6 PCI Treatment 13

8 EVALUATION OF TREATMENT COMPLIANCE 13
8.1 Compliance with Study Intervention 13

9 EVALUATION OF EFFICACY PARAMETERS 13
9.1 Analysis of Primary Outcome Variable: All-cause Death or Hospitalisation due to Heart Failure 13
9.2 Analysis of Major Secondary Endpoints 14
9.2.1 LVEF on Echocardiography at 6 Months and 1 Year 14
9.2.2 Quality of Life Scores 16
9.2.3 NYHA Functional Class at 6 Months, 1 year and 2 years 16
9.3 Analysis of Other Secondary Endpoints 16
9.4 Repeat Hospitalisation due to Heart Failure and Death 18
9.5 Win Ratio for Death, Repeat Hospitalisation due to Heart Failure and KCCQ .. 18
9.6 Other Planned Analyses 19
9.6.1 Impact of COVID-19 19
9.6.2 Implantation of Devices during Follow-up 19
9.6.3 Echocardiography Data Core Laboratory 19
9.6.4 Acute Myocardial Infarction 20

10 EVALUATION OF SAFETY PARAMETERS 20
10.1 Adverse Events 20

11 ANALYSIS OF HEALTH ECONOMIC OUTCOMES 20
11.1 General 20
11.2 Within-trial Analysis Outline 21
11.1 Analysis Methods 21
11.1.1 Health Resource Consumption and Costs 21
11.1.2 Health Benefits 22
11.1.3 Missing Data 22
11.1.4 Sensitivity Analysis 23
11.2 The Economic Analysis Data Validation 23

12 REFERENCES 23

APPENDIX 1: STUDY FLOW CHART 25

APPENDIX 2: SUMMARY PROTOCOL 26

APPENDIX 3: TRIALS PROCEDURES 28

1 Introduction

This Statistical Analysis Plan (SAP) describes the planned analysis and reporting of the clinical trial entitled 'Revascularisation for Ischaemic Ventricular Dysfunction' (REVIVED-BCIS2). This is a multicentre prospective randomised open controlled trial comparing percutaneous revascularisation (with optimal medical therapy) versus optimal medical therapy alone in patients with heart failure and viable myocardium.

The following documents were reviewed in preparation of this SAP:

- REVIVED-BCIS2 trial protocol version 8.3, issued 3rd August 2021
- Case Report Form (CRF), version 6, issued 1st February 2019
- London School of Hygiene and Tropical Medicine (LSHTM) Standard Operating Procedure (SOP) for statistics, issued 16th April 2020
- LSHTM Statistical Analysis Plan template, issued 16th April 2020

The reader of this SAP is encouraged to also read the REVIVED-BCIS2 protocol for details on the conduct of this study.

2 Study Synopsis

Ischaemic cardiomyopathy is the most common cause of heart failure and is associated with significant mortality and morbidity. Surgical revascularisation has been shown to improve long-term outcomes in some patients, but surgery itself carries a major early hazard. Percutaneous coronary intervention (PCI) may allow a better balance between risk and benefit.

REVIVED-BCIS2 is a prospective, multi-centre, open-label, randomised controlled trial, funded by the National Institute for Health Research in the United Kingdom. Patients with ischaemic left ventricular dysfunction will be randomised 1:1 to undergo PCI or receive optimal medical therapy (OMT) alone. Follow-up will be for at least 2 years from randomisation. The primary outcome is all-cause mortality or hospitalisation due to heart failure.

REVIVED-BCIS2 will provide the first randomised data on the efficacy and safety of PCI in ischaemic cardiomyopathy and has the potential to inform guidelines pertaining to both revascularisation and heart failure.

3 Study Objectives

3.1 Primary Objective

To determine whether, compared to OMT, PCI improves event free survival in patients with ischaemic cardiomyopathy and viable myocardium.

3.2 Primary Endpoint

All-cause death or hospitalisation due to heart failure. This composite endpoint will be collected over the entire duration of follow-up in the trial when the last patient randomised has reached 2 years of follow-up post randomisation.

3.3 Major Secondary Endpoints

- Left Ventricular Ejection Fraction (LVEF) on echocardiography at 6 months and 1 year
- Quality of life score:
  - Kansas City Cardiomyopathy questionnaire (KCCQ) up to 2 years
  - EuroQol EQ-5D-5L at 6 months and then yearly to the end of follow-up.
- New York Heart Association Functional (NYHA) Class up to 2 years

3.4 Other Secondary Endpoints

- Cardiovascular death over the entire duration of follow-up
- All-cause death over the entire duration of follow-up
- Hospitalisation due to heart failure over the entire duration of follow-up
- Acute myocardial infarction (MI) over the entire duration of follow-up (until last scheduled annual follow-up visit)
- Appropriate implantable cardioverter defibrillator (ICD) therapy to 2 years
- Unplanned further revascularisation over the entire duration of follow-up (until last scheduled annual follow-up visit)
- Canadian Cardiovascular Society (CCS) up to 2 years
- NHS resource use
- Brain natriuretic peptide (BNP or NT-Pro BNP) up to 2 years
- Major bleeding up to 2 years

4 Study Design

4.1 Overall General Design and Plan

A prospective open multi-centre randomised controlled trial in patients with ischaemic left ventricular dysfunction. Patients will be randomised 1:1 to undergo PCI (with OMT) or receive optimal medical therapy (OMT) (Appendix 1 and Appendix 2).

4.2 Sample Size

The predicted occurrence of death or hospitalisation for heart failure at 2 years is 36% in the OMT group. The primary endpoint in REVIVED-BCIS2 will be measured over the entire trial duration, with a minimum follow-up duration of 2 years, thus increasing the number of events. A trial of 700 (350 in each group), with 300 patients experiencing primary outcome events, would have over 85% power to detect a hazard ratio of 0.7 (a 30% relative reduction in the hazard) at 5% significance allowing for up to 5% losses by the end of follow-up and increasing recruitment over time. For illustrative purposes this represents a reduction to 27% of patients with an event in the PCI group at two years.

The hazard ratio of 0.7 is considered clinically meaningful and in line with the magnitude of benefit observed across other treatment modalities in this population. For the major secondary endpoint of LVEF, even one-half of this sample size will provide 90% power to detect a minimum difference in LVEF of 4%, assuming a standard deviation of 11%.

4.3 Randomisation and Blinding

Randomisation of the treatment assignment will be stratified by centre using randomly permuted blocks of varying size, with 1:1 allocation between the PCI and OMT alone arms. This will be done on a secure web-based service through the Clinical Trials Unit at LSHTM. It is recommended that index PCI be carried out as close as possible to randomisation to minimise the incidence of major adverse cardiovascular events prior to the assigned treatment.

Due to the nature of the intervention being assessed (PCI versus medical therapy) this is an open trial and patients / investigators will not be blinded to treatment assignment. However, independent personnel who are blinded to treatment assignment will centrally adjudicate all major endpoints.

4.4 Study Assessments

Study assessments are detailed in Appendix 3. Patients will be followed up at discharge, 6 months then annually. In addition patients will undergo a final follow-up for death, hospitalisation due to heart failure and to record EQ-5D-5L before the end of follow-up of the trial.

5 Study Populations

5.1 Selection of Trial Population

Inclusion criteria (patients are eligible for the trial if they have **all** of the following)**:**

(1) Poor left ventricular function (LVEF≤35%)

(2) Extensive coronary disease (British Cardiovascular Intervention Society Jeopardy Score (BCIS-JS) ≥6)

(3) Viable myocardium in at least 4 dysfunctional segments that can be revascularised by PCI.

Exclusion criteria:

(1) Acute myocardial infarction < 4 weeks prior to randomisation (clinical definition)

(2) Decompensated heart failure requiring inotropic support, invasive or non-invasive ventilation or IABP/left ventricular assist device (LVAD) therapy <72 hours prior to randomisation

(3) Sustained VT/VF or appropriate ICD discharges <72 hours prior to randomisation

(4) Valve disease requiring intervention

(5) Contra-indications to PCI

(6) Age <18 years

(7) eGFR < 25 ml/min, unless established on dialysis

(8) Women who are pregnant

(9) Previously enrolled in REVIVED-BCIS2 or current enrolment in other study that may affect REVIVED-BCIS2 outcome data

(10) Life expectancy < 1 year due to non-cardiac pathology

5.2 Definition of Populations for Analysis

All patients who provide informed consent will be accounted for in this study. A consort flow diagram will be produced to describe the passage of patients through the trial from enrolment, randomisation, treatment, follow-up and analysis. Study withdrawals and major protocol violations will also be indicated.

The primary outcome analysis will be conducted on all randomised patients on an intention-to-treat (ITT) basis, according to treatment groups to which they were randomised, irrespective of whether they undergo their randomised treatment.

The analyses of the secondary outcomes will also be performed on the same ITT population as the primary outcome.

REVIVED is a randomised controlled trial of an initial strategy of PCI compared to OMT, rather than technique, with subsequent revascularisation considered in patients randomised to OMT permitted in certain specific, protocol-defined circumstances. Consequently, a per-protocol analysis of these data is not planned. However, details of patients in the PCI arm not receiving the intended PCI as planned and those patient in the OMT receiving subsequent revascularisation will be presented.

For the description of procedural outcomes, only patients randomised to PCI and who actually received the PCI will be included in the analysis population.

5.3 Definition of Subgroup Populations in Different Analyses

We plan to undertake a limited number of subgroup analyses for the primary endpoint. Since the subgroup analyses are secondary analyses and exploratory in nature, the trial has not been powered for these. The pre-specified subgroup analysis will be performed on the following variables using Cox proportional hazards model incorporating tests of interaction:

- Age
- The extent of coronary disease (BCIS-JS)
- Degree of LV dysfunction (% LVEF)
- Diabetes
- NYHA class
- Unprotected left main stem disease
- Presence of atrial fibrillation at baseline
- BNP/NT-pro-BNP at baseline

For analysis of BNP/NT-pro-BNP, as different assays have been used across clinical sites, values will be standardised to the upper limit of normal (ULN) for the local assay. Results will be expressed as multiples of the ULN, with subsequent analysis performed using these standardised values.

For the continuous variables of age, degree of LV dysfunction and the standardised values of the BNP/NT-pro-BNP the cutpoint for the subgroup analysis will be at the median value. For the extent of coronary disease and NYHA class two categories will be formed for the subgroup analysis. For NYHA class these will be classes I/II and III/IV, and for extent of coronary disease these will be ≤8 or ≥10 according to BCIS-JS. The remaining variables are binary.

In addition, an analysis will be undertaken according to a patients' underlying risk of the primary outcome. First, univariate Cox proportional hazards model will be used to assess the associations between each potential risk factor and the outcome. Using a forwards modelling approach, risk factors will be entered into a multivariable Cox regression model one at a time, starting with those showing the strongest association with the outcome in the univariable analyses. The final model will include all risk factors that are independently associated with the outcome, as well as an indicator variable for randomised treatment. Using this model, a risk score for the primary outcome will be calculated for each patient (assuming the patients received OMT only) and an interaction term fitted in the Cox model for the primary outcome between risk and treatment to establish if there is greater benefit of PCI according to underlying risk.

In order to assess the effect of PCI by risk, patients will be categorised according to their underlying risk of the primary outcome. The cutpoints for this analysis will be at points such that three groups will be formed with approximately a third of events in each risk group. The number of patients with the primary will be tabulated by treatment group and risk category and corresponding hazard ratios calculated (along with 95% confidence intervals). In addition the absolute risk differences will be calculated at 2 years (the minimum follow-up for each patient) along with 95% confidence intervals and presented in each risk category. Both ratio measures and absolute measures will be utilised to fully assess whether the impact of PCI depends on underlying risk. Absolute differences at other timepoints will also be presented as secondary analyses, to assess whether any benefit, related to risk, increases or decreases over time.

6 Statistical analysis

6.1 General

The final statistical analysis will be performed as pre-specified in this SAP and the planned analyses identified in this SAP will be included in future manuscripts. Any, post-hoc, exploratory analyses completed to support planned analyses, which were not identified in this SAP, will be documented and reported in the relevant trial reports. Any results from unplanned analyses will be clearly identified in the text of the trial reports.

6.2 Pooling of Sites

The data from each participating centre will be pooled for all primary and secondary analyses.

6.3 Interim Analysis

An independent Data and Safety Monitoring Committee (DSMC) was established and a separate DSMC charter developed which includes details of the meeting schedule and stopping guidelines.

6.4 Time Points for Analysis

All final, planned, analyses identified in the protocol and in this SAP will be performed only after the last patient has completed their 2-year follow-up. In addition, no database may be locked, randomisation code unblinded, or analyses completed until this SAP has been approved other than for any pre-specified interim analyses.

6.5 Methods for Handling Withdrawals and Missing Data and Other Issues

6.5.1 Withdrawals

Patients who are randomised but withdraw before the intervention will undergo standard clinical care according to local protocols. Patients will be encouraged to allow data and samples that have been collected before withdrawal to be used in the analysis. However, if consent to use data/samples is also withdrawn, then these will be discarded.

6.5.2 Missing Data

Missing data will be identified and an effort made to return to the original medical records to obtain the data. If this is not possible and the data are missing, modelling

techniques and appropriate missing imputation methods will be considered depending on the scale and the pattern of missing data. See Section 9.2.1 for further details.

6.5.3 Data Transformations

The primary endpoint involves the occurrence or not of an event and so will not require any data transformation for the primary analyses. In the case of secondary endpoints that involve a continuous outcome, the outcome variable will be assessed to establish if a transformation, for example logarithmic, may be necessary to allow for analysis with the most appropriate statistical method. Where possible results will be presented on the original scale to aid in a clinical interpretation.

6.5.4 Multiplicity

There are no planned adjustments to the Type I error for multiple comparisons.

6.5.5 Covariate Adjustment

There are no planned covariate adjustments for the primary analysis or for other event-based outcomes. However, a secondary sensitivity analysis of the primary outcome will be performed adjusting for risk factors derived using the risk score (Section 5.4 and Section 9.1).

There will be adjustment for baseline values of continuous secondary outcomes where indicated in Section 9.2.1.

6.6 Statistical Analytical Issues

The majority of the data analyses will be performed with Stata® version 17.1 or later. Other statistical software may also be used where Stata® does not provide the relevant statistical method.

7 Evaluation of Demographics, Other Baseline Characteristics and PCI Treatment

7.1 General

Continuous variables will be described by the means and standard deviation except for skewed variables which may be described by the median and inter quartile range (IQR). Categorical variables will be described by frequency and percentages in each category by treatment group.

7.2 Demographics

Demographic data collected at baseline include:

- Sex
- Ethnicity – Caucasian, Asian, African-Caribbean, Other
- Date of birth (and age)

7.3 Baseline Physical Examination

Patients will be examined at baseline and the following characteristics recorded:

- Height
- Weight
- Body mass index
- Blood pressure (systolic and diastolic)
- Heart rate
- Smoking history
- ECG if performed (atrial fibrillation, heart rate)
- LVEF (echocardiograph: Simpson's biplane) %
- ICD / CRT inserted

7.4 Screening Medical History

The recipient will have the following baseline disease specific characteristics recorded:

- Previous PCI
- Previous CABG
- Previous stroke
- Angina status (CCS grade 0-4)
- NYHA class
- EQ-5D-5L and KCCQ questionnaires
- Jeopardy score
- Viability assessment method (how assessed)
- Blood tests (Hb, Cr, lipids, BNP/NT-proBNP, HbA1C)
- On dialysis

7.5 Medications

All cardiac medications being taken at randomisation will be summarised as frequency and percentage in each category, by treatment group.

7.6 PCI Treatment

The following PCI treatment specific data are recorded and will be summarised:

- Staging and timing of PCI
- Number of lesions/vessels successfully treated, total number of stents used and type
- Circulatory support, VT/VF requiring defibrillation, cardiorespiratory arrest requiring assisted ventilation, prolonged hypotension
- Bleeding (major/minor), access site complications and acute kidney injury
- Jeopardy score (post PCI, change from baseline)
- Aspirin / additional antiplatelet at discharge
- Days in hospital, time in level 2 environment
- Pre PCI troponin, discharge troponin.
- Creatinine (at discharge, change from baseline)

8 Evaluation of Treatment Compliance

8.1 Compliance with Study Intervention

There is no time limit from randomisation to PCI. However, it is recommended that index PCI be carried out as close as possible to randomisation (as either a one-stage or two-stage procedure) to minimise the incidence of major adverse cardiovascular events prior to the assigned treatment. Details of the treatment procedure are recorded to determine if the intervention was applied as planned. In addition, both treatment arms are allocated OMT. Cardiac medication at each timepoint after baseline up to 2 years follow-up will be described by frequency and percentage in each category, by treatment group, and compared using a Chi-squared test (or Fisher's exact test if expected numbers are less than 5).

9 Evaluation of Efficacy Parameters

9.1 Analysis of Primary Outcome Variable: All-cause Death or Hospitalisation due to Heart Failure

The primary analysis will be a comparison of the incidence of all-cause death or hospitalisation due to heart failure over all available follow-up between the intervention (treatment with PCI) and control (OMT alone) arms of the trial. An

unadjusted time-to-event analysis will be performed on the primary endpoint, with the time to the first event (or censoring) measured from randomisation on an ITT basis. Hazard ratios together with associated confidence intervals will be calculated from the Cox proportional hazards model. A p-value for the treatment difference will be calculated using a likelihood ratio test. The proportionality assumption underlying the Cox model will be assessed via Nelson-Aalen plots by treatment group and by a more formal test comparing treatment hazard ratios across appropriate pre-specified follow-up time bands. If there is clear non-proportionality, comparisons will also be made in early and later follow-up using cut-points at 6 months, 1 year, and 2 years. Cumulative event rates will be calculated and presented using Kaplan-Meier time-to-event curves. As a measure of absolute treatment difference, cumulative event rates based on Kaplan-Meier estimates will be compared at 2 years and a 95% confidence interval for the difference calculated. Losses to follow-up are expected to be minimal and patients will be included up until the time they experience the event or are censored.

A secondary sensitivity analysis will be performed for the primary endpoint using a Cox model adjusted for those risk factors identified in the risk score model developed for subgroup analysis (Section 6.5.5).

9.2 Analysis of Major Secondary Endpoints

Analysis of major secondary outcomes will be based on the ITT population.

9.2.1 LVEF on Echocardiography at 6 Months and 1 Year

Baseline, 6-month and 12-month echocardiograms will be anonymised and submitted to an independent echocardiography core laboratory (at Guy's and St Thomas' Hospital, London, UK), which will determine LVEF using a Simpson's biplane method for evaluation of the major secondary outcome. Anonymised scans were read by core laboratory readers who were blinded to treatment assignment and also the timing of each echo in relation to randomisation.

Where echocardiograms were not performed at precise follow-up intervals (for example, due to restrictions related to the COVID-19 pandemic), echocardiograms recorded between 4 months to 9 months post-randomisation will be considered as the planned 6-month measure, those recorded from 9 months to 24 months post-randomisation will be considered as the planned 1-year measure and the mean (SD) delay from randomisation to each echocardiogram reported. For patients who die a

value of 0% will be imputed for the primary analysis with a secondary sensitivity analysis excluding these patients.

The mean % LVEF at 6 months and 1 year in each treatment group, together with the absolute difference between these measures (and its associated p-value and 95% confidence interval), will be estimated using a linear mixed effects model for repeated measures as described by Frost, Kenward and Fox.[1]

This linear mixed model is an extension of a simpler model suggested by White and Thompson as an alternative to the standard analysis of covariance (ANCOVA) model commonly used for the analysis of clinical trials with only a single follow-up measure of the outcome plus a baseline measure.[2] Key features of the White and Thompson model are that the baseline measure is included in the model as an additional outcome that is allowed to be correlated with the follow-up measure, and whose mean is assumed to be the same in both arms of the trial. In the absence of missing data, the White and Thompson model gives identical effect estimates and virtually identical standard errors to the more standard ANCOVA model. Where there are missing data, the White and Thompson approach has the advantage that patients for whom the follow-up measure is present but the baseline measure is missing can be included in the analysis. It also extends naturally to the model proposed by Frost, Kenward and Fox for the situation where there are multiple follow-up measures.

Like the White and Thompson model, this model includes the baseline measure of % LVEF as an additional outcome variable. It includes time in the model as a categorical variable (baseline, 6 months and 1 year), random intercepts, an interaction between time (categorical) and treatment group, and assumes no effect of treatment at baseline (as with the White and Thompson model). The model uses an unstructured variance-covariance matrix, and allows the baseline and follow-up measures of LVEF to be correlated. This type of model has been used previously to analyse clinical trial data.[3,4] If there were only a single follow-up measure, this model would be equivalent to the White and Thompson model referred to earlier.

It may be necessary to examine % LVEF for transformations to adhere to the assumptions of the linear mixed model or use bootstrapping techniques if a suitable transformation cannot be found.

An analysis of site reported LVEF will also be undertaken as a sensitivity analysis.

9.2.2 Quality of Life Scores

These comprise the Kansas City Cardiomyopathy questionnaire (KCCQ) at 6 months, 1 year and 2 years post randomisation, and the EuroQol EQ-5D-5L at 6 months and then yearly to the end of follow-up.

For both quality of life measures described above, differences in mean levels of the standard outcome measures between treatment groups at each timepoint will be calculated using a linear mixed model with treatment group, categorical treatment by timepoint interaction and baseline standard outcome measure in the model (random intercept, unstructured correlation). It may be necessary to examine the quality of life score for transformations to adhere to the assumptions of the linear mixed model or use bootstrapping techniques if a suitable transformation cannot be found. EuroQol EQ-5D-5L data will also be analysed as part of the health economics outcome (Section 11).

9.2.3 NYHA Functional Class at 6 Months, 1 year and 2 years

NHYA class is an ordinal outcome measure scored as I, II, III or IV. NYHA class will be summarised as frequency and percentage in each category, by treatment group and timepoint. Ordered logistic regression adjusted for baseline NYHA class will be used at each timepoint (conditional on surviving to that point) to compare treatment groups by estimating the proportional odds ratio (95% confidence interval) for a higher NYHA class and associated likelihood ratio test p-value.

As a sensitivity analysis, deaths before a timepoint will be assigned a NYHA class of V and included in the analysis at that timepoint. For each model, the assumption of proportional odds will be assessed graphically by estimating the odds ratio (95% confidence interval) for each cutpoint of the outcome and more formally using the Brant test. If there is clear evidence against the proportional odds assumption, comparisons will also be made by dichotomising the outcome or using appropriate non-parametric methods.[5,6]

9.3 Analysis of Other Secondary Endpoints

Analysis of secondary outcomes will be based on the ITT population unless otherwise stated below.

(1) Cardiovascular death over the entire duration of follow-up
(2) All-cause death over the entire duration of follow-up
(3) Hospitalisation due to heart failure over the entire duration of follow-up

(4) Acute MI over the entire duration of follow-up (until last scheduled annual follow-up visit)
(5) Appropriate ICD therapy to 2 years post randomisation
(6) Unplanned further revascularisation over the entire duration of follow-up (until last scheduled annual follow-up visit)
(7) CCS class at 6 months, 1 year and 2 years post randomisation
(8) NHS Resource use
(9) Brain natriuretic peptide (BNP or NT-Pro BNP) level at 6 months, 1 year and 2 years post randomisation
(10) Major bleeding to 2 years post randomisation (recorded post PCI, 6 months, 1 year and 2 years)

The above secondary time to event outcomes (1-6) will be analysed using the same methods as described for the primary outcome (Section 9.1). CSS class, which is an ordinal outcome measure scored 0-4, will be analysed using the same methods as described for 'NYHA Functional Class' outcome (Section 9.2.3). Analysis of NHS resource use is described in the health economics outcome section (Section 11). Differences in mean BNP or NT-Pro BNP between treatment groups at each timepoint will be calculated using a linear mixed model with treatment group, categorical treatment by timepoint interaction and baseline BNP or NT-Pro BNP in the model (random intercept, unstructured correlation) as described for % LVEF (Section 9.2.1). It may be necessary to examine BNP or NT-Pro BNP for transformations to adhere to the assumptions of the linear mixed model or use bootstrapping techniques if a suitable transformation cannot be found.

Major bleeding will be summarised as the cumulative frequency and percentage at each time point by treatment group. The treatment groups will be compared at 1 and 2 years using a risk ratio, 95% confidence interval and likelihood ratio test p-value from a binomial regression model.

Appropriate ICD therapy will be assessed, on an ITT basis, in the cohort that had undergone device implantation before randomisation or within 90 days of randomisation. The main analysis will only include centre-reported ICD data up to 2 years from randomisation. Longer term ICD-derived outcomes will be the subject of a separate sub-study. See also section 9.6.2 below.

For death, MI, unplanned revascularisation, heart failure hospitalisation and appropriate ICD therapy outcomes, further details of these outcomes will be

summarised by treatment group (e.g. primary causes of death, NSTEMI or STEMI, reason for revascularisation and type of revascularisation, days in hospital, types of therapies). Continuous variables will be described by the means and standard deviation. Alternatively for skewed variables they may be described by the median and IQR. Categorical variables will be described by frequency and percentages in each category.

9.4 Repeat Hospitalisation due to Heart Failure and Death

To take account of recurrent hospitalisations due to heart failure, while also accounting for the competing mortality, a parametric joint frailty model will be used. The aim is to analyse repeat hospitalisations due to heart failure while accounting for their associated mortality risk. This will be achieved by specifying distributions for recurrent hospitalisations due to heart failure as well as for time to death and including individual-specific latent variables to allow for an association between the two event processes. This would yield a hazard ratio and 95% CI for recurrent hospitalisations due to heart failure (which accounts for mortality as informative censoring) as well as a hazard ratio and 95% CI for all-cause mortality. We will also undertake a negative binomial model to mitigate any potential issues in the development with the above model.[7-9]

9.5 Win Ratio for Death, Repeat Hospitalisation due to Heart Failure and KCCQ

The win ratio method will be used to account for the hierarchy of events as well as to incorporate repeat hospitalisations due to heart failure.[10] The win ratio is the ratio of “winners” on PCI compared to “losers” thus a value above 1 indicated a benefit of PCI. Confidence intervals will be calculated as well as p-values and will incorporate all-cause mortality, hospitalisation due to heart failure and KCCQ at two year. The outcome hierarchy is as follows:

(1) Time to all-cause death

(2) Number of hospitalisations due to heart failure

(3) KCCQ score at 2 years

The win ratio will use an unmatched pairs approach with each individual in the PCI arm compared to each individual in the OMT arm, using the stepwise sequence above, to adjudicate a winner/loser or declare a tie. If the comparison comes down to the KCCQ score at 2 years and this metric is not available for at least one of the patients

then a tie will be declared. For each comparison the common follow-up interval will be defined, i.e. follow-up will be censored, at the duration of the shorter follow-up interval of the relevant pair.

9.6 Other Planned Analyses

9.6.1 Impact of COVID-19

The last patient was randomised on 19th March 2020, four days before the first UK-wide lockdown was announced on 23rd March 2020 due to COVID-19. To allow for potential increase in event rates due to the COVID-19 pandemic and the potential impact on follow-up in REVIVED (e.g. increased delay to PCI and challenges in follow-up) additional analyses will be undertaken.

The event rates for all-cause death and hospitalisation due to heart failure will be compared pre- and post- 23rd March 2020 when the lockdown was announced. These analyses will account for time since randomisation. Rates will be compared in follow-up periods since randomisation with cut-points chosen based on the number of events available across follow-up in the pooled treatment groups before the treatment code is broken (e.g. 0-6 months, 6 months to 1 year then beyond 1 year).

An analysis will be undertaken of the primary outcome and component events comparing the impact of the interventions pre- and post- 23rd March 2020. The treatment effect together with 95% confidence intervals will presented by these time periods and formally assessed with an interaction test between time period and treatment from the Cox model.

9.6.2 Implantation of Devices during Follow-up

Date of implantation of ICD or CRT devices after randomisation is collected up to 2 years follow-up. Time to implantation will be compared between treatment groups using the same methods as described for the primary outcome (Section 9.1).

9.6.3 Echocardiography Data Core Laboratory

More detailed exploratory analyses will be performed on echocardiography data sent to the core laboratory at baseline, 6 months and 1 year post randomisation. In addition to LVEF analyses further exploratory analyses on other echocardiographic parameters will also be undertaken.

9.6.4 Acute Myocardial Infarction

For the primary analysis, myocardial infarction will be defined according to the third universal definition of myocardial infarction (UDMI).[11] In order to account for variable definitions of myocardial infarction, secondary sensitivity analyses may be performed with MI defined according to the 4th UDMI and the Society for Cardiovascular Angiology and Interventions (SCAI) definition of periprocedural MI.[12,13]

10 Evaluation of Safety Parameters

10.1 Adverse Events

Adverse events (AE) and serious adverse events (SAE) will be reported as the proportion of patients in each treatment group that suffer an event. These outcomes will be reported using the ITT.

11 Analysis of Health Economic Outcomes

11.1 General

The REVIVED-BCIS2 protocol specifies that a within-trial analysis (WTA) - an analysis using trial data only – will be undertaken to assess the costs and effects of OMT and PCI in study participants. Although WTA is considered to have high internal validity, there is a growing awareness that the findings from any RCT should not be considered in isolation but, in fact, are more valuable from a decision-making context when used in a broader context together with additional evidence. Thus, in addition to conducting the trial-level analysis, a REVIVED-BCIS2 economic analysis will be conducted to extend from the single trial to look at long-term costs and outcomes, using all relevant evidence and available interventions. REVIVED-BCIS2 will thus provide a means of collecting data to support a cost-effectiveness analysis of PCI in heart failure. This extended analysis will incorporate the findings from REVIVED-BCIS2 trial into the wider existing evidence base including other evidence any alternative treatments. Such an analysis requires the development of a decision analytic model to accommodate multiple different information sources and will be detailed in a separate analysis plan.

11.2 Within-trial Analysis Outline

The within-trial economic analysis will be performed using individual patient-level data from the REVIVED-BCIS2 trial. Health resource consumption and costs within the trial will be estimated. The outcome measure will be the EQ-5D index score, via the generic preference-based instrument EQ-5D-5L. The economic analysis will be conducted using the perspective of UK NHS and the Personal Social.[14] The period of analysis is 8 years which is the maximum period of patient follow-up in the trial. Following standard convention in economic evaluation, future costs and health outcomes will be discounted. The analyses will be conducted using appropriate statistical software such as R version 4.1.1 or Stata® version 17.1 or later.

11.1 Analysis Methods

11.1.1 Health Resource Consumption and Costs

Data on resource use will be collected for the entire duration of the trial for each participant. Costs will be calculated for each trial participant as the product of resource units used and the relevant unit cost.

The following types of resource use will be used for cost estimation:

☐ Cost of treatments undertaken (trial or non-trial treatments, e.g. IV diuretics).

☐ Costs of out-patient hospital visits and hospital in-patient stay

☐ Costs of events since discharge e.g. unplanned revascularisation

☐ Costs of cardiac medication e.g. statins

Resource use information will be available from study data record forms (CRFs) for each patient. For each participant the relevant resource use information will include:

☐ Number of hospital admissions without overnight stays due to HF or other reasons

☐ Number of nights in hospital for in-patient stay due to HF or other reasons

☐ The type and number of trial treatments

☐ The type and number of non-trial treatments, including medication

The year of pricing will be of 2021. Unit costs associated with resource use in each treatment arm will be estimated based on the appropriate version of NHS reference costs database and Personal and Social Services Research database. Unit costs of

treatment products will be obtained from the appropriate edition of British National Formulary (BNF). In case the information is not available in the BNF database, product costs will be obtained from the manufacturer.

The results section will summarise the costs by type of resource consumed for the two treatment arms. Descriptive statistics for total costs will also be summarised by trial arm at baseline, 6 months, yearly and end of trial follow-up.

11.1.2 Health Benefits

Health benefits will be considered as the change in individual level health-related quality of life (HRQoL) as measured in terms of 'utility' estimates over the study period. Changes in patient utility level will be measured using EQ-5D-5L questionnaire that evaluates patient's HRQoL on the following five dimensions: mobility, self-care, usual activities, pain/discomfort and anxiety/depression. EQ-5D-5L questionnaire was administered at baseline and also at 6 months, yearly and at the end of trial follow-up period to assess utility level at each time point. A value set for the EQ-5D-5L is now available that reflects the preference of members of the public in England for health states that are defined by the EQ-5D-5L descriptive system.[15] However, a new value set is currently under development. NICE current recommendation on the use of the EQ-5D advises that utilities for the 5L are derived using the mapping function developed by NICE DSU, namely the EQG and the NDB methods (i.e. mapping of the 5L descriptive system data onto the 3L valuation set).[16] Therefore, unless the NICE guidance is changed by the time of our analysis, the mapping will be used to derive utilities for the participants in the REVIVED-BCIS2 trial.

All EQ-5D-5L data will be rechecked to ensure that all scores are within the expected parameter space. As for resource use and costs, EQ-5D index scores will be calculated for each patient over the follow-up period and assessed in its distribution. The results section will summarise the EQ-5D index scores by treatment arm at baseline, 6 months, yearly and end of trial follow-up.

11.1.3 Missing Data

Missing data (for reasons other than censoring) will be evaluated to assess the occurrence of specific patterns. If needed, missing data will be handled through the most adequate methodology in line with the overall statistical analysis plan of REVIVED-BCIS2 study.

11.1.4 Sensitivity Analysis

Sensitivity analysis will be considered in line with the statistical analysis plan. Further sensitivity analysis will also be considered, including investigating the impact of assumptions around resource use and unit costs.

11.2 The Economic Analysis Data Validation

The within-trial economic analysis will use the validated dataset produced for the statistical analysis of the RCT. Data on health services resource use and HRQoL (EQ-5D) will be separately validated for the economic analysis. Further validation of the data will be undertaken as follows:

☐ Participants for whom there are no cost and outcome data other than baseline will be considered as censored a day after randomisation.

☐ Data will be assessed and any queries relating to resource use and quality of life will be communicated to the trial team for clarification. Decisions regarding raised data queries will be made in conjunction with the trial team and clearly documented.

12 References

1. Frost C, Kenward MG, Fox NC. Optimizing the design of clinical trials where the outcome is a rate. Can estimating a baseline rate in a run-in period increase efficiency? *Stat Med*. 2008;27:3717-31.

2. White IR, Thompson SG. Adjusting for partially missing baseline measurements in randomized trials. *Stat Med*. 2005;24:993-1007.

3. MacPherson H, Tilbrook H, Agbedjro D, et al. Acupuncture for irritable bowel syndrome: 2-year follow-up of a randomised controlled trial. *Acupunct Med*. 2017;35:17-23.

4. Mullen M, Jin XY, Child A, et al. Irbesartan in Marfan syndrome (AIMS): a double-blind, placebo-controlled randomised trial. *Lancet*. 2020;394:2263-70.

5. Bath PMW, Geeganage C, Gray LJ, et al. Use of ordinal outcomes in vascular prevention trials. Comparison with binary outcomes in published trials. *Stroke*. 2008;39:2817-2823.

6. Churilov L, Arnup S, Johns H, et al. An improved method for simple, assumption-free ordinal analysis of the modified Rankin Scale using generalized odds ratios. *Int J* Stroke. 2014;9:999-1005.

7. Rogers JK, Pocock SJ, McMurray JJ, et al. Analysing recurrent hospitalizations in heart failure: a review of statistical methodology, with application to CHARM-Preserved. *Eur J Heart Fail*. 2014;16(1):33-40.

8. Pocock SJ, Clayton TC, Stone GW. Design of major randomized trials: part 3 of a 4-part series on statistics for clinical trials. *J Am Coll Cardiol*. 2015;66(24):2757-66.

9. Rogers JK, Yaroshinsky A, Pocock SJ, et al. Analysis of recurrent events with an associated informative dropout time: Application of the joint frailty model. *Stat Med*. 2016;35(13):2195-205.

10. Redfors B, Gregson J, Crowley A, et al. The win ratio approach for composite endpoints: practical guidance based on previous experience. *Eur Hear J*. 2020;41(46):4391-99.

11. Thygesen K, Alpert JS, Jaffe AS, et al. ESC Committee for Practice Guidelines (CPG). Third universal definition of myocardial infarction. *Eur Heart J*. 2012;33(20):2551-67.

12. Thygesen K, Alpert JS, Jaffe AS, et al. ESC Scientific Document Group, Fourth universal definition of myocardial infarction (2018). *Eur Heart J*. 2019; 40(3):237–69.

13. Moussa I, Klein L, Shah B, et al. Consideration of a new definition of clinically relevant myocardial infarction after coronary revascularization. *J Am Coll Cardiol*. 2013;62(17):1563–70.

14. Curtis L, Burns A. Unit Costs of Health and Social Care 2020. *Personal Social Services Research Unit, University of Kent, Canterbury*.

15. Devlin NJ, Shah KK, Feng Y, et al. Valuing health-related quality of life: An EQ-5D-5L value set for England. *Health Econ*. 2018;27(1):7-22.

16. Hernández Alava, M, Pudney, S, Wailoo, A. Estimating the relationship between EQ-5D-5L and EQ-5D-3L: results from an English population study 2020. *Policy Research Unit in Economic Evaluation of Health and Care Interventions. Universities of Sheffield and York. Report 063.*

APPENDIX 1: STUDY FLOW CHART

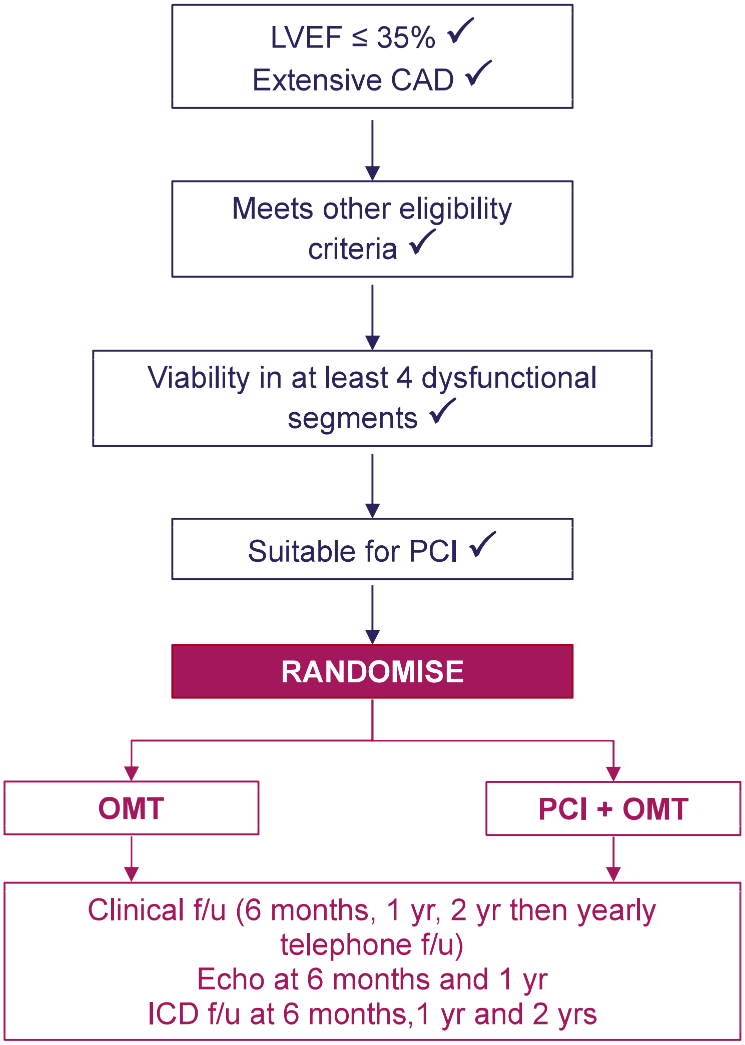

Appendix 2: Summary protocol

Trial Title	Revascularisation for Ischaemic Ventricular Dysfunction (**REVIVED-BCIS2**)
Aim	To evaluate the efficacy and safety of percutaneous coronary intervention (PCI) compared to optimal medical therapy (OMT) alone for ischaemic left ventricular dysfunction
Trial Design	Multicentre prospective randomised open controlled trial
Primary Endpoint	All-cause death or hospitalisation due to heart failure
Secondary Endpoints	Quality of life score:<br>Kansas City Cardiomyopathy Questionnaire (KCCQ)<br>EuroQol EQ-5D-5L<br>New York Heart Association (NYHA) Functional Class<br>Left ventricular ejection fraction (LVEF) on echocardiography at 6 months and 1 year<br>Hospitalisation for heart failure<br>All-cause death<br>Cardiovascular death<br>Acute myocardial infarction (MI)<br>Appropriate implantable cardioverter defibrillator (ICD) therapy<br>Unplanned further revascularisation<br>Canadian Cardiovascular Society (CCS) angina class<br>Health resource use<br>Brain natriuretic peptide (BNP or NT-proBNP) level<br>Troponin (T or I) level<br>Major bleeding
Inclusion Criteria	LVEF ≤35%<br>Extensive coronary artery disease (CAD)<br>Viability in at least 4 dysfunctional myocardial segments, that can be revascularised by PCI
Major Exclusion Criteria	Acute MI <4 weeks prior to randomisation (clinical definition)<br>Acutely decompensated heart failure requiring treatment with inotropes/ventilation/mechanical circulatory support <72 hours prior to randomisation<br>Any contraindication to PCI

Sample Size and Enrolment	n=700<br>Start date: 1st June 2013<br>Recruitment start date: 1st September 2013<br>Recruitment end date: 19th March 2020<br>Follow-up end date: 31st March 2022<br>Number of centres: 40 (listed on trial website)

Appendix 3: Trials procedures

	Tests required for eligibility	Baseline	Pre-PCI as per local protocol ‡	At discharge (up to 16 hours) post-PCI ‡	48 hrs post-PCI ‡	At 6 months after randomisation	At 1 year after randomisation	At 2 years after randomisation	Yearly follow-up	End of trial follow-up
**Clinical assessments (standard of care)**										
Demographics and medical history	X									
Coro Angio	X									
Viability assessment	X									
LVEF Assessment	X*									
Echo		X†								
ICD check		X				X	X	X		
FBC	X			X						
Creatinine‡ & Electrolytes	X			X						
HbA1C		X								
Full Lipid Profile		X								
CK			X	X						
Trop T/I		X	X	X		X	X			
ECG		X		X						
AKI					X					
**Trial specific assessments**										
Echo						X	X			
BNP / NT-proBNP		X				X	X	X		
NYHA/CCS		X				X	X	X		
EQ-5D-5L		X				X	X	X	X	X
KCCQ		X				X	X	X		
Primary Endpoint				X		X	X	X	X	X
Secondary Endpoints						X	X	X	X	
SAEs				X		X	X	X		
Cardiac Medication		X		X		X	X	X		

‡ If PCI is staged, please collect for each stage of the procedure

* In the case of patients with Acute Coronary Syndrome (ACS), must be >4 weeks after ACS

† This echo is only required if there is no available echo within 6 months of randomisation and >4 weeks after ACS

DocuSign Envelope ID: 871CD68E-52C1-4AE7-B406-CA193F8181CC

REVIVED Viability Study

Statistical Analysis Plan

Study Title: Revascularisation for Ischaemic Ventricular Dysfunction (REVIVED-BCIS2)

Study Registration Number: ISRCTN45979711 / NCT01920048

Statistical Analysis Plan Authors: Matthew Ryan, Holly Morgan, Matthew Dodd, Saad Ezad, Amedeo Chiribiri, Tim Clayton, Divaka Perera

Statistical Analysis Plan Version: 2.0 dated 12th December 2022

DocuSign Envelope ID: 871CD68E-52C1-4AE7-B406-CA193F8181CC

CURRENT VERSION APPROVED BY (IF CHANGED):

Name	Signature	Date
Professor Divaka Perera	DocuSigned by: 99D449AE8FF9434...	12/12/2022
Professor Tim Clayton	DocuSigned by: Tim Clayton 2116E431B414456...	12/12/2022

VERSION CHRONOLOGY:			
**VERSION NUMBER**	**EFFECTIVE DATE**	**REASON(S) FOR CHANGE**	**AUTHORISED BY (NAME)**
1.0	29/05/2022	NA	Prof. Divaka Perera<br>Prof. Amedeo Chiribiri
2.0	12/12/2022	1. Includes viability data from dobutamine stress echocardiography (DSE) with prespecified criteria added for adjudicating segmental viability by DSE and rules for patients in whom both tests are available<br>2. Elaborates tests for interaction between viability and a) primary outcome, b) functional recovery.<br>3. Definition of functional recovery changed from an arbitrary numerical value to ≥median change in LVEF within REVIVED trial cohort.<br>4. Secondary outcomes rationalized (myocardial infarction, ventricular arryhthmia and aborted sudden cardiac death removed)<br>5. CMR and DSE appendices added for secondary analyses	Prof. Divaka Perera<br>Prof. Tim Clayton

Table of Contents

1. *SAP history* 5

2. *Background* 5

3. *Study objectives* 6

3.1.1. Primary 6

3.1.2. Secondary 6

4. *Data available* 6

4.1. Sample size 6

4.2. Imaging processing 6

4.3. Other datasets 7

5. *Definitions* 7

5.1. Wall motion scoring 7

5.2. CMR - segmental scar transmurality 7

5.3. DSE – contractile reserve 7

5.4. Patients with CMR and DSE data 7

5.5. Echocardiography – left ventricular morphology and function 8

6. *Outcomes* 8

6.1. Primary outcome 8

6.2. Secondary outcomes 8

6.3. Comparison of CMR and DSE as viability tests 8

6.4. Missing data 8

7. *Statistical analysis* 9

7.1. Viability and event-free survival 9

7.2. Viability and improvement in left ventricular ejection fraction 9

7.3. Improvement in left ventricular function and event-free survival 10

DocuSign Envelope ID: 871CD68E-52C1-4AE7-B406-CA193F8181CC

8. Appendix 1: Cardiac Magnetic Resonance Secondary Analyses 11

8.1. Morphology and function 11

8.1.1. Global parameters 11

8.1.2. Segmental parameters (AHA 17-segment model) 11

8.1.3. Exploratory analyses in subsets 12

8.2. Scar burden and clinical outcomes 12

8.3. Visual vs. quantitative scar burden 12

8.4. Segmental LV function 12

8.5. Regional LV function 13

8.6. Cardiac morphology and clinical outcomes 13

8.7. Inducible ischaemia 13

8.8. Ventricular arrhythmias 14

9. Appendix 2: Dobutamine Stress Echocardiography Secondary Analyses 16

9.1. Analysis of DSE viability to predict outcomes 16

9.2. Wall motion score index prediction of LV recovery and mortality 16

9.3. Relationship between the number of viable segments on DSE and improvement in LV function following revascularisation 16

9.4. Comparison of sustained improvement in wall motion vs biphasic response of segments in predicting LV recovery 17

10. References 18

DocuSign Envelope ID: 871CD68E-52C1-4AE7-B406-CA193F8181CC

1. SAP history

Version 1 of the SAP was signed off prior to database lock and unblinding of the main trial results. Version 2 of the SAP was signed off prior to database lock and unblinding of the imaging core laboratory results.

2. Background

REVIVED-BCIS2 is a multicentre randomised controlled trial investigating whether percutaneous coronary intervention (PCI) improves clinical outcomes in patients with severe left ventricular dysfunction, extensive coronary artery disease and demonstrable myocardial viability, compared with optimal medical therapy (OMT) alone[1]. The primary outcome of the trial is a combination of all-cause and hospitalisation for heart failure (HHF) at a minimum of 2 years follow-up. Key secondary outcomes include change in left ventricular ejection fraction (LVEF) from baseline to 6- and 12-month follow up, as well as the occurrence of myocardial infarction, quality of life scores and healthcare resource utilisation. Enrolment in the trial required demonstration of significant myocardial viability, defined as at least four dysfunctional-but-viable segments which could be revascularised by PCI. Viability needed to be assessed by one of the following 4 modalities: cardiac magnetic resonance imaging (CMR), dobutamine stress echocardiography (DSE), single positron emission computed tomography (SPECT) or positron emission tomography (PET); given wide availability in the United Kingdom, most viability studies were performed with CMR and DSE. When assessing eligibility for enrolment in REVIVED, adjudication of segmental viability was done by recruiting centres, with the trial protocol providing guidance for the definition of viability by each modality.

Viability is defined as regions of dysfunctional myocardium in which function is predicted to improve following appropriate treatment; this has typically been considered specific to revascularisation. Viability assessment with CMR is largely performed with late gadolinium enhancement (LGE) sequences, where myocardial scar is identified by persistent enhancement following injection of gadolinium contrast. Scar burden has been shown to inversely relate to the likelihood of improvement in function at both a segmental and global level[2]. With stress echocardiography, viability is determined by the presence of contractile reserve in response to dobutamine stress, in

myocardial segments which are dysfunctional at rest[3]. The presence of extensive viability is thought to identify patients who gain particular benefit from undergoing revascularisation, though previous studies have been inconclusive and no high-quality data exists to support this presumption[4–6].

3. Study objectives

3.1.1. Primary

To determine whether the extent of viability predicts the effect of PCI on clinical outcomes and left ventricular functional recovery in patients with severe ischaemic cardiomyopathy.

3.1.2. Secondary

To determine the accuracy with which viability imaging predicts global functional recovery

To explore the relationship between viability, revascularisation, functional recovery, and clinical outcome.

4. Data available

4.1. Sample size

In REVIVED-BCIS2 all patients underwent viability imaging prior to randomisation; at least four viable segments which could be revascularised by PCI were required for inclusion. Of the 700 patients enrolled, viability was assessed with CMR in 491 and DSE in 187. Primary outcome data are available for 99.1% of patients. Serial echocardiographic data are available for a subset of these patients.

4.2. Imaging processing

All viability studies will be analysed by one of two dedicated core laboratories: the King's College London Imaging Sciences CMR core laboratory (led by Prof Amedeo Chiribiri) and the King's Health Partners Stress Echo core laboratory (led by Prof Roxy Senior). Analyses will be performed by trained readers blinded to trial-group assignment and any other clinical data. Viability will be analysed on a segmental basis, using the AHA 17-segment model, excluding segment 17 (the left ventricular apex) as it is incompletely imaged on DSE and not included in CMR quantification. Transthoracic echocardiograms were analysed by the Guy's and St Thomas' echocardiography core laboratory with readers also blinded to the temporal sequence of echocardiograms.

DocuSign Envelope ID: 871CD68E-52C1-4AE7-B406-CA193F8181CC

4.3. Other datasets

The following additional datasets will provide comparative data for analysis:

- REVIVED-BCIS2 main trial database and electronic case report forms (London School of Hygiene and Tropical Medicine)
    - All analyses will be based on the intention-to-treat population
- REVIVED-BCIS2 echocardiography core lab database (Guy's and St Thomas' NHS Foundation Trust)
- REVIVED-BCIS2 arrhythmia case report form (King's College London)

5. Definitions

5.1. Wall motion scoring

For CMR and DSE, wall motion score will be classified on a five-point ordinal Likert scale as 1 – normal, 2 – hypokinetic, 3 – akinetic, 4 – dyskinetic, 5 – aneurysmal. For GSTT echocardiography core laboratory (non-DSE) measurements, the latter two points are combined into one category.

5.2. CMR - segmental scar transmurality

Segmental scar transmurality is assessed visually on an ordinal scale as 0%, 1-25%, 26-50%, 51-75% and 76-100% of the segmental extent LGE. For trial inclusion, viability was adjudicated according to local protocols, with a recommendation that dysfunctional segments with ≤ 25% transmurality be considered viable, ≥ 50% non-viable and 25-50% based on local expert opinion. For core-laboratory adjudication, segmental viability was defined as a scar transmurality ≤25% in a segment which is dysfunctional at rest[2,7,8]. Sensitivity analyses will also be performed using a cut-off of ≤50%.

5.3. DSE – contractile reserve

Viability is defined as the presence of contractile reserve in a segment which is dysfunctional at rest. Contractile reserve is defined as an improvement in wall motion score ≥ 1 grade (or ≥ 2 grades in segments which are dyskinetic at rest) during dobutamine stress, compared to baseline.

5.4. Patients with CMR and DSE data

Where participants underwent both CMR and DSE data prior to randomisation, both scans are available and both are interpretable, the CMR data will be used in all analyses.

5.5. Echocardiography – left ventricular morphology and function

Core lab adjudicated resting transthoracic echocardiography-derived values will be used to determine left ventricular volumes and ejection fraction for all analyses that combine or compare CMR and DSE datasets. CMR-derived volumes will be used for analyses restricted to baseline CMR datasets.

6. Outcomes

6.1. Primary outcome

The primary composite outcome is as specified in the main trial SAP and protocol: all-cause death or hospitalisation for heart failure. Analysis will be by the time-to-first event on an intention-to-treat basis.

6.2. Secondary outcomes

While several secondary clinical outcomes were specified in the main trial protocol, for the Viability Study SAP, these will be restricted to all-cause death, cardiovascular death and heart failure hospitalisation, each analysed in the same manner as the primary composite outcome. Improvement of left ventricular function at six months is the key secondary outcome and will be defined in a binary manner, as an improvement in LV ejection fraction greater than the median change in LVEF at six months.

6.3. Comparison of CMR and DSE as viability tests

An exploratory analysis will be used to compare patients who underwent viability testing with DSE and CMR, as well as sensitivity analyses restricted to each modality, for the key outcomes above. Demographic details will be displayed for the two groups as either frequency and percentage, mean ± standard deviation or median (and inter-quartile range) depending on the type of data and normality of distribution. All Cox models concerning myocardial viability will be adjusted for the testing modality used.

6.4. Missing data

Missing viability data will not be imputed – participants whose viability studies are either not available or are not suitable for analysis will be excluded. For patients who have survived more than 6 months, missing values of ejection fraction will be imputed using multiple imputation by chained equations

with randomised treatment, age, sex, and baseline, 6-month and 12-month ejection fraction included in the imputation model. Twenty imputed datasets will be generated and effect estimates and corresponding confidence intervals calculated using Rubin's rules. Other rules for handling missing data will follow the main trial SAP. A sensitivity analysis will be performed limited to patients who have echocardiography data available at baseline and 6-months.

7. Statistical analysis

7.1. Viability and event-free survival

A Cox proportional hazards model will be used to assess the relationship between the number of viable myocardial segments (treated as a linear effect) and the primary outcome. Multivariable Cox analyses will be used to adjust the analysis for factors including age, sex, previous hospitalisation for heart failure, presence of diabetes, ejection fraction, extent of coronary disease, presence of chronic renal failure and modality of viability testing. The interaction between viability and treatment assignment on event-free survival will then be assessed using a Cox proportional hazards model containing the following covariates: viability (treated as a linear effect), randomly assigned treatment, and their interaction, plus the aforementioned list of baseline risk factors. In secondary analyses, the extent of viability will be divided into tertiles and Kaplan-Meier curves stratified by both viability tertile and treatment assignment presented. A sensitivity analysis will be performed where viability is defined irrespective of resting dysfunction (including segments with normal function at rest and with ≤25% scar on CMR or contractile reserve on DSE).

7.2. Viability and improvement in left ventricular ejection fraction

A Cox proportional hazards model will be used to assess the relationship between the number of viable myocardial segments (treated as a linear effect) and recovery of left ventricular function at six and twelve months. Multivariable Cox analyses will be used to adjust the analysis for the list of baseline factors in section 7.1. The interaction between viability and treatment assignment on improvement in left ventricular function will then be assessed using a Cox proportional hazards model containing the following co-variates: viability (treated as a linear effect), randomly assigned treatment, and their interaction, plus the aforementioned list of baseline risk factors. In secondary analyses, the extent of viability will be divided into tertiles and Kaplan-Meier curves stratified by both viability tertile and treatment assignment presented. A sensitivity analysis will be performed where viability is defined irrespective of resting dysfunction (including segments with normal function at rest and with ≤25% scar on CMR or contractile reserve on DSE).

DocuSign Envelope ID: 871CD68E-52C1-4AE7-B406-CA193F8181CC

The relationship between the number of viable segments and recovery of left ventricular function will also be assessed using the change in ejection fraction at 6 and 12 months with a linear mixed effects model for repeated measures adjusting for the variables specified in section 7.1[7]. This model accounts for the baseline ejection fraction, while also incorporating both follow-up measurements, and enables the inclusion of participants with missing measurements. The mean change in ejection fraction at each timepoint along with the association between viability and these means will be estimated. The model includes a variable for time, viability, and an interaction between time and viability, and assumes no effect of treatment at baseline. An unstructured variance-covariance matrix will be used to allow for correlations between repeated measures of ejection fraction. The model will be fitted using restricted maximum likelihood and assumes that missing ejection fraction values are missing-at-random.

7.3. Improvement in left ventricular function and event-free survival

A Cox proportional hazards model will be used to assess the relationship between the improvement in left ventricular function (as a binary effect) on the primary outcome. Multivariable Cox analyses will be used to adjust the analysis for the list of baseline factors in section 7.1. The interaction between improvement in left ventricular function and treatment assignment on even-free survival will then be assessed using a Cox proportional hazards model containing the following co-variates: improvement in left ventricular ejection fraction (treated as a binary effect), randomly assigned treatment, and their interaction, plus the aforementioned list of baseline risk factors. Kaplan-Meier plots will be presented stratified by improvement in left ventricular function and treatment assignment presented. These analyses will be limited to patients who did not experience a primary outcome event in the first six months after randomisation. In secondary analyses, change in left ventricular ejection fraction will be treated as a linear effect.

DocuSign Envelope ID: 871CD68E-52C1-4AE7-B406-CA193F8181CC

8. Appendix 1: Cardiac Magnetic Resonance Secondary Analyses

This appendix describes subsequent planned analyses based on CMR data alone.

8.1. Morphology and function

CMR provides the reference standard assessment of left and right ventricular morphology and function, with improved accuracy and reproducibility over transthoracic echocardiography (TTE)[8]. Specific indices such as LVEF are widely used to inform decisions on pharmacological and device therapy, yet the use of CMR to assess these parameters has not been validated. Whether specific parameters of cardiac morphology or function predict clinical outcome or the benefit from revascularisation with PCI in this population is also unknown.

8.1.1. Global parameters

- LV end diastolic volume*
- LV end systolic volume*
- LV stroke volume*
- LV ejection fraction
- RV end diastolic volume*
- RV end systolic volume*
- RV stroke volume*
- RV ejection fraction
- Left atrial volume*
- Right atrial volume*
- LV mass*
- LV scar percentage
- LV scar mass*

8.1.2. Segmental parameters (AHA 17-segment model)

- End diastolic wall thickness
- End systolic wall thickness
- Wall thickening
- Wall motion scoring (visual likert scale)
- Scar transmurality (visual)
- Scar burden (quantitative)
    - full width half max (FWHM) (primary) and five standard deviation (5SD) method (exploratory)
    - bright blood LGE method (primary) and *dark blood LGE method (exploratory)*

DocuSign Envelope ID: 871CD68E-52C1-4AE7-B406-CA193F8181CC

8.1.3. Exploratory analyses in subsets

- Presence of inducible ischaemia (visual, semi-quantitative, and quantitative analysis, depending on source data availability)
- LV strain
- Extracellular volume
- Quantitative perfusion
- Low-dose dobutamine stress
- Scar texture analysis
- LV geometry, perfusion and scar pattern AI analysis

**indexed to body surface area*

8.2. Scar burden and clinical outcomes

A secondary analysis of the relationship between the number of LV segments with significant scar (defined at exploratory thresholds of ≤25%, <50%, <75% and 75-100% transmural extent), number of non-viable segments, number of segments with normal function, global LV scar burden (%), scar mass (g) and the number of viable, non-revascularised segments (≤25 and ≤50%) will be performed with the same statistical methods as above.

8.3. Visual vs. quantitative scar burden

The extent of myocardial scar burden will be compared between visual and quantitative analysis and FWHM and +/- 5SD data using paired T-tests or the Wilcoxon signed-rank test, depending on the normality of the data.

8.4. Segmental LV function

Improvement in segmental function will be defined as an improvement in wall motion score ≥1 grade at follow-up. The frequency of functional recovery will be compared between segments based on scar transmurality/scar burden across time points using a repeated measures ANOVA to adjust for repeated measures within the same patient and over three time points of baseline, six months and one year. An unadjusted Cox regression model will be used to test for an interaction between scar transmurality/scar burden and the provision of revascularisation and the likelihood of functional recovery, adjusted for baseline segmental wall motion. Two models will be used for revascularisation; the first where all segments will be considered revascularised in patients who were assigned to PCI, the second where only segments where a territory was documented as having been successfully revascularised in the eCRF, with the attribution of segments based on the standard American Heart

DocuSign Envelope ID: 871CD68E-52C1-4AE7-B406-CA193F8181CC

Association 17-segment model adjusted for dominance of the circumflex or right coronary artery [9]. Equivalent analyses will also be performed with scar transmurality/scar burden substituted for end diastolic wall thickness/end systolic wall thickness, as well as for different thresholds of LGE for viability (≤50% and ≤ 75%) and where scar burden is treated as a continuous variable. Receiver-operator characteristic curve analysis will be used to examine diagnostic utility, with Youden's index used to calculate the LGE threshold for optimum sensitivity and specificity to predict functional recovery. DeLong's method will be used to compare ROC curves for the prediction of recovery between the FWHM and 5SD methods of defining regions of myocardial scar.

8.5. Regional LV function

Improvement in regional function will be defined as an improvement in mean wall motion score index ≥ 0.5 across a coronary vascular territory, and by improvement in wall motion score index ≥ the median change. Vascular territories will be defined according to the AHA 17 segment model[9]. The frequency of functional recovery will be compared between territories which were and were not revascularised based upon scar burden, median wall thickness and the proportion of viable segments or ischaemic segments within the subtended territory. A Cox regression analysis will be used to determine the factors best predictive of functional recovery. The analysis will be conducted in three cohorts; the first within the whole trial population, the second solely within those assigned to receive PCI, and the third in all those vascular territories which met trial-defined criteria for viability (median scar burden ≤ 25%).

8.6. Cardiac morphology and clinical outcomes

Median values for left and right ventricular volumes, stroke volume and left ventricular mass will be determined from the dataset. Kaplan-Meier curves for the primary outcome will be constructed separated by median values for each feature of cardiac morphology. A Cox regression analysis will be performed to determine whether these factors have an independent impact on clinical outcome, adjusted for treatment assignment, scar burden and other clinical factors, incorporating tests for interaction.

8.7. Inducible ischaemia

The burden of inducible ischaemia predicts future adverse cardiac events in patients with ischaemic heart disease[10]. CMR allows assessment of ischaemic burden through stress perfusion imaging, typically with adenosine vasodilatation, a diagnostic strategy which is equivalent to both nuclear

perfusion imaging and invasive assessment in patients with stable coronary artery disease and normal left ventricular function[11,12]. Several randomised trials have assessed whether the presence of inducible ischaemia, or the burden of inducible ischaemia, can identify patients with stable coronary artery disease who may benefit from revascularisation, with no significant interaction found in studies to date[13,14]; these trials excluded patients with severely impaired left ventricular function and data the question remains unanswered in this population. Perfusion defects will be defined as delayed subendocardial or transmural contrast inflow for at least five dynamic images after contrast agent arrival in the LV cavity. The perfusion defect needs to extend beyond areas of scar identified on LGE imaging and not be matched on resting images, where available. For quantitative analysis, ischaemia will be defined by absolute myocardial blood flow at stress less than 1.9 ml/min/gram of tissue (or normative values for the specific sequence used)or myocardial perfusion reserve less than 2, where rest images are available.

The extent of inducible ischaemia will be determined on a per-patient basis. Ischaemic burden will be compared with myocardial jeopardy by converting both values to percentages of total LV volume (assuming a jeopardy score of 12 = 100% ischaemic burden. Kaplan-Meier plots for the primary outcome will be constructed based upon the presence/absence of significant inducible ischaemia (at least 1 segment of transmural ischaemia or 2 subendocardial segments in the same coronary territory) and based on the presence of ischaemia in >1 coronary vascular territory and separated by treatment assignment. A Cox regression model will be used to determine the effect of ischaemic burden on the primary outcome, key secondary outcomes and on global functional recovery, adjusted for treatment assignment and other key prognostic variables. A repeated measures ANOVA will compare the likelihood of functional recovery between segments with and without ischaemia, accounting for repeated measures in the same patient. A Cox regression model will be used to test for interaction between the presence of inducible ischaemia and segmental functional recovery, adjusted for segmental scar transmurality, wall thickness and revascularisation status. Further analyses will be performed where segmental ischaemia is defined as either a visible perfusion defect or a dysfunctional and viable segment[15].

8.8. Ventricular arrhythmias

Infarcted myocardial tissue provides an abnormal electrophysiological substrate, which can allow generation of re-entry circuits and ventricular tachycardia and/or fibrillation. Sudden death from these scar-related ventricular arrhythmias is one of the leading causes of death in the ischaemic

DocuSign Envelope ID: 871CD68E-52C1-4AE7-B406-CA193F8181CC

cardiomyopathy cohort. This large, curated cohort provides an invaluable opportunity to assess how scar characteristics predict and relate to arrhythmic risk.

Data relating to indication and timing of ICD and CRT insertion, appropriate and inappropriate therapies and sustained ventricular arrythmias were collected via a dedicated arrhythmia case report form (CRF) during the trial.

The primary outcome of this arrhythmia analysis will be a composite of all-cause death or aborted sudden death (defined as an appropriate ICD therapy or a resuscitated cardiac arrest). A Cox proportional hazards model will be used to assess the relationship between the number of viable segments/volume of myocardial scar and the primary outcome. The analysis will be adjusted for baseline factors listed in section 7.1. The interaction between viability/scar burden and treatment assignment on the primary outcome will then be assessed using a Cox proportional hazards model where randomly assigned treatment, their interaction and the baseline risk factors listed above are included in the model.

Secondary outcomes will include cardiovascular death, appropriate ICD therapies, total number of appropriate therapies and sustained ventricular arrhythmia(s) (any ventricular fibrillation OR ventricular tachycardia >100bpm that lasts for more than 30 seconds OR requires termination in less than 30 seconds due to hemodynamic compromise).

CMR predictors of arrhythmia (scar burden, LV volumes, LV ejection fraction) will be entered into a Cox regression model along with clinical and ECG predictors of risk, to determine which factors best predict the occurrence of sudden death and ventricular arrhythmias.

Exploratory analyses will be performed to relate novel measures such as scar border zone (defined as signal intensity ≥ 2SD around an area of scar) and scar entropy to the occurrence of ventricular arrhythmias[16].

DocuSign Envelope ID: 871CD68E-52C1-4AE7-B406-CA193F8181CC

9. Appendix 2: Dobutamine Stress Echocardiography Secondary Analyses

This appendix describes subsequent planned analyses based on DSE data alone.

9.1. Analysis of DSE viability to predict outcomes

An analysis which reproduces the main viability study outlined above, consisting of the DSE assessed viability alone.

9.2. Wall motion score index prediction of LV recovery and mortality

The wall motion score index (WMSI) is a unitless number that is proportional to the severity and extent of wall motion abnormalities on stress echocardiography calculated by dividing the sum of the individual segment scores by the total number of segments scored. A WMSI >2.19 has been shown to be predictive of mortality or poor functional result in patients undergoing surgical revascularisation however this has yet to be tested in patient undergoing percutaneous coronary revascularisation[17]. ROC curves will be constructed to identify a cut-off WMSI predicting poor outcomes in those undergoing percutaneous revascularisation.

9.3. Relationship between the number of viable segments on DSE and improvement in LV function following revascularisation

Using the AHA 16 segment model for analysis of regional wall motion score will allow the relationship between the total number of viable segments and global improvement in LV function to be explored; additionally segmental recovery will also be assessed using paired student's T test.

DocuSign Envelope ID: 871CD68E-52C1-4AE7-B406-CA193F8181CC

9.4. Comparison of sustained improvement in wall motion vs biphasic response of segments in predicting LV recovery

A biphasic response to dobutamine ("low vs high dose") has been found to be a more sensitive predictor of myocardial viability as compared with sustained improvement, moreover the biphasic response was found to be a predictor of global improvement in LV function after surgical revascularisation[18]. A comparison of sustained improvement vs biphasic response utilising Cox regression analysis will be carried out to identify if a biphasic response is a more sensitive predictor of myocardial recovery following percutaneous coronary revascularisation.

DocuSign Envelope ID: 871CD68E-52C1-4AE7-B406-CA193F8181CC

10. References

1. Perera D, Clayton T, Petrie MC, et al. Percutaneous Revascularization for Ischemic Ventricular Dysfunction: Rationale and Design of the REVIVED-BCIS2 Trial: Percutaneous Coronary Intervention for Ischemic Cardiomyopathy. JACC: Heart Failure 2018;6(6):517–26.

2. Kim RJ, Wu E, Rafael A, et al. The Use of Contrast-Enhanced Magnetic Resonance Imaging to Identify Reversible Myocardial Dysfunction. New Engl J Medicine 2000;343(20):1445–53.

3. Senior R, Lahiri A. Enhanced detection of myocardial ischemia by stress dobutamine echocardiography utilizing the "biphasic" response of wall thickening during low and high dose dobutamine infusion. J Am Coll Cardiol 1995;26(1):26–32.

4. Ryan M, Morgan H, Chiribiri A, Nagel E, Cleland J, Perera D. Myocardial viability testing: all STICHed up, or about to be REVIVED? Eur Heart J 2021;43(2):118–26.

5. Panza JA, Dilsizian V, Curiel RV, Unger EF, Laurienzo JM, Kitsiou AN. Myocardial blood flow at rest and contractile reserve in patients with chronic coronary artery disease and left ventricular dysfunction. J Nucl Cardiol 1999;6(5):487–94.

6. Beanlands RSB, Nichol G, Huszti E, et al. F-18-Fluorodeoxyglucose Positron Emission Tomography Imaging-Assisted Management of Patients With Severe Left Ventricular Dysfunction and Suspected Coronary Disease A Randomized, Controlled Trial (PARR-2). J Am Coll Cardiol 2007;50(20):2002–12.

7. Frost C, Kenward MG, Fox NC. The analysis of repeated 'direct' measures of change illustrated with an application in longitudinal imaging. Statist Med 2004;23(21):3275–86.

8. Gardner BI, Bingham SE, Allen MR, Blatter DD, Anderson JL. Cardiac magnetic resonance versus transthoracic echocardiography for the assessment of cardiac volumes and regional function after myocardial infarction: an intrasubject comparison using simultaneous intrasubject recordings. Cardiovasc Ultrasoun 2009;7(1):38.

9. Cerqueira MD, Weissman NJ, Dilsizian V, et al. Standardized Myocardial Segmentation and Nomenclature for Tomographic Imaging of the Heart. Circulation 2002;105(4):539–42.

10. Bingham SE, Hachamovitch R. Incremental Prognostic Significance of Combined Cardiac Magnetic Resonance Imaging, Adenosine Stress Perfusion, Delayed Enhancement, and Left Ventricular Function Over Preimaging Information for the Prediction of Adverse Events. Circulation 2011;123(14):1509–18.

11. Nagel E, Greenwood JP, McCann GP, et al. Magnetic Resonance Perfusion or Fractional Flow Reserve in Coronary Disease. New Engl J Med 2019;380(25):2418–28.

12. Greenwood JP, Ripley DP, Berry C, et al. Effect of Care Guided by Cardiovascular Magnetic Resonance, Myocardial Perfusion Scintigraphy, or NICE Guidelines on Subsequent Unnecessary Angiography Rates: The CE-MARC 2 Randomized Clinical Trial. Jama 2016;316(10):1051.

DocuSign Envelope ID: 871CD68E-52C1-4AE7-B406-CA193F8181CC

13. Hochman JS, Sleeper LA, Webb JG, et al. Early Revascularization in Acute Myocardial Infarction Complicated by Cardiogenic Shock. New Engl J Medicine 1999;341:625–34.

14. Shaw LJ, Berman DS, Maron DJ, et al. Optimal Medical Therapy With or Without Percutaneous Coronary Intervention to Reduce Ischemic Burden. Circulation 2008;117(10):1283–91.

15. Abdelaty AMSEK, Budgeon CA, Hetherington S, et al. Ischemia and Infarction in Isolated Chronic Total Coronary Artery Occlusion Assessed by Cardiovascular Magnetic Resonance. Jacc Cardiovasc Imaging 2020;14(2):501–2.

16. Gould J, Porter B, Claridge S, et al. Mean entropy predicts implantable cardioverter-defibrillator therapy using cardiac magnetic resonance texture analysis of scar heterogeneity. Heart Rhythm 2019;16(8):1242–50.

17. Klein P, Holman ER, Versteegh MIM, et al. Wall motion score index predicts mortality and functional result after surgical ventricular restoration for advanced ischemic heart failure. Eur J Cardio-thorac 2009;35(5):847–53.

18. Cornel JH, Bax JJ, Elhendy A, et al. Biphasic Response to Dobutamine Predicts Improvement of Global Left Ventricular Function After Surgical Revascularization in Patients With Stable Coronary Artery Disease Implications of Time Course of Recovery on Diagnostic Accuracy. J Am Coll Cardiol 1998;31(5):1002–10.
