# Supplementary material for: Viability and Outcomes With Revascularization or Medical Therapy in Ischemic Ventricular Dysfunction: A Prespecified Secondary Analysis of the REVIVED-BCIS2 Trial
Source: JAMA Cardiol. 2023 Oct 25;8(12):1154–61. doi: 10.1001/jamacardio.2023.3803 (PMC10600721; doi:10.1001/jamacardio.2023.3803)
Supplement: Supplement 2. — eAppendix 1. Revived Sites and Investigators eAppendix 2. Trial Organization and Oversight eFigure 1. Relationship Between Viability Characteristics and the Primary Outcome by Treatment Assignment eFigure 2. Interaction Between Treatment Assignment, Viability Characteristics (in Tertiles) and Primary Outcome eFigure 3. Interaction Between Treatment Assignment, Viability Characteristics (in Tertiles) and Likelihood of Left Ventricular Improvement at 6-Months eFigure 4. Improvement in Left Ventricular Ejection Fraction at 12 Months eFigure 5. Impact of Improvement in Left Ventricular Function at 6 Months on Subsequent Occurrence of Primary Outcome (Landmark Analysis) eTable 1. Baseline Demographics in Patients Who Had CMR, DSE or Were Excluded eTable 2. Primary and Clinical Secondary Outcomes eTable 3. Interaction Between Treatment Assignment, Viability Characteristics (Continuous) and Outcomes eTable 4. Relationship Between Viability Characteristics (Continuous) and Outcomes eTable 5. Sensitivity Analysis (Incorporating 50% LGE Transmurality Threshold) of Interaction Between Treatment Assignment, Viability Characteristics (Continuous) and Primary Outcome eTable 6. Change in Left Ventricular Ejection Fraction From Baseline to 6- and 12-Month Follow-Up eTable 7. Determinants of Binary Improvement in Left Ventricular Ejection Fraction at 6- and 12-Months eReferences. [file jamacardiol-e233803-s002.pdf]

Supplementary Online Content

Perera D, Ryan M, Morgan HP, et al; REVIVED-BCIS2 Investigators. Viability and outcomes with revascularization or medical therapy in ischemic ventricular dysfunction: a prespecified secondary analysis of the REVIVED-BCIS2 trial. *JAMA Cardiol.* 2023;8(12):e233803. doi:10.1001/jamacardio.2023.3803

eAppendix 1. Revived Sites and Investigators

eAppendix 2. Trial Organization and Oversight

eFigure 1. Relationship Between Viability Characteristics and the Primary Outcome by Treatment Assignment

eFigure 2. Interaction Between Treatment Assignment, Viability Characteristics (in Tertiles) and Primary Outcome

eFigure 3. Interaction Between Treatment Assignment, Viability Characteristics (in Tertiles) and Likelihood of Left Ventricular Improvement at 6-Months

eFigure 4. Improvement in Left Ventricular Ejection Fraction at 12 Months

eFigure 5. Impact of Improvement in Left Ventricular Function at 6 Months on Subsequent Occurrence of Primary Outcome (Landmark Analysis)

eTable 1. Baseline Demographics in Patients Who Had CMR, DSE or Were Excluded

eTable 2. Primary and Clinical Secondary Outcomes

eTable 3. Interaction Between Treatment Assignment, Viability Characteristics (Continuous) and Outcomes

eTable 4. Relationship Between Viability Characteristics (Continuous) and Outcomes

eTable 5. Sensitivity Analysis (Incorporating 50% LGE Transmurality Threshold) of Interaction Between Treatment Assignment, Viability Characteristics (Continuous) and Primary Outcome

eTable 6. Change in Left Ventricular Ejection Fraction From Baseline to 6- and 12-Month Follow-Up

eTable 7. Determinants of Binary Improvement in Left Ventricular Ejection Fraction at 6- and 12-Months

eReferences.

This supplementary material has been provided by the authors to give readers additional information about their work.

© 2023 Perera D et al. *JAMA Cardiology.*

eAppendix 1. Revived Sites and Investigators

This list is ordered by the number of patients enrolled by each center. There were many more individuals who made contributions to the REVIVED-BCIS2 Trial at each participating center, but have not been named below; we are very grateful for their efforts.

**Center**	**Principal Investigator**	**Site team**
Guy's & St Thomas' Hospital	Prof Divaka Perera	Prof Amedeo Chiribiri<br>Prof Gerry Carr-White<br>Dr Antonis Pavlidis<br>Prof Simon Redwood<br>Dr Brian Clapp<br>Prof Aldo Rinaldi<br>Dr Haseeb Rahman<br>Dr Natalia Briceno<br>Ms Sophie Arnold<br>Ms Amy Raynsford
Golden Jubilee National Hospital, Glasgow	Prof Mark Petrie	Dr Margaret McEntegart<br>Dr Stuart Watkins<br>Dr Aadil Shaukat<br>Dr Paul Rocchiccioli<br>Ms Louise Cowan
Barts Heart Centre, London	Dr Roshan Weerackody	Dr Ceri Davies<br>Dr Elliot Smith<br>Dr Bhavik Modi
Royal Bournemouth Hospital	Dr Peter O'Kane	Dr Jehangir Din<br>Dr Jonathon Hinton
Leeds General Infirmary	Prof John Greenwood	Dr Jonathan Blaxill<br>Dr Abdul Mozid<br>Ms Michelle Anderson
Royal Victoria Hospital, Belfast	Dr Lana Dixon	Dr Simon Walsh<br>Dr Mark Spence<br>Ms Patricia Glover
Freeman Hospital, Newcastle	Dr Richard Edwards	Dr Adam McDiarmid<br>Dr Mohaned Egred<br>Ms Hannah Stevenson
King's College Hospital, London	Dr George Amin-Youssef	Prof Ajay Shah<br>Prof Theresa McDonagh<br>Dr Jonathan Byrne<br>Dr Nilesh Pareek<br>Mr Jonathan Breeze
Glenfield Hospital, Leicester	Prof Anthony Gershlick	Prof Gerald McCann<br>Dr Andrew Ladwiniec<br>Prof Iain Squire<br>Ms Donna Alexander
Bristol Royal Infirmary	Dr Kalpa De Silva	Dr Julian Strange<br>Dr Tom Johnson<br>Dr Angus Nightingale<br>Ms Laura Gallego
St George's Hospital, London	Prof James Spratt	Dr Claudia Cosgrove<br>Dr Rupert Williams<br>Dr Sam Firoozi<br>Dr Pitt Lim
Pinderfields Hospital, Wakefield	Dr Dwayne Conway	Dr Peter Swoboda<br>Dr Paul Brooksby
New Cross Hospital, Wolverhampton	Dr James Cotton	Dr Richard Horton<br>Ms Stella Metherell

© 2023 Perera D et al. *JAMA Cardiology.*

Kettering General Hospital	Dr Kai Hogrefe	Dr Adrian Cheng<br>Ms Sian Sidgwick
Royal Free Hospital, London	Dr Tim Lockie	Dr Niket Patel<br>Dr Roby Rakhit
Manchester Royal Infirmary	Dr Fozia Ahmed	Dr Cara Hendry<br>Dr Farzin Fath-Odoubadi<br>Dr Douglas Fraser<br>Dr Mamas Mamas
Royal Infirmary of Edinburgh	Dr Miles Behan	Dr Alan Japp
Sunderland Royal Hospital	Dr Nicholas Jenkins	Dr Sam McClure<br>Ms Karen Martin
Wythenshawe Hospital	Dr Eltigani Abdelaal	Dr Jaydeep Sarma<br>Dr Sanjay Sastry<br>Dr Jo Riley
Liverpool Heart and Chest Hospital	Dr Pradeep Magapu	Prof Rod Stables<br>Dr David Wright
Southampton General Hospital	Dr Michael Mahmoudi	Dr Andrew Flett<br>Prof Nick Curzen<br>Ms Sam Gough<br>Ms Zoe Nicholas
Royal Devon & Exeter Hospital	Dr Andrew Ludman	Dr Hibba Kurdi<br>Ms Sam Keenan<br>Mr Kevin Thorpe
University Hospitals Coventry & Warwickshire	Prof Prithwish Banerjee	Dr Luke Tapp<br>Mr Abeesh Panicker
The James Cook University Hospital, Middlesbrough	Dr Mark de Belder	Dr Jeet Thambyrajah<br>Dr Neil Swanson
Lister Hospital, Stevenage	Dr Neville Kukreja	Dr Mary Lynch
Derriford Hospital, Plymouth	Dr Girish Viswanathan	Ms Elaine Jones<br>Ms Sarah Norman
Worcestershire Acute Hospitals	Dr Helen Routledge	Dr Jasper Trevelyan
Worthing Hospital	Dr Nick Pegge	Dr Sukhbir Dhamrait
Salisbury District Hospital	Dr Tim Wells	Dr Manas Sinha
Blackpool Victoria Hospital	Dr Gavin Galasko	Dr Christopher Cassidy
Dorset County Hospital	Dr Tim Edwards	Dr Javed Iqbal<br>Dr Fraser Witherow
Birmingham Heartlands Hospital	Dr Kaeng Lee	Dr James Beattie<br>Dr Mike Pitt
Northern General Hospital, Sheffield	Dr Julian Gunn	Dr Abdallah Al-Mohammad<br>Ms Helen Denney
Queen Alexandra Hospital, Portsmouth	Dr Huw Griffiths	Prof Paul Kalra
Royal Oldham Hospital	Dr Tim Gray	Dr Jolanta Sobolewska
Great Western Hospital, Swindon	Dr Steve Ramcharitar	Ms Laura McCafferty
Ninewells Hospital, Dundee	Dr Thomas Martin	Dr John Irving<br>Dr Zaid Iskandar
Basingstoke & North Hampshire Hospital	Dr Jason Glover	Dr James Beynon
The York Hospital	Mr Maurice Pye	Dr Simon Megarry
North Wales Cardiac Centre	Dr Paul Das	Dr Chris Bellamy

© 2023 Perera D et al. *JAMA Cardiology*.

eAppendix 2. Trial Organization and Oversight

Core Laboratories

Cardiac MRI Core Laboratory

Prof Amedeo Chiribiri (Lead; Reader), King's College London

Dr Pier Giorgio Masci (Reader), King's College London

Dr Sohaib Nazir (Reader), King's College London

Dr Jennifer Silva, King's College London

Dr Ebraham Alskaf, King's College London

Dr Holly Morgan, King's College London

Dobutamine Stress Echocardiography Core Laboratory

Prof Roxy Senior (Lead; Reader), Royal Brompton Hospital, London

Dr Alexandros Papachristidis (Reader), King's College Hospital, London

Dr Navtej Chahal (Reader), Royal Brompton Hospital, London

Dr Rajdeep Khattar (Reader), Royal Brompton Hospital, London

Dr Saad Ezad, King's College London

Echocardiography Core Laboratory

Dr Stam Kapetenakis (Lead), Guy's and St Thomas' Hospital, London

Ms Jane Draper (Reader), Guy's and St Thomas' Hospital, London

Ms Sheila Subbiah (Reader), Guy's and St Thomas' Hospital, London

Ms Annabel Oraa (Reader), Guy's and St Thomas' Hospital, London

Ms Olga Khaleva (Reader), Guy's and St Thomas' Hospital, London

Dr Haotian Gu (Reader), Guy's and St Thomas' Hospital, London

Dr Sarah Blake (Reader), Guy's and St Thomas' Hospital, London

Ms Emily Denman (Reader), King's College Hospital, London

Ms Almira Whittaker (Reader), King's College Hospital, London

Ms Marilou Huang (Reader), King's College Hospital, London

Ms Sandya Nandakumar (Reader), King's College Hospital, London

© 2023 Perera D et al. *JAMA Cardiology.*

Dr Joseph Okafor (Reader), Guy's and St Thomas' NHS Foundation Trust, London

Dr Oleksandr Danylenko (Reader), Guy's and St Thomas' NHS Foundation Trust, London

Committees and Oversight

Trial Steering Committee

Prof Andrew Clark, Chair of Clinical Cardiology, Castle Hill Hospital, Hull (Chair)

Mrs Helen Williams, Pharmacist, NHS Southwark Clinical Commissioning Group, London

Dr Pablo Perel, Epidemiologist, London School of Hygiene & Tropical Medicine

Dr David Walker, Consultant Cardiologist, Conquest Hospital, St. Leonards-on-Sea

Prof Rod Stables, Consultant Cardiologist, Liverpool Heart and Chest Hospital

Prof Divaka Perera, Chief Investigator, King's College London

Ms Liz Bestic, Patient, Carer and Public representative

Mrs Paula Young, Patient, Carer and Public representative

Mrs Helen Datta, Patient, Carer and Public representative

Mr Jeremy Dearling, Patient, Carer and Public representative

Data and Safety Monitoring Committee

Prof Peter Ludman, Consultant Cardiologist, Queen Elizabeth Hospital, Birmingham (Chair)

Dr Suzanna Hardman, Consultant Cardiologist, The Whittington Hospital, London

Prof Louise Brown, Senior Statistician, MRC Clinical Trials Unit at University College London

Clinical Events Committee

Prof Roxy Senior, Professor of Cardiology, Royal Brompton Hospital, London (Chair)

Dr Zaheer Yousef, Consultant Cardiologist, University Hospital of Wales

Dr Rajan Sharma, Consultant Cardiologist, St George's Hospital, London

Dr Shazia Hussain, Consultant Cardiologist, University Hospitals of Leicester NHS Trust

Dr Stephen Hoole, Consultant Cardiologist, Royal Papworth Hospital

Dr Ninian Lang, Reader in Cardiology, University of Glasgow

Dr Kieran Docherty, Clinical Lecturer in Cardiology, University of Glasgow

Dr Roy Gardner, Consultant Cardiologist, Golden Jubilee National Hospital, Glasgow

Prof Andrew Sharp, Consultant Cardiologist, University Hospital of Wales

© 2023 Perera D et al. *JAMA Cardiology.*

Dr Ricardo Petraco, Consultant Cardiologist, Imperial College Healthcare NHS Trust

Dr Vasileios Panoulas, Consultant Cardiologist, Royal Brompton and Harefield Hospitals

Dr Andreas Schuster, Consultant Cardiologist, Universitätsmedizin Göttingen, Germany

Dr Kaleab Asrress, Consultant Cardiologist, Bankstown-Lidcombe Hospital, Australia

Dr Matthew Lee, Clinical Lecturer in Cardiology, University of Glasgow

Prof Pardeep Jhund, Professor of Cardiology and Epidemiology, University of Glasgow

Dr Eugene Connolly, Director, Global Clinical Trial Partners, Glasgow

Prof Raj Kharbanda, Consultant Cardiologist, John Radcliffe Hospital, Oxford

Ms Farandeep Dhaliwal, London School of Hygiene & Tropical Medicine (Admin)

Project Management Group

Prof Divaka Perera, King’s College London

Prof Tim Clayton, London School of Hygiene & Tropical Medicine

Mr Richard Evans, London School of Hygiene & Tropical Medicine

Ms Ruth Canter, London School of Hygiene & Tropical Medicine

Mr Steven Robertson, London School of Hygiene & Tropical Medicine

Mrs Sophie Arnold, Guy’s and St Thomas’ Hospital, London

Dr Bhavik Modi, King's College London

Dr Matthew Ryan, King's College London

Dr Holly Morgan, King’s College London

Mrs Rosemary Knight, London School of Hygiene & Tropical Medicine

Miss Rebecca Matthews, London School of Hygiene & Tropical Medicine

Mrs Lucy Clack, Guy’s and St Thomas’ Hospital, London

Ms Josenir Astarci, London School of Hygiene & Tropical Medicine (Admin)

Trial Statisticians

Ms Joanne Dobson, London School of Hygiene & Tropical Medicine

Mr Matthew Dodd, London School of Hygiene & Tropical Medicine

Prof Tim Clayton, London School of Hygiene & Tropical Medicine

© 2023 Perera D et al. *JAMA Cardiology.*

eFigure 1. Relationship between viability characteristics and the primary outcome by treatment assignment

Figure e1A – The extent of viable myocardium (continuous)

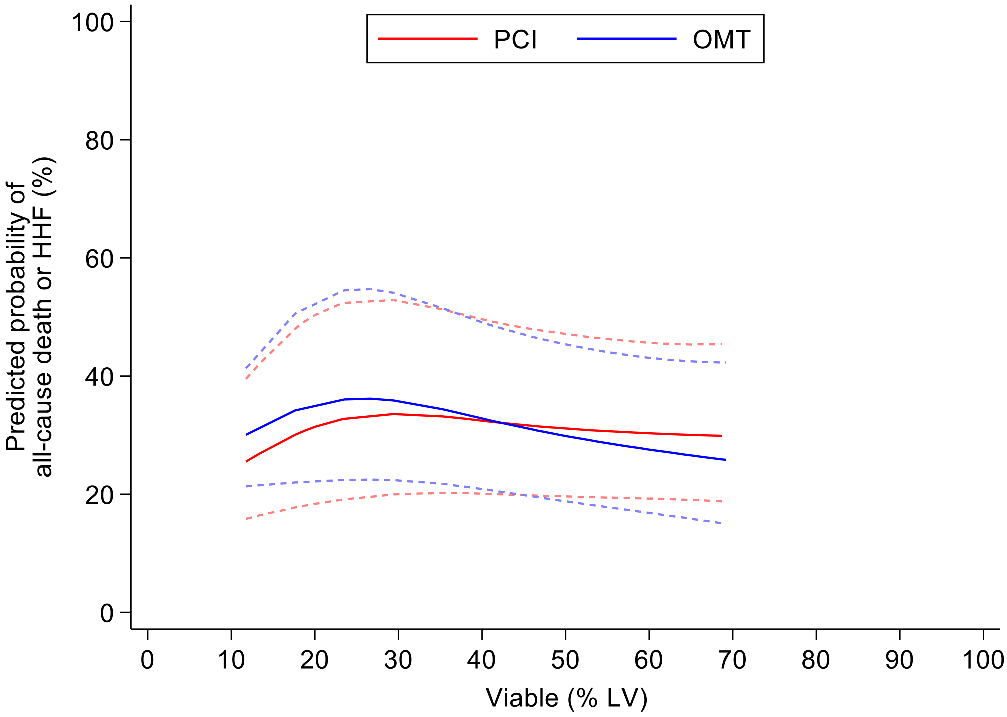

OMT – optimal medical therapy. PCI – percutaneous coronary intervention. Data are presented as cubic splines – these were not specified in the statistical analysis plan but are presented for clarity of visualisation of the data. Dotted lines represent 95% confidence intervals.

© 2023 Perera D et al. *JAMA Cardiology.*

Figure e1B – The extent of non-viable myocardium (continuous)

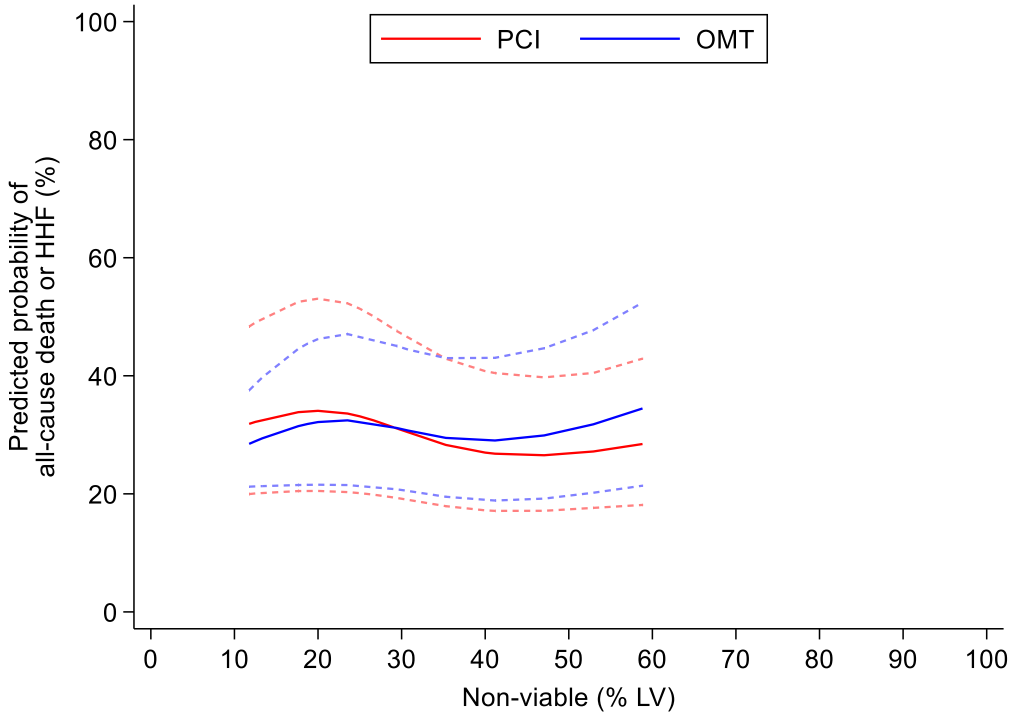

OMT – optimal medical therapy. PCI – percutaneous coronary intervention. Data are presented as cubic splines – these were not specified in the statistical analysis plan but are presented for clarity of visualisation of the data. Dotted lines represent 95% confidence intervals.

© 2023 Perera D et al. *JAMA Cardiology*.

Figure e1C – Scar burden (continuous)

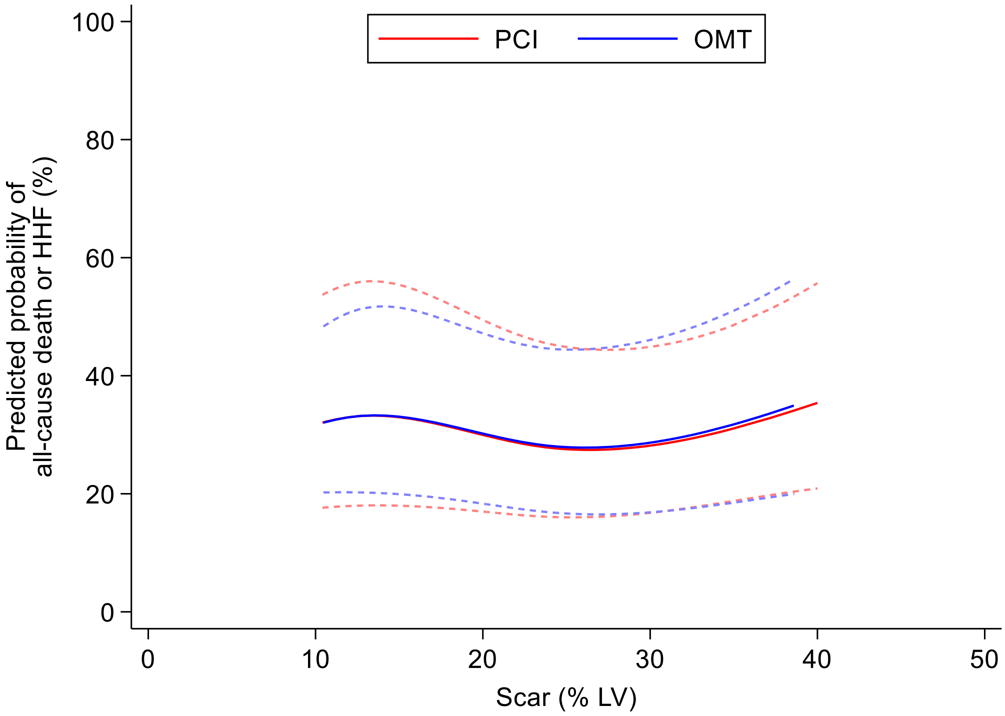

OMT – optimal medical therapy. PCI – percutaneous coronary intervention. Data are presented as cubic splines – these were not specified in the statistical analysis plan but are presented for clarity of visualisation of the data. Dotted lines represent 95% confidence intervals.

© 2023 Perera D et al. *JAMA Cardiology.*

eFigure 2. Interaction between treatment assignment, viability characteristics (in tertiles) and primary outcome

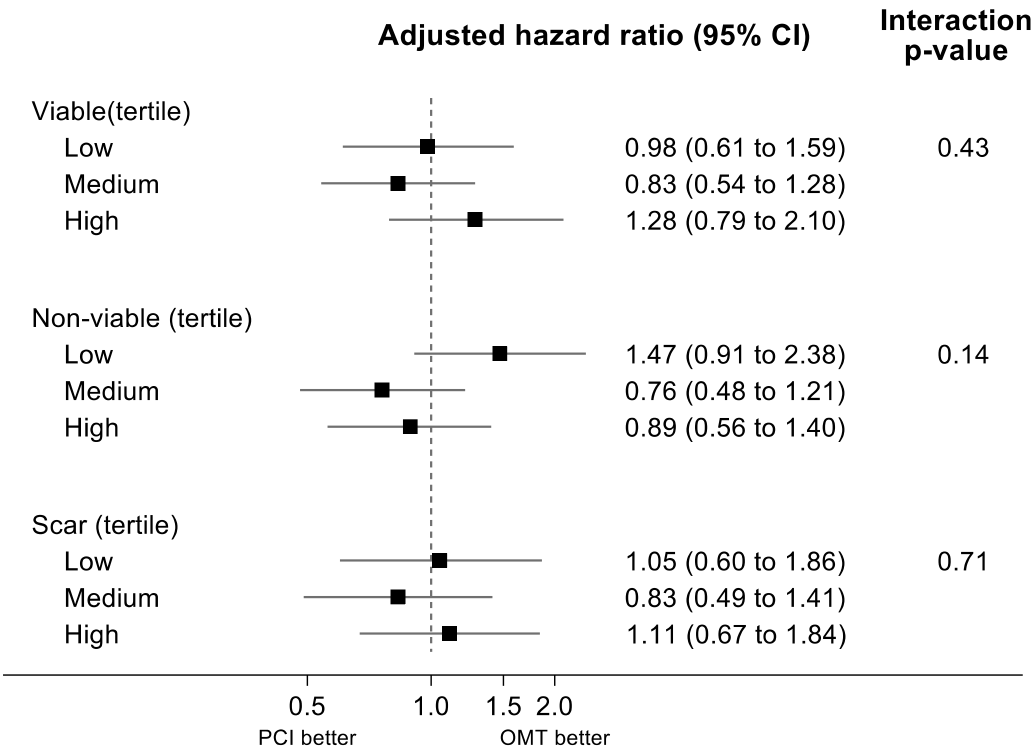

CI – confidence interval. OMT – optimal medical therapy. PCI – percutaneous coronary intervention

Viable myocardium tertiles – low ≤18%, medium >18 to ≤ 41%, high > 41% of LV

Non-viable myocardium tertiles – low ≤ 18%, medium ≤ 35%, high > 35% of LV

Scar burden tertiles – low ≤ 12%, medium >12 to ≤ 24%, high > 24% of LV

© 2023 Perera D et al. *JAMA Cardiology.*

eFigure 3. Interaction between treatment assignment, viability characteristics (in tertiles) and likelihood of left ventricular improvement at 6-months

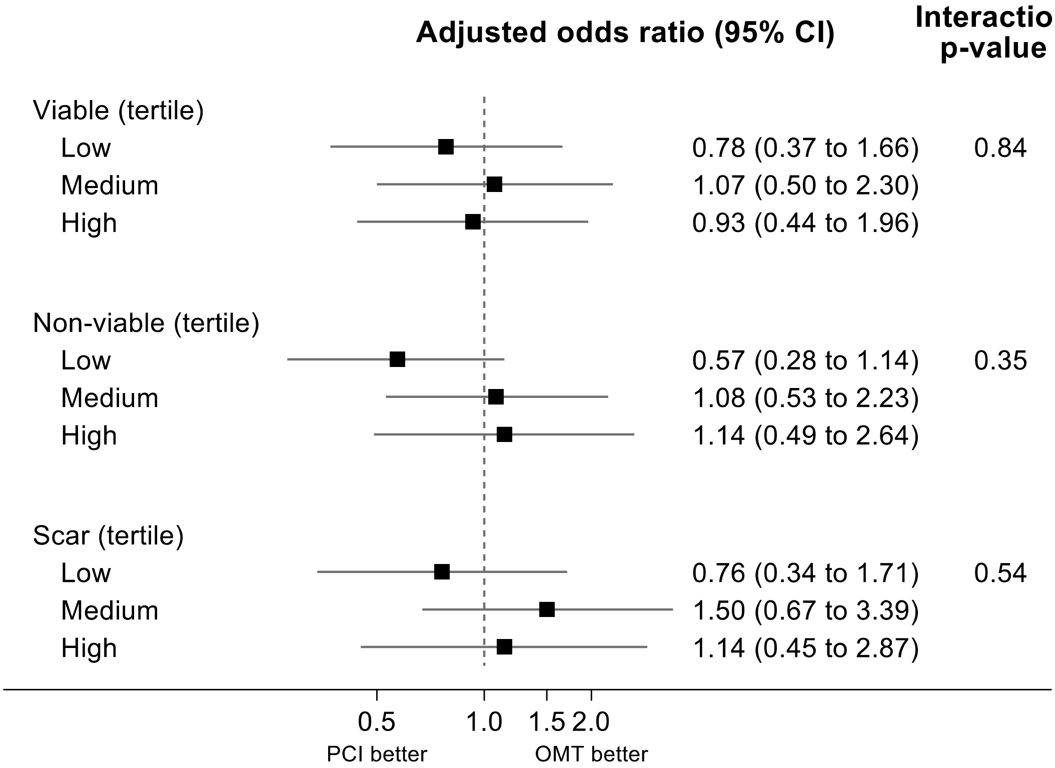

CI – confidence interval. OMT – optimal medical therapy. PCI – percutaneous coronary intervention.
Viable myocardium tertiles – low ≤18%, medium >18 to ≤ 41%, high > 41% of LV mass
Non-viable myocardium tertiles – low ≤ 18%, medium ≤ 35%, high > 35% of LV mass
Scar burden tertiles – low ≤ 12%, medium >12 to ≤ 24%, high > 24% of LV mass

© 2023 Perera D et al. *JAMA Cardiology.*

eFigure 4. Improvement in left ventricular ejection fraction at 12 months

e4A – Improvement in left ventricular function at 12-months by viability characteristics (continuous) in the whole population

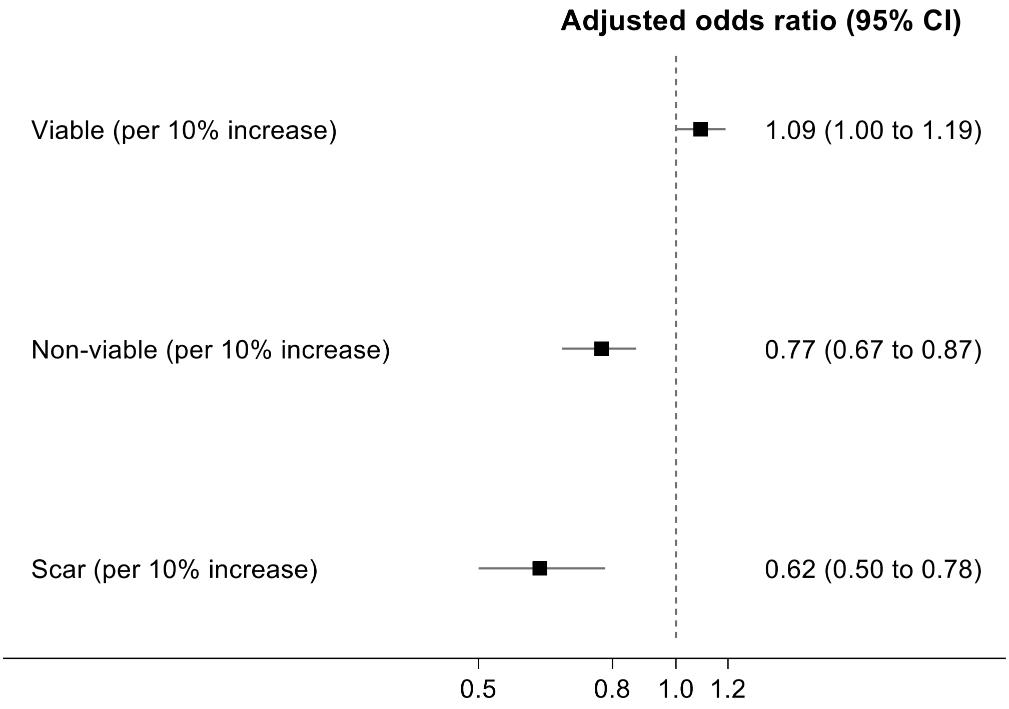

CI – confidence interval. *Per 10% increase refers to an absolute increase in viable/non-viable/scarred myocardium as a percentage of total left ventricular myocardial volume.*

© 2023 Perera D et al. *JAMA Cardiology.*

e4B – Interaction between treatment assignment, viability characteristics (in tertiles) and likelihood of left ventricular improvement at 12 months

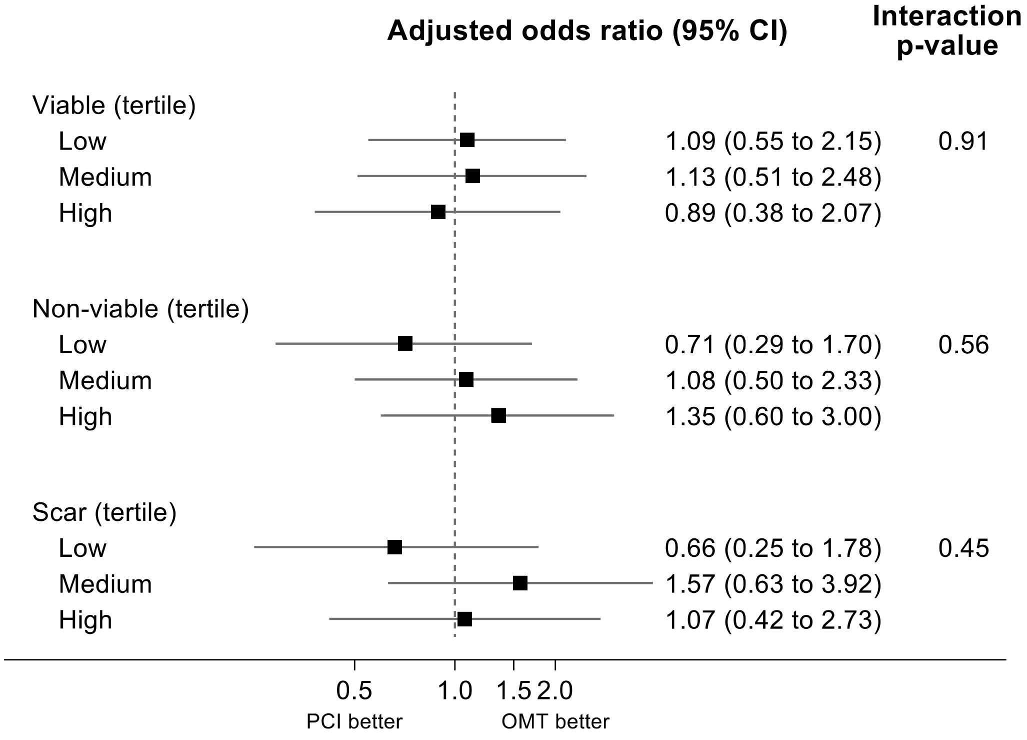

CI – confidence interval. OMT – optimal medical therapy. PCI – percutaneous coronary intervention.
Viable myocardium tertiles – low ≤18%, medium >18 to ≤ 41%, high > 41% of LV
Non-viable myocardium tertiles – low ≤ 18%, medium ≤ 35%, high > 35% of LV
Scar burden tertiles – low ≤ 12%, medi.um >12 to ≤ 24%, high > 24% of LV

© 2023 Perera D et al. *JAMA Cardiology*.

eFigure 5. Impact of improvement in left ventricular function at 6 months on subsequent occurrence of primary outcome (landmark analysis)

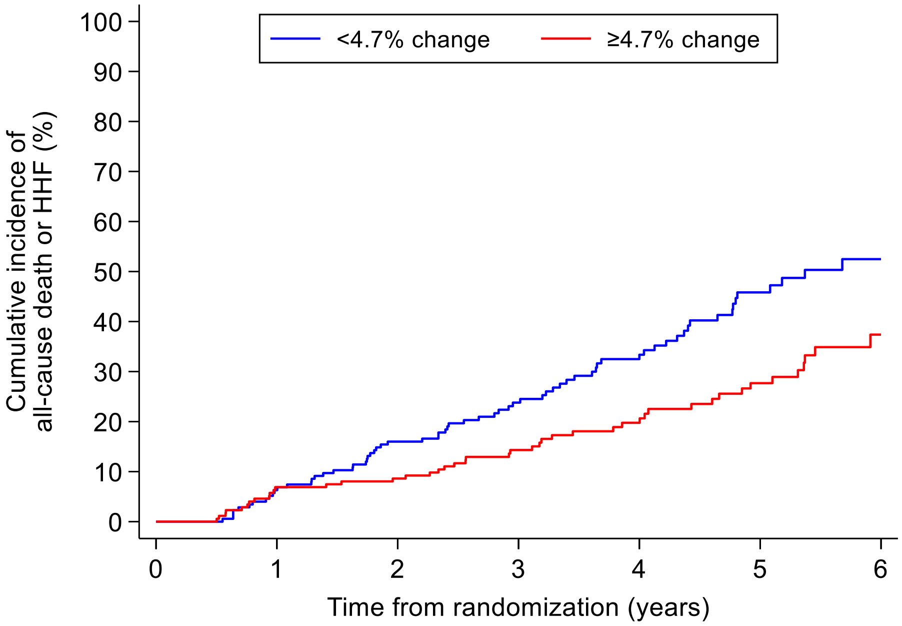

Number at risk							
<4.7% change	175	164	146	106	77	43	18
≥4.7% change	174	162	157	121	91	63	25

The data above only include patients for whom paired baseline and 6-month echocardiograms were available (without imputation). The imputed datasets (n=578 and n=554 at 6- and 12-months respectively) have been used when calculating the odds ratios of the primary outcome by LV improvement in the main manuscript. Patients who experienced a primary outcome event in the first six months were excluded from the imputed and non-imputed analyses. See also table S7.

© 2023 Perera D et al. *JAMA Cardiology.*

eTable 1. Baseline demographics in patients who had CMR, DSE or were excluded*

	CMR (n=479)	DSE (n=131)	Not included (n=90)
Age - yrs	69.5±9.0	68.3±8.7	70.1±9.6
Male – no (%)	419 (87.5)	116 (88.6)	79 (87.8)
Race – no (%)			
White	438 (91.4)	119 (90.8)	77 (85.6)
Asian	33 (6.9)	6 (4.6)	10 (11.1)
Black	4 (0.8)	2 (1.5)	0 (0.0)
Mixed, other or not reported	4 (0.8)	4 (3.1)	3 (3.3)
Diabetes – no (%)	199 (41.5)	51 (38.9)	39 (43.3)
History of MI – no (%)	251 (52.4)	70 (53.4)	51 (56.7)
Previous PCI – no (%)	91 (19.0)	28 (21.4)	23 (25.6)
Previous CABG – no (%)	23 (4.8)	8 (6.1)	3 (3.3)
NYHA class			
I/II	356 (74.3)	103 (81.8)	54 (60)
III/IV	123 (25.7)	23 (18.3)	36 (40)
Angina status			
No angina	314 (65.6)	94 (73.4)	56 (62.2)
CCS I/II	154 (32.2)	32 (25.0)	32 (35.6)
CCS III/IV	11 (2.3)	2 (1.6)	2 (2.2)
Median BCIS jeopardy score (IQR) †	10 (8 to 12)	10 (8 to 12)	8 (6 to 12)
Left ventricular ejection fraction - %‡	31.4±9.7	33.8±9.9	31.1±10.7
Implantable defibrillator at randomisation – no. (%)	87 (18.2)	36 (27.5)	25 (27.8)
ICD	45 (9.4)	18 (13.7)	16 (17.8)
CRT-D	42 (8.8)	18 (13.7)	9 (10.0)
Left main coronary artery disease – no (%)	71 (14.9)	15 (11.5)	9 (10.0)
Cardiac medication – no (%)			
RAAS inhibitor	426 (89.1)	114 (87.0)	80 (88.9)
Beta-blocker	428 (89.4)	123 (93.9)	83 (92.2)
Mineralocorticoid receptor antagonist	229 (48.0)	75 (57.3)	42 (47.2)
Median extent of viable myocardium - % (IQR)$	29 (18 to 47)	35 (12 to 59)	-
Median extent of non-viable myocardium- % (IQR)$	29 (12 to 41)	35 (18 to 47)	-
Scar burden - % (IQR)	18 (9 to 28)	-	-

* Plus–minus values are means ±SD. Percentages may not total 100 because of rounding. 19 patients underwent assessment with single photon emission computed tomography and 11 with positron emission tomography. BCIS denotes British Cardiovascular Intervention Society, CMR cardiovascular magnetic resonance imaging, CRT cardiac resynchronization therapy, DSE dobutamine stress echocardiography, ICD implantable cardioverter defibrillator, IQR interquartile range, RAAS renin angiotensin aldosterone system.

© 2023 Perera D et al. *JAMA Cardiology*.

† The British Cardiovascular Intervention Society (BCIS) jeopardy score is a quantification of the extent of myocardial jeopardy relating to clinically significant coronary artery stenoses. The score ranges from 0 (no significant coronary disease) to 12 (disease jeopardizing the whole left ventricular myocardium).

‡Baseline left ventricular ejection fraction measured by the blinded echocardiography core laboratory.

$ 16 (5.4%) patients in the PCI group and 19 (6.0%) patients in the OMT group had non-ischemic scar. The median (IQR) number of segments with non-ischemic scar in these patients was 2 (1 to 3) segments the PCI group and 2 (1 to 3) in the OMT group.

© 2023 Perera D et al. *JAMA Cardiology*.

eTable 2. Primary and clinical secondary outcomes*

	PCI group (n=295)	OMT group (n=315)	Hazard ratio (95% CI)	p-value
All-cause death or hospitalisation for heart failure	107 (36.3)	114 (36.2)	0.99 (0.76 to 1.29)	0.93
All-cause death	91 (30.9)	98 (31.1)	0.96 (0.72 to 1.28)	0.79
Cardiovascular death	64 (21.7)	75 (23.8)	0.88 (0.63 to 1.23)	0.47
Hospitalisation for heart failure	37 (12.5)	47 (14.9)	0.84 (0.54 to 1.29)	0.42
Acute myocardial infarction	31 (10.5)	34 (10.8)	0.99 (0.61 to 1.61)	0.96
Periprocedural	14 (45.2)	0 (0)		
Spontaneous	15 (48.4)	30 (88.2)		
Sudden death	2 (6.5)	4 (11.8)		
Unplanned revascularization	10 (3.4)	34 (10.8)	0.30 (0.15 to 0.61)	0.0003
PCI	9 (90.0)	26 (76.5)		
CABG	1 (10.0)	8 (23.5)		

CI – confidence interval. OMT – optimal medical therapy. PCI – percutaneous coronary intervention. CABG – coronary artery bypass graft surgery

* These are primary and secondary outcomes specific to the subset of patients (n=610) included in the current analysis and hence the values may differ from those previously reported for the overall trial (n=700).

© 2023 Perera D et al. *JAMA Cardiology*.

eTable 3. Interaction between treatment assignment, viability characteristics (continuous) and outcomes

Myocardial Characteristic	Outcome measure	PCI group HR/OR; 95% CI	OMT group HR/OR; 95% CI	Interaction P value
**Viable**	Death or HHF	1.01 (0.94 to 1.09)	0.96 (0.90 to 1.03)	0.33
per 10% absolute	All-cause death	1.01 (0.93 to 1.10)	0.95 (0.87 to 1.03)	0.26
increase by LV	CV death	1.02 (0.92 to 1.12)	0.94 (0.85 to 1.03)	0.22
myocardial	HHF	1.01 (0.90 to 1.15)	0.92 (0.82 to 1.05)	0.30
volume	LV improvement	1.01 (0.89 to 1.14)	1.02 (0.90 to 1.16)	0.92
**Non-viable**	Death or HHF	1.01 (0.92 to 1.12)	1.13 (1.03 to 1.24)	0.11
per 10% absolute	All-cause death	1.02 (0.92 to 1.14)	1.17 (1.05 to 1.29)	0.07
increase by LV	CV death	1.03 (0.91 to 1.17)	1.22 (1.08 to 1.37)	0.053
myocardial	HHF	0.94 (0.80 to 1.12)	1.12 (0.96 to 1.30)	0.14
volume	LV improvement	0.88 (0.75 to 1.04)	0.77 (0.65 to 0.91)	0.24
**Scar burden**	Death or HHF	1.14 (0.96 to 1.36)	1.21 (1.02 to 1.42)	0.64
per 10% absolute	All-cause death	1.14 (0.95 to 1.37)	1.29 (1.08 to 1.54)	0.33
increase by LV	CV death	1.20 (0.97 to 1.48)	1.37 (1.11 to 1.68)	0.38
myocardial	HHF	1.13 (0.84 to 1.53)	1.09 (0.84 to 1.43)	0.86
volume	LV improvement	0.72 (0.54 to 0.96)	0.66 (0.50 to 0.88)	0.68

CI – confidence interval; CV- cardiovascular; HHF- hospitalization for heart failure; HR- hazard ratio; LV – left ventricle; OMT – optimal medical therapy; OR - odds ratio; PCI – percutaneous coronary intervention

© 2023 Perera D et al. *JAMA Cardiology.*

eTable 4. Relationship between viability characteristics (continuous) and outcomes

Myocardial Characteristic	Outcome measure	Association<br>HR/OR; 95% CI
**Viable**<br>*per 10% absolute increase by LV myocardial volume*	Death or HHF	0.98 (0.93 to 1.04)
	All-cause death	0.98 (0.92 to 1.04)
	CV death	0.97 (0.91 to 1.04)
	HHF	0.96 (0.88 to 1.05)
	LV improvement	1.01 (0.93 to 1.11)
**Non-viable**<br>*per 10% absolute increase by LV myocardial volume*	Death or HHF	1.07 (1.00 to 1.15)
	All-cause death	1.10 (1.02 to 1.18)
	CV death	1.13 (1.03 to 1.23)
	HHF	1.04 (0.93 to 1.17)
	LV improvement	0.82 (0.73 to 0.93)
**Scar burden**<br>*per 10% absolute increase by LV myocardial volume*	Death or HHF	1.18 (1.04 to 1.33)
	All-cause death	1.21 (1.07 to 1.38)
	CV death	1.28 (1.10 to 1.49)
	HHF	1.11 (0.91 to 1.36)
	LV improvement	0.69 (0.56 to 0.84)

CI – confidence interval; CV- cardiovascular; HHF- hospitalization for heart failure; HR- hazard ratio; LV – left ventricle; OMT – optimal medical therapy; OR - odds ratio; PCI – percutaneous coronary intervention

© 2023 Perera D et al. *JAMA Cardiology.*

eTable 5. Sensitivity analysis (incorporating 50% LGE transmurality threshold) of interaction between treatment assignment, viability characteristics (continuous) and primary outcome

	Adjusted hazard ratio (95% CI)	Interaction p-value
Viable (per 10% LV increase)	1.01 (0.95 to 1.06)	-
PCI group	1.03 (0.95 to 1.11)	0.52
OMT group	0.99 (0.92 to 1.07)	
Non-viable (per 10% LV increase)	1.04 (0.96 to 1.13)	-
PCI group	0.99 (0.89 to 1.11)	0.21
OMT group	1.08 (0.98 to 1.20)	

CI – confidence interval. LV – left ventricle. OMT – optimal medical therapy. PCI – percutaneous coronary intervention

© 2023 Perera D et al. *JAMA Cardiology*.

eTable 6. Change in left ventricular ejection fraction from baseline to 6- and 12-month follow-up

**LVEF (%) – 6 months**	**PCI (n=277)**	**OMT (n=301)**	**Overall (n=578)**
Baseline, mean (SE)	32.1 (0.7)	32.1 (0.7)	32.1 (0.5)
6 months, mean (SE)	36.5 (0.7)	38.0 (0.7)	37.2 (0.5)
Change, mean (SE)	4.3 (0.9)	5.8 (0.8)	5.1 (0.6)
Change, median (IQR)	4.5 (-3.2 to 11.9)	4.9 (-1.2 to 12.9)	4.7 (-2.2 to 12.5)
**LVEF (%) – 12 months**	**PCI (n=271)**	**OMT (n=283)**	**Overall (n=554)**
Baseline, mean (SE)	32.3 (0.7)	32.3 (0.7)	32.3 (0.5)
12 months, mean (SE)	38.0 (0.7)	37.8 (0.7)	37.9 (0.5)
Change, mean (SE)	5.8 (0.9)	5.5 (0.9)	5.6 (0.6)
Change, median (IQR)	5.4 (-2.9 to 14.6)	5.0 (-3.6 to 14.1)	5.2 (-3.2 to 14.2)

IQR - interquartile range. OMT – optimal medical therapy. PCI – percutaneous coronary intervention. SE - standard error.

Data above include imputation for missing values, as described in the main manuscript

© 2023 Perera D et al. *JAMA Cardiology.*

eTable 7. Determinants of binary improvement in left ventricular ejection fraction at 6- and 12-months

Table e7A – Interaction between treatment assignment, viability characteristics (continuous) and likelihood of left ventricular improvement at 6-months

	**Adjusted odds ratio (95% CI)**	**Interaction p-value**
Viable (per 10% LV increase)	1.01 (0.93 to 1.11)	-
PCI group	1.01 (0.89 to 1.14)	0.92
OMT group	1.02 (0.90 to 1.16)	
Non-viable (per 10% LV increase)	0.82 (0.73 to 0.93)	-
PCI group	0.88 (0.75 to 1.04)	0.24
OMT group	0.77 (0.65 to 0.91)	
Scar (per 10% LV increase)	0.69 (0.56 to 0.84)	
PCI group	0.72 (0.54 to 0.96)	0.68
OMT group	0.66 (0.50 to 0.88)	

CI – confidence interval. LV – left ventricle. OMT – optimal medical therapy. PCI – percutaneous coronary intervention

Table e7B – Sensitivity analysis (incorporating 50% LGE transmurality threshold): Interaction between treatment assignment, viability characteristics (continuous) and likelihood of left ventricular improvement at 6-months

	**Adjusted odds ratio (95% CI)**	**Interaction p-value**
Viable (per 10% LV increase)	1.00 (0.91 to 1.10)	-
PCI group	1.01 (0.89 to 1.16)	0.79
OMT group	0.99 (0.87 to 1.12)	
Non-viable (per 10% LV increase)	0.80 (0.70 to 0.91)	-
PCI group	0.84 (0.70 to 1.01)	0.44
OMT group	0.76 (0.64 to 0.91)	

CI – confidence interval. LV – left ventricle. OMT – optimal medical therapy. PCI – percutaneous coronary intervention

© 2023 Perera D et al. *JAMA Cardiology.*

Table e7C - Interaction between treatment assignment, viability characteristics (continuous) and likelihood of left ventricular improvement at 12-months

	**Adjusted odds ratio (95% CI)**	**Interaction p-value**
Viable (per 10% LV increase)	1.09 (1.00 to 1.19)	-
PCI group	1.09 (0.96 to 1.23)	0.93
OMT group	1.10 (0.96 to 1.24)	
Non-viable (per 10% LV increase)	0.77 (0.67 to 0.87)	-
PCI group	0.80 (0.66 to 0.96)	0.51
OMT group	0.74 (0.62 to 0.87)	
Scar (per 10% LV increase)	0.62 (0.50 to 0.78)	-
PCI group	0.66 (0.48 to 0.91)	0.60
OMT group	0.59 (0.43 to 0.79)	

CI – confidence interval. LV – left ventricle. OMT – optimal medical therapy. PCI – percutaneous coronary intervention

© 2023 Perera D et al. *JAMA Cardiology.*

eReferences.

1. Steg PG, Greenlaw N, Tardif JC et al. Women and men with stable coronary artery disease have similar clinical outcomes: insights from the international prospective CLARIFY registry. *Eur Heart J*. 2012;33(22):2831-40.
2. Daly C, Clemens F, Lopez Sendon JL et al. Gender differences in the management and clinical outcome of stable angina. *Circulation*. 2006;113(4):490-8.
3. Stolfo D, Uijl A, Vedin O et al. Sex-Based Differences in Heart Failure Across the Ejection Fraction Spectrum: Phenotyping, and Prognostic and Therapeutic Implications. *JACC Heart Fail*. 2019;7(6):505-15.
4. Bragazzi NL, Zhong W, Shu J et al. Burden of heart failure and underlying causes in 195 countries and territories from 1990 to 2017. *Eur J Prev Cardiol*. 2021;28(15):1682-90.
5. Lawson CA, Zaccardi F, Squire I et al. 20-year trends in cause-specific heart failure outcomes by sex, socioeconomic status, and place of diagnosis: a population-based study. *Lancet Public Health.* 2019;4(8):e406-e20.
6. Colantonio LD, Gamboa CM, Richman JS et al. Black-White Differences in Incident Fatal, Nonfatal, and Total Coronary Heart Disease. *Circulation*. 2017;136(2):152-66.
7. Zaman MJ, Philipson P, Chen R et al. South Asians and coronary disease: is there discordance between effects on incidence and prognosis? *Heart*. 2013;99(10):729-36.
8. Morgan H, Sinha A, Mcentegart M, Hardman SM, Perera D. Evaluation of the causes of sex disparity in heart failure trials. *Heart*. 2022.
9. Velazquez EJ, Lee KL, Deja MA et al. Coronary-artery bypass surgery in patients with left ventricular dysfunction. *N Engl J Med*. 2011;364(17):1607-16.

© 2023 Perera D et al. *JAMA Cardiology.*
