# Supplementary material for: Viability and Outcomes With Revascularization or Medical Therapy in Ischemic Ventricular Dysfunction: A Prespecified Secondary Analysis of the REVIVED-BCIS2 Trial
Source: JAMA Cardiol. 2023 Oct 25;8(12):1154–61. doi: 10.1001/jamacardio.2023.3803 (PMC10600721; doi:10.1001/jamacardio.2023.3803)
Supplement: Supplement 3. — Nonauthor Collaborators [file jamacardiol-e233803-s003.pdf]

Supplemental Online Content: Nonauthor Collaborators

*Indicates required information. Only first name, last name, and suffix will appear in PubMed.

## *Group Name(s): REVIVED-BCIS2 Investigators

| ***First Name and Middle Initial(s)** | ***Last Name** | ***Suffix (eg, Jr, III)** | Academic Degrees | Institution | Location (city, state/province, country) | Role or Contribution, eg, chair, principal investigator | Group (if more than 1 Group listed in the byline) and/or Subgroup (eg, Steering Committee) |
|---|---|---|---|---|---|---|---|
| Gerry | Carr-White | | | Guy’s & St Thomas’ Hospital | London, UK | | |
| Antonis | Pavlidis | | | Guy’s & St Thomas’ Hospital | London, UK | | |
| Simon | Redwood | | | Guy’s & St Thomas’ Hospital | London, UK | | |
| Brian | Clapp | | | Guy’s & St Thomas’ Hospital | London, UK | | |
| Aldo | Rinaldi | | | Guy’s & St Thomas’ Hospital | London, UK | | |
| Haseeb | Rahman | | | Guy’s & St Thomas’ Hospital | London, UK | | |
| Natalia | Briceno | | | Guy’s & St Thomas’ Hospital | London, UK | | |
| Sophie | Arnold | | | Guy’s & St Thomas’ Hospital | London, UK | | |
| Amy | Raynsford | | | Guy’s & St Thomas’ Hospital | London, UK | | |
| Margaret | McEntegart | | | Golden Jubilee National Hospital | Glasgow, UK | | |
| Stuart | Watkins | | | Golden Jubilee National Hospital | Glasgow, UK | | |
| Aadil | Shaukat | | | Golden Jubilee National Hospital | Glasgow, UK | | |
| Paul | Rocchiccioli | | | Golden Jubilee National Hospital | Glasgow, UK | | |
| Louise | Cowan | | | Golden Jubilee National Hospital | Glasgow, UK | | |
| Ceri | Davies | | | Bart's Heart Centre | London, UK | | |
| Elliott | Smith | | | Bart's Heart Centre | London, UK | | |
| Bhavik | Modi | | | Bart's Heart Centre | London, UK | | |
| Jehangir | Din | | | Royal Bournemouth Hospital | Bournemouth, UK | | |
| Jonathon | Hinton | | | Royal Bournemouth Hospital | Bournemouth, UK | | |
| Jonathan | Blaxill | | | Leeds General Infirmary | Leeds, UK | | |
| Abdul | Mozid | | | Leeds General Infirmary | Leeds, UK | | |
| Michelle | Anderson | | | Leeds General Infirmary | Leeds, UK | | |
| Simon | Walsh | | | Royal Victoria Hospital | Belfast, UK | | |
| Mark | Spence | | | Royal Victoria Hospital | Belfast, UK | | |
| Patricia | Glover | | | Royal Victoria Hospital | Belfast, UK | | |
| Richard | Edwards | | | Freeman Hospital | Newcastle, UK | | |
| Mohaned | Egred | | | Freeman Hospital | Newcastle, UK | | |
| Hannah | Stevenson | | | Freeman Hospital | Newcastle, UK | | |
| George | Amin-Youssef | | | King's College Hospital | London, UK | | |

*Indicates required information. Only first name, last name, and suffix will appear in PubMed.

| ***First Name and Middle Initial(s)** | ***Last Name** | ***Suffix (eg, Jr, III)** | Academic Degrees | Institution | Location (city, state/province, country) | Role or Contribution, eg, chair, principal investigator | Group (if more than 1 Group listed in the byline) and/or Subgroup (eg, Steering Committee) |
|---|---|---|---|---|---|---|---|
| Ajay | Shah | | | King's College Hospital | London, UK | | |
| Jonathan | Byrne | | | King's College Hospital | London, UK | | |
| Nilesh | Pareek | | | King's College Hospital | London, UK | | |
| Jonathan | Breeze | | | King's College Hospital | London, UK | | |
| Anthony | Gershlick | | | Glenfield Hospital | Leicester, UK | | |
| Andrew | Ladwiniec | | | Glenfield Hospital | Leicester, UK | | |
| Iain | Squire | | | Glenfield Hospital | Leicester, UK | | |
| Donna | Alexander | | | Glenfield Hospital | Leicester, UK | | |
| Julian | Strange | | | Bristol Heart Institute | Bristol, UK | | |
| Tom | Johnson | | | Bristol Heart Institute | Bristol, UK | | |
| Angus | Nightingale | | | Bristol Heart Institute | Bristol, UK | | |
| Laura | Gallego | | | Bristol Heart Institute | Bristol, UK | | |
| James | Spratt | | | St George's Hospital | London, UK | | |
| Claudia | Cosgrove | | | St George's Hospital | London, UK | | |
| Rupert | Williams | | | St George's Hospital | London, UK | | |
| Sam | Firoozi | | | St George's Hospital | London, UK | | |
| Pitt | Lim | | | St George's Hospital | London, UK | | |
| Dwayne | Conway | | | Pinderfields Hospital | Wakefield, UK | | |
| Peter | Swoboda | | | Pinderfields Hospital | Wakefield, UK | | |
| Paul | Brooksby | | | Pinderfields Hospital | Wakefield, UK | | |
| James | Cotton | | | New Cross Hospital | Wolverhampton, UK | | |
| Richard | Horton | | | New Cross Hospital | Wolverhampton, UK | | |
| Stella | Metherell | | | New Cross Hospital | Wolverhampton, UK | | |
| Kai | Hogrefe | | | Kettering General Hospital | Kettering, UK | | |
| Adrian | Cheng | | | Kettering General Hospital | Kettering, UK | | |
| Sian | Sidgwick | | | Kettering General Hospital | Kettering, UK | | |
| Tim | Lockie | | | Royal Free Hospital | London, UK | | |
| Niket | Patel | | | Royal Free Hospital | London, UK | | |
| Roby | Rakhit | | | Royal Free Hospital | London, UK | | |
| Fozia | Ahmed | | | Manchester Royal Infirmary | Manchester, UK | | |
| Cara | Hendry | | | Manchester Royal Infirmary | Manchester, UK | | |

Supplemental Online Content: Nonauthor Collaborators

*Indicates required information. Only first name, last name, and suffix will appear in PubMed.

| ***First Name and Middle Initial(s)** | ***Last Name** | ***Suffix (eg, Jr, III)** | Academic Degrees | Institution | Location (city, state/province, country) | Role or Contribution, eg, chair, principal investigator | Group (if more than 1 Group listed in the byline) and/or Subgroup (eg, Steering Committee) |
|---|---|---|---|---|---|---|---|
| Farzin | Fath-Ordoubadi | | | Manchester Royal Infirmary | Manchester, UK | | |
| Douglas | Frazer | | | Manchester Royal Infirmary | Manchester, UK | | |
| Mamas | Mamas | | | Manchester Royal Infirmary | Manchester, UK | | |
| Miles | Behan | | | Edinburgh Royal Infirmary | Edinburgh, UK | | |
| Alan | Japp | | | Edinburgh Royal Infirmary | Edinburgh, UK | | |
| Nicholas | Jenkins | | | Sunderland Royal Hospital | Sunderland, UK | | |
| Sam | McClure | | | Sunderland Royal Hospital | Sunderland, UK | | |
| Karen | Martin | | | Sunderland Royal Hospital | Sunderland, UK | | |
| Eltigani | Abdelaal | | | Wythenshawe Hospital | Manchester, UK | | |
| Jaydeep | Sarma | | | Wythenshawe Hospital | Manchester, UK | | |
| Sanjay | Sastry | | | Wythenshawe Hospital | Manchester, UK | | |
| Jo | Riley | | | Wythenshawe Hospital | Manchester, UK | | |
| Pradeep | Magapu | | | Liverpool Heart and Chest Hospital | Liverpool, UK | | |
| Rod | Stables | | | Liverpool Heart and Chest Hospital | Liverpool, UK | | |
| David | Wright | | | Liverpool Heart and Chest Hospital | Liverpool, UK | | |
| Michael | Mahmoudi | | | Southampton General Hospital | Southampton, UK | | |
| Andrew | Flett | | | Southampton General Hospital | Southampton, UK | | |
| Nick | Curzen | | | Southampton General Hospital | Southampton, UK | | |
| Sam | Gough | | | Southampton General Hospital | Southampton, UK | | |
| Zoe | Nicholas | | | Southampton General Hospital | Southampton, UK | | |
| Andrew | Ludman | | | Royal Devon and Exeter Hospital | Exeter, UK | | |
| Hibba | Kurdi | | | Royal Devon and Exeter Hospital | Exeter, UK | | |
| Sam | Keenan | | | Royal Devon and Exeter Hospital | Exeter, UK | | |
| Kevin | Thorpe | | | Royal Devon and Exeter Hospital | Exeter, UK | | |
| Prithwish | Banerjee | | | University Hospitals Coventry & Warwicl | Coventry, UK | | |
| Luke | Tapp | | | University Hospitals Coventry & Warwicl | Coventry, UK | | |
| Abeesh | Panicker | | | University Hospitals Coventry & Warwicl | Coventry, UK | | |

Supplemental Online Content: Nonauthor Collaborators

*Indicates required information. Only first name, last name, and suffix will appear in PubMed.

| ***First Name and Middle Initial(s)** | ***Last Name** | ***Suffix (eg, Jr, III)** | Academic Degrees | Institution | Location (city, state/province, country) | Role or Contribution, eg, chair, principal investigator | Group (if more than 1 Group listed in the byline) and/or Subgroup (eg, Steering Committee) |
|---|---|---|---|---|---|---|---|
| Mark | De Belder | | | James Cook University Hospital | Middlesborough, UK | | |
| Jeet | Thambyrajah | | | James Cook University Hospital | Middlesborough, UK | | |
| Neil | Swanson | | | James Cook University Hospital | Middlesborough, UK | | |
| Neville | Kukreja | | | Lister Hospital | Stevenage, UK | | |
| Mary | Lynch | | | Lister Hospital | Stevenage, UK | | |
| Girish | Viswanathan | | | Derriford Hospital | Plymouth, UK | | |
| Elaine | Jones | | | Derriford Hospital | Plymouth, UK | | |
| Sarah | Norman | | | Derriford Hospital | Plymouth, UK | | |
| Helen | Routledge | | | Worcestershire Acute Hospitals | Worcester, UK | | |
| Jasper | Trevelyan | | | Worcestershire Acute Hospitals | Worcester, UK | | |
| Nick | Pegge | | | Worthing Hospital | Worthing, UK | | |
| Sukhbir | Dhamrajit | | | Worthing Hospital | Worthing, UK | | |
| Tim | Wells | | | Salisbury District Hospital | Salisbury, UK | | |
| Manas | Sinha | | | Salisbury District Hospital | Salisbury, UK | | |
| Gavin | Galasko | | | Blackpool Victoria Hospital | Blackpool, UK | | |
| Christopher | Cassidy | | | Blackpool Victoria Hospital | Blackpool, UK | | |
| Tim | Edwards | | | Dorset County Hospital | Blackpool, UK | | |
| Javed | Iqbal | | | Dorset County Hospital | Blackpool, UK | | |
| Fraser | Witherow | | | Dorset County Hospital | Blackpool, UK | | |
| Kaeng | Lee | | | Birmingham Heartlands Hospital | Birmingham, UK | | |
| James | Beattie | | | Birmingham Heartlands Hospital | Birmingham, UK | | |
| Mike | Pitt | | | Birmingham Heartlands Hospital | Birmingham, UK | | |
| Julian | Gunn | | | Northern General Hospital | Sheffield, UK | | |
| Abdallah | Al-Mohammad | | | Northern General Hospital | Sheffield, UK | | |
| Helen | Denney | | | Northern General Hospital | Sheffield, UK | | |
| Huw | Griffiths | | | Queen Alexandra Hospital | Portsmouth, UK | | |
| Paul | Kalra | | | Queen Alexandra Hospital | Portsmouth, UK | | |
| Tim | Gray | | | Royal Oldham Hospital | Oldham, UK | | |
| Jolanta | Sobolewska | | | Royal Oldham Hospital | Oldham, UK | | |
| Steve | Ramcharitar | | | Great Western Hospital | Swindon, UK | | |
| Laura | McCafferty | | | Great Western Hospital | Swindon, UK | | |

Supplemental Online Content: Nonauthor Collaborators

*Indicates required information. Only first name, last name, and suffix will appear in PubMed.

| ***First Name and Middle Initial(s)** | ***Last Name** | ***Suffix (eg, Jr, III)** | Academic Degrees | Institution | Location (city, state/province, country) | Role or Contribution, eg, chair, principal investigator | Group (if more than 1 Group listed in the byline) and/or Subgroup (eg, Steering Committee) |
|---|---|---|---|---|---|---|---|
| Thomas | Martin | | | Ninewells Hospital | Dundee, UK | | |
| John | Irving | | | Ninewells Hospital | Dundee, UK | | |
| Zaid | Iskandar | | | Ninewells Hospital | Dundee, UK | | |
| Jason | Glover | | | Basingstoke Hospital | Basingstoke, UK | | |
| James | Beynon | | | Basingstoke Hospital | Basingstoke, UK | | |
| Maurice | Pye | | | York Hospital | York, UK | | |
| Simon | Megarry | | | York Hospital | York, UK | | |
| Paul | Das | | | North Wales Cardiac Centre | Rhyl, UK | | |
| Chris | Bellamy | | | North Wales Cardiac Centre | Rhyl, UK | | |
