# Supplementary material for: Viability and Outcomes With Revascularization or Medical Therapy in Ischemic Ventricular Dysfunction: A Prespecified Secondary Analysis of the REVIVED-BCIS2 Trial
Source: JAMA Cardiol. 2023 Oct 25;8(12):1154–61. doi: 10.1001/jamacardio.2023.3803 (PMC10600721; doi:10.1001/jamacardio.2023.3803)
Supplement: Supplement 4. — Data Sharing Statement [file jamacardiol-e233803-s004.pdf]

Data Sharing Statement

Perera. Viability and Outcomes With Revascularization or Medical Therapy in Ischemic Ventricular Dysfunction. *JAMA Cardiol*. Published October 25, 2023.
doi:10.1001/jamacardio.2023.3803

Data
Data available: Yes
Data types: Deidentified participant data
How to access data: De-identified data will be made available one year from the end of the trial on submission of a structured request to the corresponding author and completion of a signed data sharing agreement.
When available: beginning date: 09-30-2024

Supporting Documents
Document types: None

Additional Information
Who can access the data: De-identified data will be made available one year from the end of the trial on submission of a structured request to the corresponding author and completion of a signed data sharing agreement.
Types of analyses: As above
Mechanisms of data availability: As above
Any additional restrictions: As above
